# Unconventional mechanism and selectivity of the Pd-catalyzed C–H bond lactonization in aromatic carboxylic acid

**DOI:** 10.1038/s41467-022-27986-6

**Published:** 2022-01-14

**Authors:** Li-Ping Xu, Shaoqun Qian, Zhe Zhuang, Jin-Quan Yu, Djamaladdin G. Musaev

**Affiliations:** 1 Cherry L. Emerson Center for Scientific Computation, Department of Chemistry, Emory University, 1521 Dickey Drive, Atlanta, GA 30322 USA; 2 School of Chemistry and Chemical Engineering, Shandong University of Technology, Zibo, 255000 China; 3 Department of Chemistry, The Scripps Research Institute, 10550 North Torrey Pines Road, La Jolla, CA 92037 USA

**Keywords:** Density functional theory, Catalytic mechanisms

## Abstract

The search for more effective and highly selective C–H bond oxidation of accessible hydrocarbons and biomolecules is a greatly attractive research mission. The elucidating of mechanism and controlling factors will, undoubtedly, help to broaden scope of these synthetic protocols, and enable discovery of more efficient, environmentally benign, and highly practical new C–H oxidation reactions. Here, we reveal the stepwise intramolecular S_N_2 nucleophilic substitution mechanism with the rate-limiting C–O bond formation step for the Pd(II)-catalyzed C(sp^3^)–H lactonization in aromatic 2,6-dimethylbenzoic acid. We show that for this reaction, the direct C–O reductive elimination from both Pd(II) and Pd(IV) (oxidized by O_2_ oxidant) intermediates is unfavorable. Critical factors controlling the outcome of this reaction are the presence of the η^3^-(π-benzylic)–Pd and K^+^–O(carboxylic) interactions. The controlling factors of the benzylic vs ortho site-selectivity of this reaction are the: (a) difference in the strains of the generated lactone rings; (b) difference in the strengths of the η^3^-(π-benzylic)–Pd and η^2^-(π-phenyl)–Pd interactions, and (c) more pronounced electrostatic interaction between the nucleophilic oxygen and K^+^ cation in the ortho-C–H activation transition state. The presented data indicate the utmost importance of base, substrate, and ligand in the selective C(sp^3^)–H bond lactonization in the presence of C(sp^2^)–H.

## Abstract (web summary)

Methods to functionalize inert C–H bonds are a critical focus of synthetic organic chemistry. In this work the authors use computations and experiments to uncover the mechanisms of palladium-catalysed C–H lactonizations in aromatic carboxylic acids, and explain the origin of an observed preference for functionalization of a C(sp3)–H bond over a C(sp2)–H bond in a recent report.

## Introduction

The transition-metal-catalyzed and directing group assisted selective conversion of “inert” C–H bonds to functional C–C and C-heteroatom bonds has emerged as a powerful synthetic strategy^1–29^. Despite of numerous advances in this field of the chemical research, the search for more effective and highly selective C–H functionalization reactions utilizing of more important and accessible substrates, and inexpensive ligands, as well as proceeding via a green and sustainable ways, is a greatly attractive research mission^30^. In this context, catalytic synthesis of lactones, which are highly valuable intermediates in the synthesis of various natural products, via the C–H bond oxidation of accessible hydrocarbons has attracted significant attention^31–34^.

**Fig. 1:**
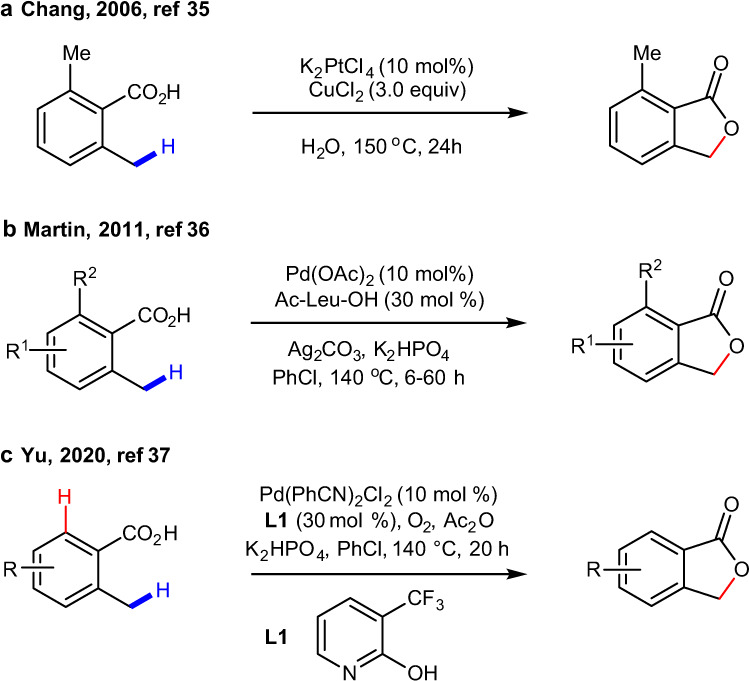
Transition metal-catalyzed direct C–H lactonization reactions. **a** Pt-catalyzed lactonization reported by Lee and Chang^35^; **b** Pd-catalyzed, MPAA ligand and Ag salt-assisted, lactonization by Novák et al.^36^, and **c** Pd-catalyzed, pyridine ligand and O_2_-assisted lactonization by Qian et al.^37^.

Recently, several seminal direct C–H lactonization reactions, both aromatic^35–37^ (i.e., benzolactones) and aliphatic^38^ (i.e., β-lactones) substrates, have been reported. For instance (see Fig. 1), Chang and coworkers have developed a Pt(II)-catalyzed benzylic C–H lactonization with three equivalents of CuCl_2_ as the oxidant^35^. Martin and coworkers have reported a palladium-catalyzed C(sp^3^)–H lactonization with mono-protected amino acid ligand and stoichiometric silver(I) as the oxidant^36^. Very recently, Yu and coworkers have reported a Pd(II)-catalyzed C(sp^3^)–H lactonization of o-methyl benzoic acids using molecular oxygen as the sole oxidant^37^. Amazingly, in the reaction reported by Yu and coworkers the stronger C(sp^3^)–H bond lactonization is achieved in the presence of the relatively weaker C(sp^2^)–H bond.

Despite of applicability of the developed synthetic protocols to the late-stage functionalization^39^, the mechanisms of these reactions, as well as the roles of the utilized catalysts, ligands, and oxidants remain to be elucidated. The acquiring of fundamental knowledge will, undoubtedly, help to broaden scope of these synthetic protocols, and enable discovery of more efficient, environmentally benign, and highly practical new C–H lactonization reactions. Nowadays, state-of-art computations have proven to be a powerful strategy in study of mechanistic details of complex reactions^40–45^.

In this work, we launch a joint experimental and computational study on the mechanism and controlling factors of the Pd(II)-catalyzed C(sp^3^)–H lactonization of aromatic 2,6-dimethylbenzoic acid (BA) by utilizing pyridone as a ligand, and O_2_ as an oxidant. We elaborate the origin of the observed unusually selective C(sp^3^)–H vs. C(sp^2^)–H bond lactonization. In our study, we use 2,6-dimethylbenzoic acid as a substrate, Pd(PhCN)_2_Cl_2_ as an initial catalyst, pyridone as a ligand, and K_2_HPO_4_ as a base.

## Results and discussion

### Active catalyst, true nature of substrate, and the C–H bond activation step

**Fig. 2:**
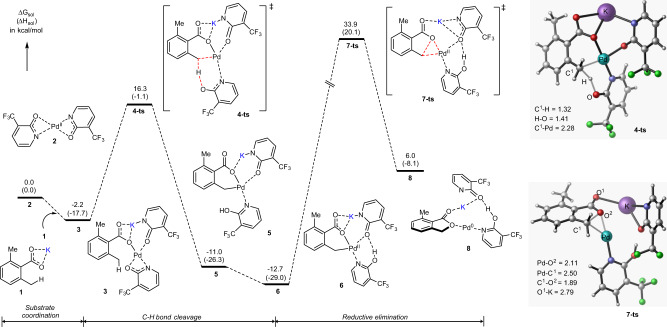
The calculated free energy surface. Energy profile for the substrate coordination, C–H bond activation, and direct C–O reductive elimination steps for the benzolactonization reaction, and geometries of the key transition states (bond distances are given in Å).

At first, we used computations to identify the true nature of the used substrate under the reported reaction conditions. We found that the generation of the potassium carboxylate (**1**, Fig. 2), by the reaction of 2,6-dimethyl benzoic acid with the potassium phosphate, is thermodynamically favorable (see Supplementary Information, below called as SI). Therefore, we identified the potassium salt **1** as a true substrate of this reaction.

Next, we have elucidated the nature of the catalytic active species under the reported experimental conditions. In general, under the reported reaction conditions, the active catalysts can be complexes Pd(**L1)**_2_ (**2**), Pd(**L1)** (**2_1L**), Pd(**L1)**_**3**_^**–**^ (**2_3L**), Pd(**L1)P** (**2_LP**), and Pd(**P)**_**2**_ (here, **L1** is the deprotonated pyridone ligand, and **P** = KHPO_4_^–^). Since the previous experiments^37^ have indicated no reaction without the ligand, we, confidently, can eliminate complex Pd(**P)**_**2**_ as the active catalyst. In this paper, we have calculated the key steps of the titled reaction by utilizing of all above listed complexes as the active catalysts. Comparision of the calculated critical kinetic and thermodynamic parameters of these reaction steps (see the SI for details) allowed us to establish complex Pd(**L1)**_2_ (**2**), as the most favorable active catalyst.

Having identified the true natures of substrate (**1**) and active catalyst (**2**) of the reaction given in Fig. 1c, the next step of the reaction can be expected to be the complexation, i.e., formation of complex **3**: this process is calculated to be exergonic by 2.2 kcal/mol (Fig. 2). We found that the benzylic C(sp^3^)–H bond activation in complex **3** occurs via the concerted metalation-deprotonation (CMD) transition state **4-ts**, and leads to palladacycle **5**. In course of this CMD process, one of the pyridone ligands serves as the deprotonating reagent. As seen in Fig. 2, overall reaction **3 → 4-ts → 5** occurs with a free energy barrier of 18.5 kcal/mol and is exergonic by 8.8 kcal/mol. Later, intermediate **5** easily isomerizes to its thermodynamically favorable conformer **6** with an intramolecular H-bond.

### C–O bond formation step

The next step of the reaction is the C–O bond formation from the Pd(II)-complex **6**. Previous experimental and computational studies have demonstrated a complexity of the C–O bond formation from the Pd(II), Pt(IV), and Pd(IV)-complexes^46–53^. In 2001, Goldberg and coworkers concluded that the C(sp^3^)–O bond formation from the Pt(IV)-complex, likely, involves the OR^–^ dissociation and following nucleophilic addition^46^. In 2005, Sanford and coworkers provided evidence that from the Pd(IV)-complex, the C(aryl)–O bond formation might undergo via a concerted three-centered transition state^47^. In contrast, the following stereochemistry analysis by Stahl, Liu and coworkers^48^ of the Pd(IV)-catalyzed C(alkyl)–O bond formation provided support for the S_N_2 nucleophilic substitution mechanism. Later, the proposed S_N_2 mechanism in the Pd(IV)-catalyzed C(sp^3^)–O bond formation was confirmed by Sanford and coworkers^49,50^ and by our group^51^. In 2009, Ribas and coworkers proposed a concerted C(sp^2^)–O bond formation from the bimetallic Pd(III)/Pd(III) intermediate^52^. However, all these early reports described the C–O bond formation from the high-valent group 10 transition metals [i.e., Pt(IV), Pd(IV), and Pd(III)]. In the literature, there are only rare mechanistic studies on the C–O bond formation from the Pd(II)-complexes. In 2011, Hartwig and coworkers reported the C(sp^3^)–O reductive elimination from the bisphosphine-ligated Pd(II) aryloxide complexes and established an ionic mechanism of the reaction^54^. For the Pd(II)-catalyzed C(sp^3^)–H lactonization with stoichiometric silver(I) oxidant^36^, Martin and coworkers have proposed a concerted reductive elimination from the Pd(II)-intermediate, but they did not rule out a stepwise mechanism involving the dissociation of the carboxylate ligand and following C–O bond formation steps.

Armed with these examples, here we investigated several possible mechanistic scenarios of the C–O bond formation from the Pd(II)-complex **6**. One of them is the direct C–O reductive elimination. Our calculations show that direct C–O reductive elimination from the Pd(II)-complex **6** requires a prohibitively high free energy barrier (46.6 kcal/mol, at the transition state **7-ts**, Fig. 2).

The next mechanistic scenario, investigated in this paper, is the Pd(II) to Pd(IV) oxidation by the oxidant (i.e., O_2_ molecule), and then the C–O bond reductive elimination from the Pd(IV)-complex. It is widely accepted that reductive elimination from the high-valent transition metal complex should be a facile process^21,55^. Since in the reported experiment^37^ O_2_ was used as the sole oxidant, here, we investigated the Pd(II) to Pd(IV) oxidation by O_2_ in intermediate **6**, and its impact to the calculated C–O bond formation energy barrier. All our efforts have shown that O_2_ does not oxidize Pd(II) to Pd(IV) in intermediate **6**. The calculated reductive elimination transition states from the O_2_-oxidized Pd(IV) intermediates bear dramatically high free energy barriers (see SI for details). Coordination of O_2_ to pre-reaction complex **6** or to transition state **7-ts** does not reduce the C–O bond formation energy barrier (see the SI). We also validated several other mechanistic pathways, such as (a) the dissociation of one of the pyridone ligands before the reductive elimination, and (b) coordination of H_2_O_2_ (which may be generated in situ under the utilized reaction conditions) then C–O reductive elimination. Our calculations have shown that both of these pathways are highly unfavorable (see SI for details). Thus, the presented calculations show that O_2_ does not facilitate the C–O bond reductive elimination in the studied Pd-catalyzed and pyridone ligand-enabled lactonization of 2,6-dimethyl benzoic acid.

**Fig. 3:**
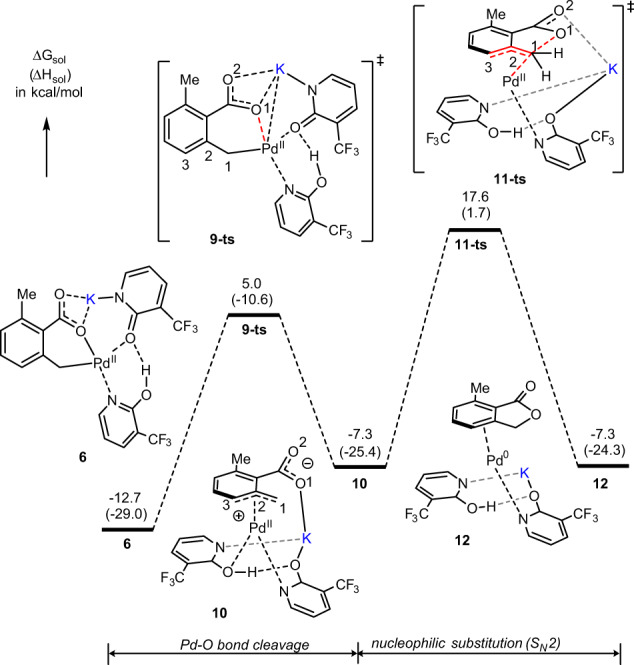
The calculated free energy profile. Energy profile for the stepwise C–O formation process: Pd–O bond cleavage and nucleophilic substitution.

**Fig. 4:**
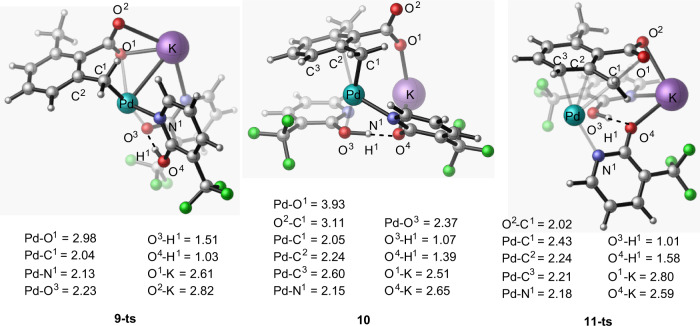
The calculated geometry structures of the critical transition states and intermediates. The optimized structures of **9-ts**, **10**, and **11-ts** along with their important geometry parameters (in Å).

Then, we turned our attention to the stepwise S_N_2 nucleophilic substitution mechanism of the titled reaction. Calculations show that (Fig. 3) the first step of the proposed stepwise S_N_2 nucleophilic substitution mechanism, i.e., the Pd–O(O=)CR bond cleavage, occurs with a 17.7 kcal/mol free energy barrier at the transition state **9-ts** and leads to intermediate **10** with the η^3^–(π-benzylic)-Pd(II) interaction. This process is endergonic by 5.4 kcal/mol (see Figs. 3 and 4).

Analyses show that in **10** (Fig. 4), the π-benzylic ligand coordination to the Pd-center (in an asymmetric η^3^ fashion with the Pd–C^1^ = 2.05 Å, Pd–C^2^ = 2.24 Å, and Pd–C^3^ = 2.60 Å bonds) stabilizes the cationic palladium center. In this complex, (i) the emerging negative charge is localized on the leaving carboxylic oxygen (O^1^), and (ii) electrostatic interaction between the potassium ion and O^1^-center (O^1^-K = 2.51 Å) provides additional stability to the intermediate **10**.

The subsequent step of the investigated S_N_2 nucleophilic substitution mechanism is nucleophilic attack of another carboxylate oxygen (O^1^) to benzylic carbon (C^1^) to form the C^1^–O^1^ bond at the transition state **11-ts** (see Figs. 3 and 4). This step of the reaction has a barrier of 30.3 kcal/mol, relative to **6**, and is the rate-determining step of the studied stepwise S_N_2 nucleophilic substitution mechanism. Comparing to the direct C–O bond reductive elimination from both intermediate **6** (i.e., at the transition state **7-ts**, ΔG^≠^ = 46.6 kcal/mol) and the O_2_-coordinated Pd-complex, (O_2_)–**6**, (i.e., at the transition state **7-ts-b**, ΔG^≠^ = 52.8 kcal/mol, see SI), the stepwise C–O bond formation via the S_N_2 nucleophilic substitution in intermediate **6** (at **11-ts**, ΔG^≠^ = 30.3 kcal/mol), is highly favorable. Therefore, we conclude that (i) the Pd(II)-catalyzed and pyridone ligand-enabled lactonization of 2,6-dimethyl benzoic acid may proceed via the stepwise S_N_2 nucleophilic substitution pathway, and (ii) the critical controlling factors of this reaction are the: (ii.a) η^3^–(π-benzylic)–Pd, i.e., π-cation, interaction, and (ii.b) electrostatic interaction between the potassium ion and emerging negative charge of the leaving carboxylic oxygen (O^1^).

As seen in Fig. 3, the formation of the Pd-coordinated lactone, **12**, is exergonic by 7.3 kcal/mol relative to the reactants (**1** + **2**), and endergonic by 5.4 kcal/mol relative to complex **6**. As that could be hypothesized, the next steps of the reaction from **12** could be (i) release of the lactone and formation of the Pd(0) complex, and (ii) oxidation of Pd(0) to Pd(II) by the O_2_ oxidant. Since these processes were previously studied in details^56^, here we did not investigate those, again.

One should emphasize that the above-presented C–O bond formation via the stepwise S_N_2 nucleophilic substitution for the Pd(II)-catalyzed and pyridone ligand-enabled lactonization of 2,6-dimethyl benzoic acid is different from that previously reported by Hartwig and coworkers in the bisphosphine-ligated Pd(II) aryloxide complexes^54^. Indeed, in the present study, (a) the C(benzylic)-O(carboxylate) bond is formed to generate lactone, and (b) it occurs via an “intramolecular” fashion as the carboxylate is assembled at the ortho position of the benzyl group. In contrast, in the reaction reported by Hartwig and coworkers, (a) the C(benzylic)-O(aryloxide) is formed to produce ether, and (b) it occurs via an “intermolecular” fashion, where the ArO^−^ entirely dissociates from the reaction center followed by nucleophilic attack from the other prochiral face^54^. In general, our findings are consistent with the previously made hypotheses by Martin and coworkers^36^ in the study of the Pd(II)-catalyzed C(sp^3^)–H lactonization with mono-protected amino acid ligand and stoichiometric silver(I) as the oxidant.

### Experimental validation of the stepwise mechanism

Thus, the above-presented computations have identified the stepwise intramolecular S_N_2 nucleophilic substitution mechanism of the Pd(II)-catalyzed and pyridone ligand-enabled lactonization of 2,6-dimethyl benzoic acid with the rate-determining C–O formation step. We have shown that two factors, critically impacting the outcome of this reaction, are the (a) η^3^–(π-benzylic)–Pd, i.e., π-cation, interaction, and (b) electrostatic interaction between the potassium ion and emerging negative charge of the leaving carboxylic oxygen (O^1^), i.e., the M^+^–O^1^(O=)CR interaction. To validate these predictions, we have performed several control experiments.

### Solvent effect

One would expect that polarity and amount of the used solvent, as well as the nature of alkali metal (M) would impact the critical M^+^–O^1^(O=)CR interaction, consequently, the outcome of the reaction. Indeed, it would be expected that polar solvent molecules will solvate the M^+^ cation, consequently, reduce the stabilizing M^+^–O^1^(O=)CR interaction in intermediate **10** and transition state **11-ts**. To test this hypothesis, at first, we investigated the geometry and energy parameters of intermediates and transition states of the rate-determining C–O formation step with an explicit solvent (PhCl) molecule. Briefly, we found that while the presence of the PhCl–K^+^, i.e., π-cation, the interaction does slightly stabilize the **6**, **10**, and **11-ts** structures, and increases the overall activation free energy barrier of the C–O formation by 2.9 kcal/mol, but does not impact our final conclusions (see the SIs for more details).

Then, we performed a control experiment with different amounts of H_2_O water (see SI). Briefly, we found that the addition of different amounts of H_2_O to utilized standard experimental conditions decreases the yield of the reaction. Of course, the presence of water molecules and its quantity could impact to the reaction outcome via multiple different ways, one of them is the solvation of the M-cation: thus, these experimental findings, indirectly, support the above presented computational predictions, and the proposed stepwise mechanism of the Pd(II)-catalyzed and pyridone ligand-enabled lactonization of 2,6-dimethyl benzoic acid.

### Alkali metal (M) effect

**Fig. 5:**
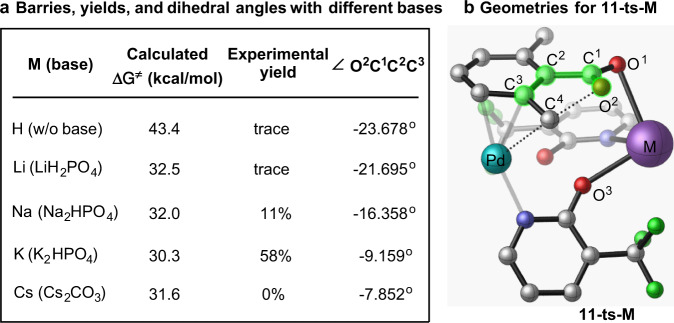
Different alkali metal base effects. **a** Calculated activation free energy barriers, experimental yields, and dihedral angles with different bases, and **b** Geometry of the key transition state **11-ts-M** (The hydrogen atoms are omitted for clarity).

Another factor that will impact the M^+^–O^1^(O=)CR interaction is the nature of the used alkali metal. Previously^37^, we have reported that without base or with the Na-base (Na_2_HPO_4_) the yields of the reaction are trace amount or 11%, respectively (Fig. 5). Here, we have calculated the rate-limiting C–O bond formation barriers for the reactions without base and with the Na-base, and found them to be 43.4 and 32.0 kcal/mol, respectively. Both of these values are larger than 30.3 kcal/mol, calculated for the reaction with the K_2_HPO_4_ base.

Close analyses of the calculated rate-limiting transition state structure **11-ts** show that the structural motif of the M^+^–O^1^(O=)CR fragment is critically dependent on the size (e.g., atomic radii) of the used alkali metal. If the size of M is too small, the carboxylate (-C^1^O^1^O^2^) group rotates down and allows O^1^ to effectively interact with M^+^. In its turn, this causes the O^2^ atom of carboxylate (which is nucleophile in the C–O bond formation) to bend out of the aromatic plane (manifested in the calculated [O^2^C^1^C^2^C^3^] dihedral angle), consequently, retard the O^2^–C^1^ bond formation: the calculated [O^2^C^1^C^2^C^3^] dihedral angle reduces by reducing the size of M, i.e., via M = H (−23.678^o^) > M = Na (−16.358^o^) > M = K (−9.159^o^). This trend is consistent with the order of reduction of energy barrier as M = H (43.4 kcal/mol) > M = Na (32.0 kcal/mol) > M = K (30.3 kcal/mol).

Based on these analyses, we hypothesized that the yield of the Pd(II)-catalyzed and pyridone ligand-enabled lactonization of 2,6-dimethyl benzoic acid with Li-base should be even smaller than 11% (that reported for the reaction with Na-base), but it should be significantly larger with Cs-base. The calculated free energy barrier of 32.5 kcal/mol, and associated [O^2^C^1^C^2^C^3^] dihedral angle of −21.695° for the reaction with Li-base (see Fig. 5) are fully consistent with this prediction. Gratifyingly, our following experiments with LiH_2_PO_4_ as a base showing only a trace amount of lactone formation fully validated the prediction of computation for the reaction with Li-base.

In contrast, our extensive calculations of the key steps, i.e., **6** → **9-ts** → **10** → **11-ts** steps, of the Pd(II)-catalyzed and pyridone ligand-enabled lactonization of 2,6-dimethyl benzoic acid with Cs-base revealed a very complex nature of this reaction. Namely, although the calculated [O^2^C^1^C^2^C^3^] dihedral angle is smaller than that for the K-base case, the calculated rate-limiting nucleophilic C–O bond formation free energy barrier is larger for the reaction with Cs-base compared to the K-base case (Fig. 5).

Close examination of transition state structure **11-ts-Cs** (see SI) revealed a strong electrostatic interaction between the Cs^+^ and carboxylate (-C^1^O^1^O^2^) group, where Cs^+^ interacts not only with O^1^ atom (which is the case in other above-reported transition state structures with different alkali metals) but also with the inner O^2^ atom (which is a nucleophile in the C–O bond formation). The latter interaction (i.e., the Cs^+^ and nucleophilic O^2^ interaction) decreases the nucleophilicity of the O^2^-center and results in the higher C–O bond formation energy barrier. Thus, based on these computational findings we summarize that the Pd(II)-catalyzed and pyridone ligand-enabled lactonization of 2,6-dimethyl benzoic acid with Cs-base is going to be less efficient than that with the K-base because of the presence of the Cs^+^ and nucleophilic O^2^ interaction. Consistent with the computational prediction, our following experiments with Cs-base showed no lactone formation (see SI for details).

### Site-selectivity: benzylic C(sp^3^)–H vs. ortho C(sp^2^)–H bond lactonization

Above presented stepwise intramolecular S_N_2 nucleophilic substitution mechanism of the Pd(II)-catalyzed and pyridone ligand-enabled lactonization of 2,6-dimethyl benzoic acid also enables us to elucidate the origin of the previously reported site-selective, C(sp^3^)–H (benzylic) vs C(sp^2^)–H (ortho), bond lactonization in substrate **1b** (o-Toluic acid)^37^. For this purpose, we have calculated both the benzylic and ortho C–H bond lactonization pathways of the Pd(II)-catalyzed and pyridone ligand-enabled lactonization of **1b** (see SI for details).

**Fig. 6:**
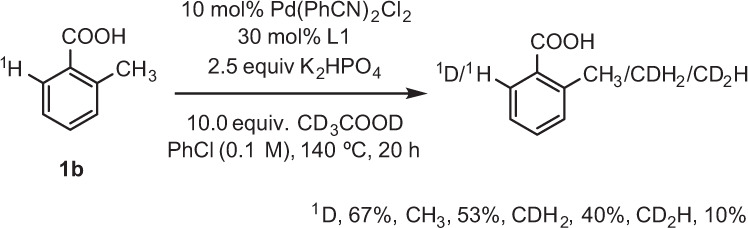
Deuterium-labeling study. Deuterium-labeling experiment for the Pd(II)-catalyzed C–H lactonization (Note, this reaction was performed in the absence of O_2_).

Briefly, these calculations show (see SI for details) that both C(sp^3^)–H (benzylic) and C(sp^2^)–H (ortho) bond activation in **1b** is reversible, and the following C–O bond formation is a rate-limiting step. To validate these computational findings, we performed the deuterium labeling experiments (see Fig. 6 and SIs for more details) without the oxidant O_2_. By adding 10.0 Equiv of CD_3_COOD into the reaction mixture we observed the H/D exchange at both benzylic and ortho positions. These experimental findings confirmed the reversible nature of the C–H bond activation in **1b**, as predicted by the computation.

**Fig. 7:**
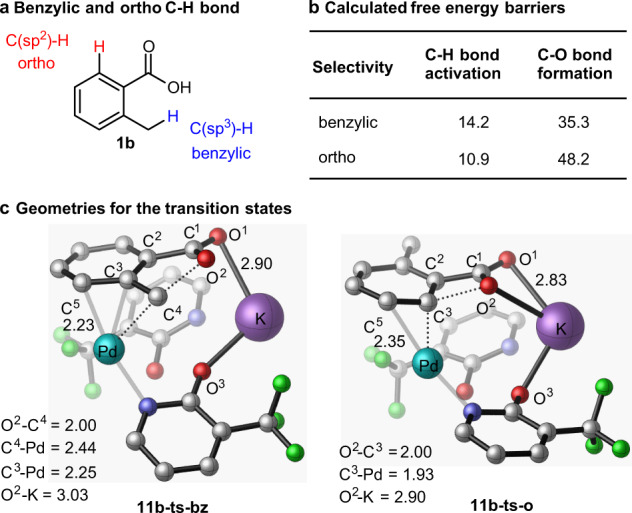
Selectivity in C–H bond lactonization. **a** Benzylic and ortho C–H bonds; **b** Calculated free energy barrier for C–H bond activation and C–O bond formation (in kcal/mol), and **c** geometries for the key transition states (hydrogen atoms are omitted for clarity and bond distances are in Å).

The presented calculations (see Fig. 7 and SIs for more details) also show that the benzylic C(sp^3^)–H bond activation requires a slightly larger energy barrier than ortho C(sp^2^)–H bond activation (14.2 kcal/mol vs 10.9 kcal/mol, respectively). However, the following, and rate-limiting, C(benzylic)–O bond formation occurs by smaller energy barrier than the competing C(ortho)–O bond formation (35.3 kcal/mol vs. 48.2 kcal/mol, respectively). Analyses of the associated C–O formation transition states **11b-ts-bz** and **11b-ts-o** show that the major reasons for the calculated difference in the C(sp^3^)–O and C(sp^2^)–O bond formation energy barriers are: (a) the difference in the strains of the generated five-membered (upon the benzylic C(sp^3^)–H bond lactonization) and four-membered (upon the ortho C(sp^2^)–H bond lactonization) lactone rings; (b) difference in the strengths of the η^3^-(π-benzylic)–Pd and η^2^-(π-phenyl)–Pd interactions; and (c) the more pronounced electrostatic interaction between the nucleophilic O^2^ atom and K^+^ cation in the transition state **11b-ts-o** which reduces nucleophilicity of the O^2^ atom. Furthermore, these analyses also show the presence of the pyridone-Pd(cationic) and pyridone-K^+^ cation interactions in the key C–O formation transition states. Nevertheless, the above presented computational findings are fully consistent with the previous experiments^37^ establishing the preferential benzylic C–H lactonization in **1b**.

**Fig. 8:**
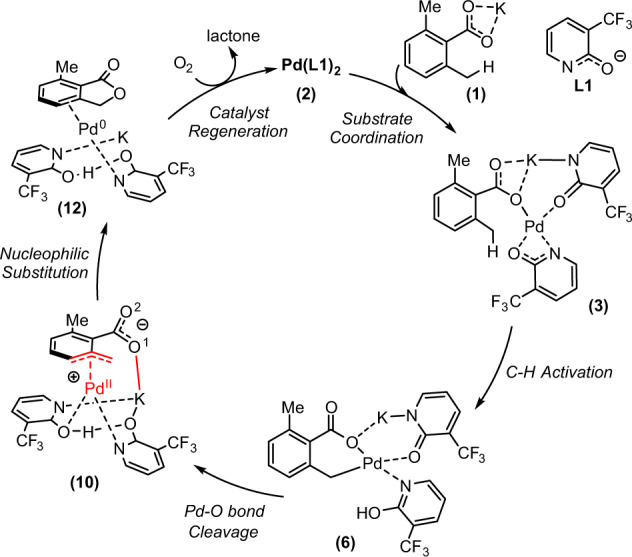
The proposed mechanism. The mechanism of the C(sp^3^)–H lactonization in aromatic 2,6-dimethyl benzoic acid.

In summary, the above presented computational and experimental studies on the Pd(II)-catalyzed and pyridone ligand-enabled C(sp^3^)–H lactonization in aromatic 2,6-dimethyl benzoic acid show that: (1) Under the reported reaction conditions, the true substrate of the reaction is potassium carboxylate, **1**, and the active catalytic species is complex Pd**L1**_2_ (**2**) (where **L1** is the deprotonated pyridone ligand); (2) This reaction proceeds via the stepwise S_N_2 nucleophilic substitution pathway (Fig. 8), critical steps of which are the (a) Pd–O^1^(O^2^=)CR bond cleavage, leading to the intermediate with the η^3^–(π-benzylic)–Pd interaction, and (b) nucleophilic attack of the carboxylate oxygen (O^2^) to the benzylic carbon to form the C–O bond. The C–O bond formation is a rate-limiting step; (3) Critical factors controlling the outcome of this reaction are the (a) η^3^-(π-benzylic)–Pd, and (b) potassium ion and negatively charged carboxylic oxygen (O^1^) interactions; (4) The used molecular O_2_ plays a critical role for catalyst re-generation by oxidizing of Pd(0)-product complex to the Pd(II) active species, and (5) The origins of the observed the C(sp^3^)–H (benzylic) vs C(sp^2^)–H (ortho) site-selective C–H bond lactonization in o-Toluic acid is the (a) difference in the strains of the generated lactone rings; (b) difference in the strengths of the η^3^-(π-benzylic)–Pd and η^2^-(π-phenyl)–Pd interactions; and (c) more pronounced electrostatic interaction between the nucleophilic carboxylic oxygen (O^2^) and K^+^ cation in the ortho transition state.

Thus, the above presented computational data clearly indicate of utmost importance of base, substrate, and ligand in functionalization of the C(sp^3^)–H bond in the presence of C(sp^2^)–H bond, which is a long-standing challenge of synthetic chemistry.

## Methods

### Computational details

Optimization of all reported structures and frequency calculations were performed using the Gaussian-09 suite of programs^57^ at the B3LYP-D3/[6-31G(d,p) + Lanl2dz (Pd, Cs)] level of theory (called as a B3LYP-D3/BS1 approach) with the corresponding Hay–Wadt effective core potential^58,59^ for Pd and Cs. Here, we used the B3LYP density functional^60–62^ with Grimme’s empirical dispersion correction (D3)^63^. Frequency analyses were used to characterize each minimum and each transition state (TS) structure, and to obtain thermal and entropy corrections to the reported thermodynamic parameters. Intrinsic reaction coordinate calculations were performed for all TSs to ensure their true nature. Solvent effects were incorporated in geometry optimizations and frequency calculations with the SMD^64^ solvent model. PhCl is used as the solvent. In some cases, we used the explicit solvent approach. We have also performed single-point energy calculations by utilizing the larger [6–311++G(d,p) + SDD (Pd, Cs)] basis sets (called as a BS2) at the B3LYP-D3/BS1 optimized structures (here, we call this approach as the B3LYP-D3/BS2//B3LYP-D3/BS1 approach). The reported final free energies are the sum of the B3LYP-D3/BS2 calculated electronic energies and the B3LYP-D3/BS1 calculated thermal corrections. 3D geometries were prepared using CYLView software^65^. All reported thermodynamics were obtained by utilizing the standard conditions (i.e., 298 K and 1 atm). The presented energies are presented Δ*G*(Δ*H*) (in kcal/mol) unless otherwise stated.

### Experimental procedure

The general procedure for C(sp^3^)–H lactonization is as follows: o-methyl benzoic acid (0.2 mmol), Pd(PhCN)_2_Cl_2_ (10 mol%, 7.6 mg), L1 (30 mol%, 9.8 mg), and K_2_HPO_4_ (2.5 equiv, 87.0 mg) were weighed in open air and placed in a 12 × 75 mm borosilicate test tube (5 mL). Ac_2_O (2.0 equiv, 18.9 μL) and PhCl (0.1 M, 2.0 mL) were added. The reaction heated to 140 °C and pressurized to 400 psi using a 5% oxygen in nitrogen gas mix for 20 h. Afterward, the reaction mixture was cooled to room temperature and vented. The crude mixture was diluted with EtOAc and then filtered with Celite. The filtrate was concentrated in vacuo, and the resulting mixture was purified by column chromatography or pTLC using hexane/EtOAc as the eluent. Full experiment details can be found in the Supplementary Information.

## Supplementary information

Supplementary Information

## Data Availability

All data supporting the findings of this study are available within the article and Supplementary Information files, or from the corresponding authors upon request.

## Code availability

Here, we used only the commercial codes (see references).
