# Supplementary material for: Unconventional mechanism and selectivity of the Pd-catalyzed C–H bond lactonization in aromatic carboxylic acid
Source: Nat Commun. 2022 Jan 14;13:315. doi: 10.1038/s41467-022-27986-6 (PMC8760335; doi:10.1038/s41467-022-27986-6)
Supplement: Supplementary file 1 — Supplementary Information [file 41467_2022_27986_MOESM1_ESM.pdf]

Supplementary Information

Unconventional Mechanism and Selectivity in the Pd(II)-Catalyzed C–H Bond Lactonization in Aromatic Carboxylic Acid

Li-Ping Xu[†,§], Shaoqun Qian[‡], Zhe Zhuang[‡], Jin-Quan Yu[*,‡], Djamaladdin G. Musaev[*,†]

[†]Cherry L. Emerson Center for Scientific Computation, and Department of Chemistry, Emory University, 1521 Dickey Drive, Atlanta, GA, 30322, United States
[‡]Department of Chemistry, The Scripps Research Institute, 10550 North Torrey Pines Road, La Jolla, California 92037, United States
[§] School of Chemistry and Chemical Engineering, Shandong University of Technology, Zibo, 255000, China

E-mail: dmusaev@emory.edu yu200@scripps.edu

Contents

1. **Supplementary Methods**
   (1) Computational details ········ S2
   (2) General experimental procedure ········ S2
2. **Supplementary Discussion**
   (1) Reaction of *o,o*-dimethyl benzoic acid with K_2HPO_4 ········ S2
   (2) Reaction energies of the formation of **PdL1$_2$** and **PdP$_2$** ········ S3
   (3) Reaction of *o,o*-dimethyl benzoic acid with **PdL1$_2$** and **PdP$_2$**: Substrate coordination and C-H bond activation steps ········ S3
   (4) Activation barrier for the rate-determining S_N2 nucleophilic substitution step with the **PdP$_2$** catalyst ········ S4
   (5) Other possible reductive elimination pathways involving O_2, H_2O_2, and ligand dissociation ········ S5
   (6) Geometry for the transition state **11-ts-Cs** ········ S5
   (7) Reaction pathways for the ortho and benzylic C-H lactonization with **1b** ····· S6
   (8) General procedure for control experiment with different amounts of water ··· S7
   (9) General procedure for reaction with different alkali metal bases ········ S8
   (10) Reaction with cesium base ········ S8
   (11) General procedure for deuterium-labeling experiment ········ S9
   (12) Results with other possible active catalysts ········ S9
   (13) Results for the low-level basis set calculations ········ S12
   (14) Control experiment with different amount of water molecules ……………S13
   (15) Results with the explicit PhCl solvent model ········ S14
   (16) Energetics for all calculated species ········ S15
   (17) Cartesian coordinates for all calculated species ········ S17
3. **Supplementary References** ········ S49

1. **Supplementary Methods**

(1) Computational details

Optimization of all reported structures and frequency calculations were performed using the Gaussian-09 suite of programs[1] at the B3LYP-D3/[6-31G(d,p) + Lanl2dz (Pd, Cs)] level of theory (called as a B3LYP-D3/BS1 approach) with the corresponding Hay-Wadt effective core potential[2,3] for Pd and Cs. Here we used the B3LYP density functional[4-6] with Grimme's empirical dispersion-correction (D3).[7] Frequency analyses were used to characterize each minimum and each transition state (TS) structures, and to obtain thermal and entropy corrections to the reported thermodynamic parameters. Intrinsic reaction coordinate (IRC) calculations were performed for all TSs to ensure their true nature. Solvent effects were incorporated in geometry optimizations and frequency calculations with the SMD[8] solvent model. PhCl is used as the solvent. We have also performed single-point energy calculations by utilizing the larger [6-311++G(d,p) + SDD (Pd, Cs)] basis sets (called as a BS2) at the B3LYP-D3/BS1 optimized structures (here, we call this approach as the B3LYP-D3/BS2//B3LYP-D3/BS1 approach). The reported final free energies (ΔG_{sol}-HL) are the sum of the B3LYP-D3/BS2 calculated electronic energies and the B3LYP-D3/BS1 calculated thermal corrections. 3D geometries were prepared using CYLView software.[9] The presented energies are presented ΔG(ΔH) (in kcal/mol) unless otherwise stated.

(2) General experimental procedure

The general procedure for C(sp^3)-H lactonization is as follows: o-methyl benzoic acid (0.2 mmol), $Pd(PhCN)_2Cl_2$ (10 mol%, 7.6 mg), **L1** (30 mol%, 9.8 mg) and K_2HPO_4 (2.5 equiv, 87.0 mg) were weighed in open air and placed in a 12x75 mm borosilicate test tube (5 mL). Ac_2O (2.0 equiv, 18.9 μL) and PhCl (0.1 M, 2.0 mL) were added. The reaction heated to 140 °C and pressurized to 400 psi using a 5% oxygen in nitrogen gas mix for 20h. Afterwards, the reaction mixture was cooled to room temperature and vented. The crude mixture was diluted with EtOAc and then filtered with Celite. The filtrate was concentrated in vacuo, and the resulting mixture purified by column chromatography or pTLC using hexane/EtOAc as the eluent.

2. **Supplementary Discussion**

(1) Reaction of *o,o*-dimethyl benzoic acid with K_2HPO_4

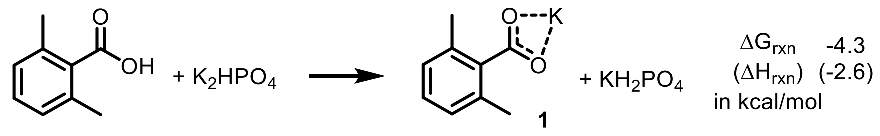

Supplementary Figure 1. Reaction and reaction energies of *o,o*-dimethyl benzoic acid with K_2HPO_4.

(2) Reaction energies of the formation of **PdL1$_2$** and **PdP$_2$**

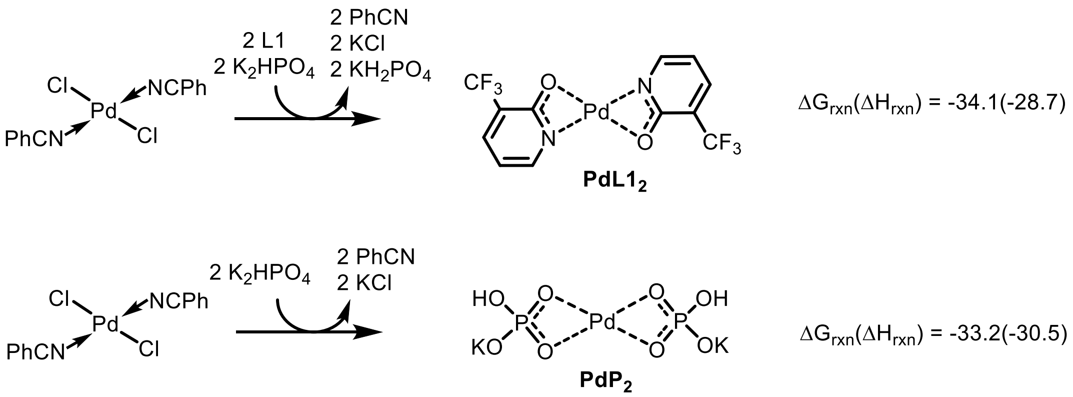

Supplementary Figure 2. Reaction Energies of the Formation of **PdL1$_2$** and **PdP$_2$**.

(3) Reaction of *o,o*-dimethyl benzoic acid with **PdL1$_2$** (Figure 3) and **PdP$_2$** (Figure 4): substrate coordination and C-H bond activation steps

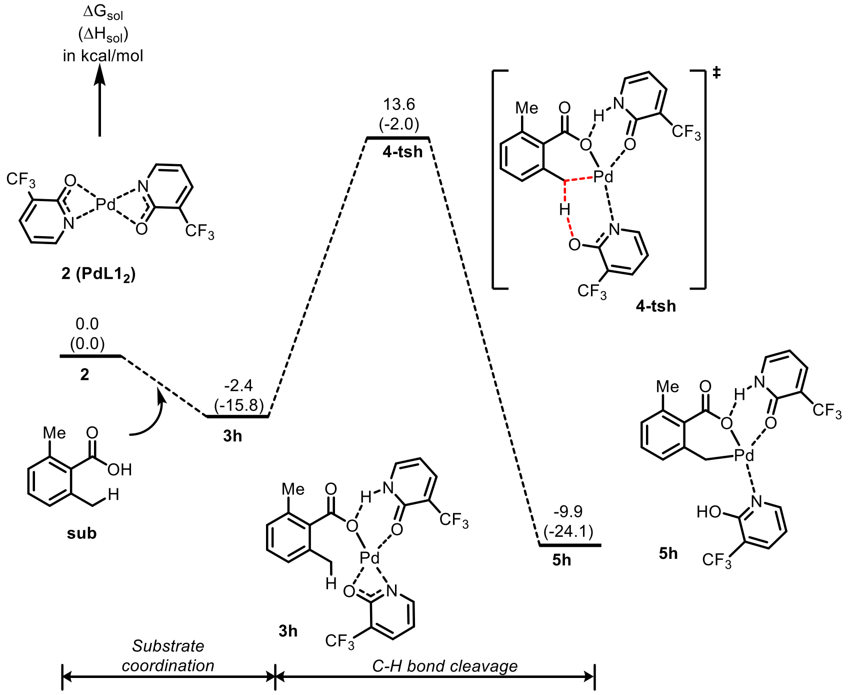

Supplementary Figure 3. Energy profile for the reaction of *o,o*-dimethyl benzoic acid with **PdL1$_2$**.

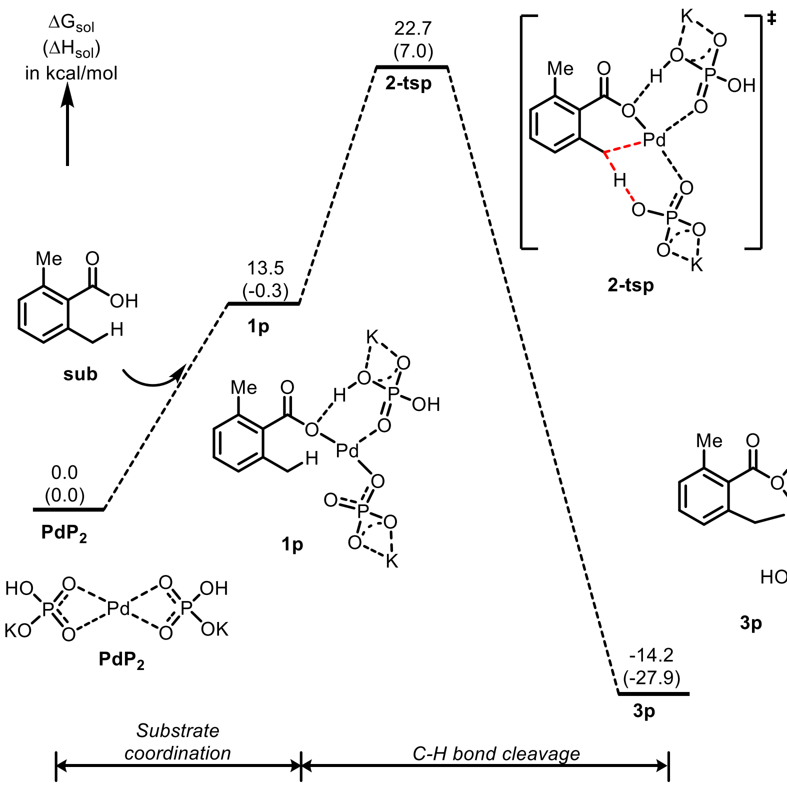

Supplementary Figure 4. Energy profile for the reaction of *o,o*-dimethyl benzoic acid with **PdP₂** (note that the C-H bond activation barrier is higher than that with **PdL1₂**).

(4) Activation barrier for the rate-determining S_N2 nucleophilic substitution step with the **PdP₂** catalyst

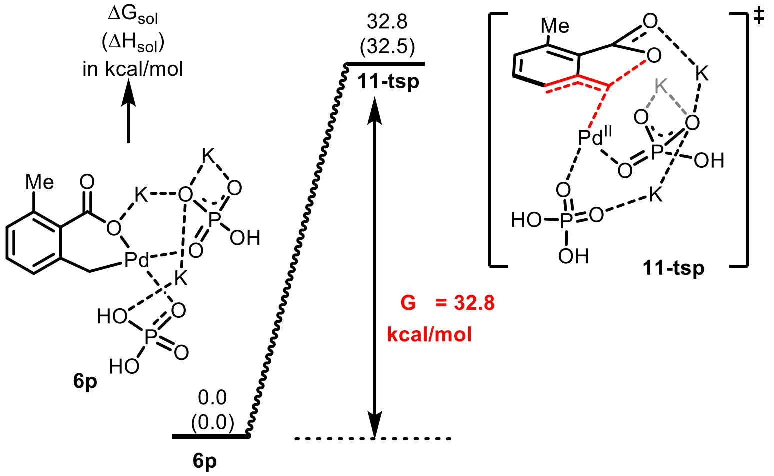

Supplementary Figure 5. Activation barrier for the rate-determining S_N2 nucleophilic substitution step with the **PdP₂** catalyst (note that this rate-determining barrier is higher than that with the **PdL1₂** catalyst, which has a 30.3 kcal/mol barrier).

(5) Other possible reductive elimination pathways involving O_2, H_2O_2, and ligand dissociation

with O_2 coordination (triplet)

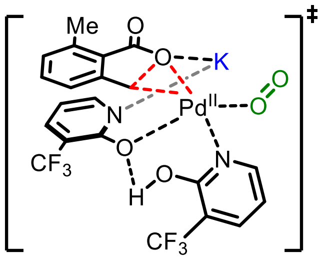

7-ts-a

G = 60.1 kcal/mol

O_2 oxidize Pd(II) to Pd(IV)

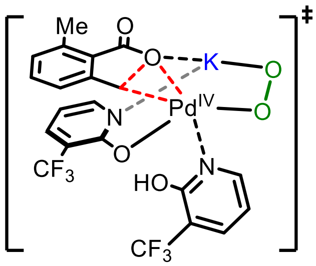

7-ts-b

G = 52.8 kcal/mol

with H_2O_2 coordination

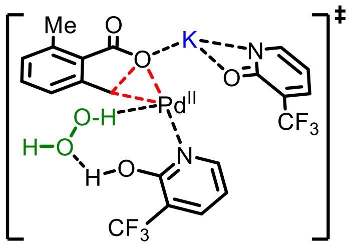

7-ts-c

G = 54.4 kcal/mol

O_2 replaces one LH (triplet)

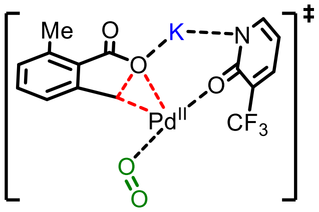

7-ts-d

G = 67.8 kcal/mol

O_2 oxidize Pd(II) to Pd(IV), with one ligand

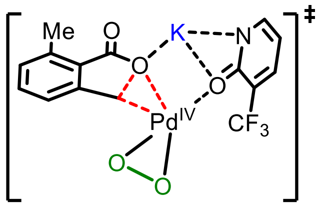

7-ts-e

G = 56.6 kcal/mol

one LH dissociation followed by reductive elimination

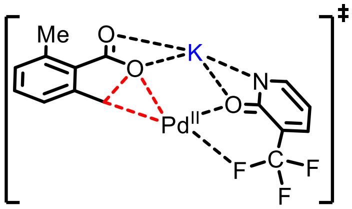

7-ts-f

G = 47.5 kcal/mol

Supplementary Figure 6. Other possible reductive elimination pathways involving O_2, H_2O_2, and ligand dissociation.

(6) Geometry of the transition state **11-ts-Cs**

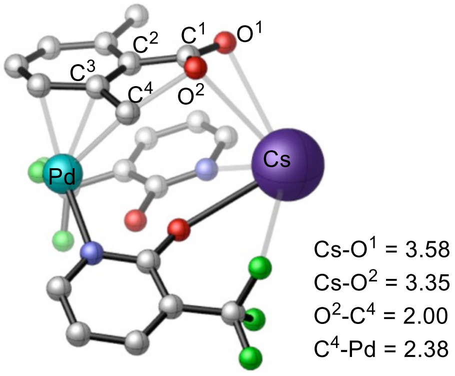

11-ts-Cs

Supplementary Figure 7. Geometry for the transition state **11-ts-Cs** (note that the Cs cation has interactions with both O^1 and O^2 atoms from the carboxylate group).

(7) Reaction pathways for the ortho and benzylic C-H lactonization with **1b**

(a) Potential energy surface for the ortho C-H lactonization with **1b**.

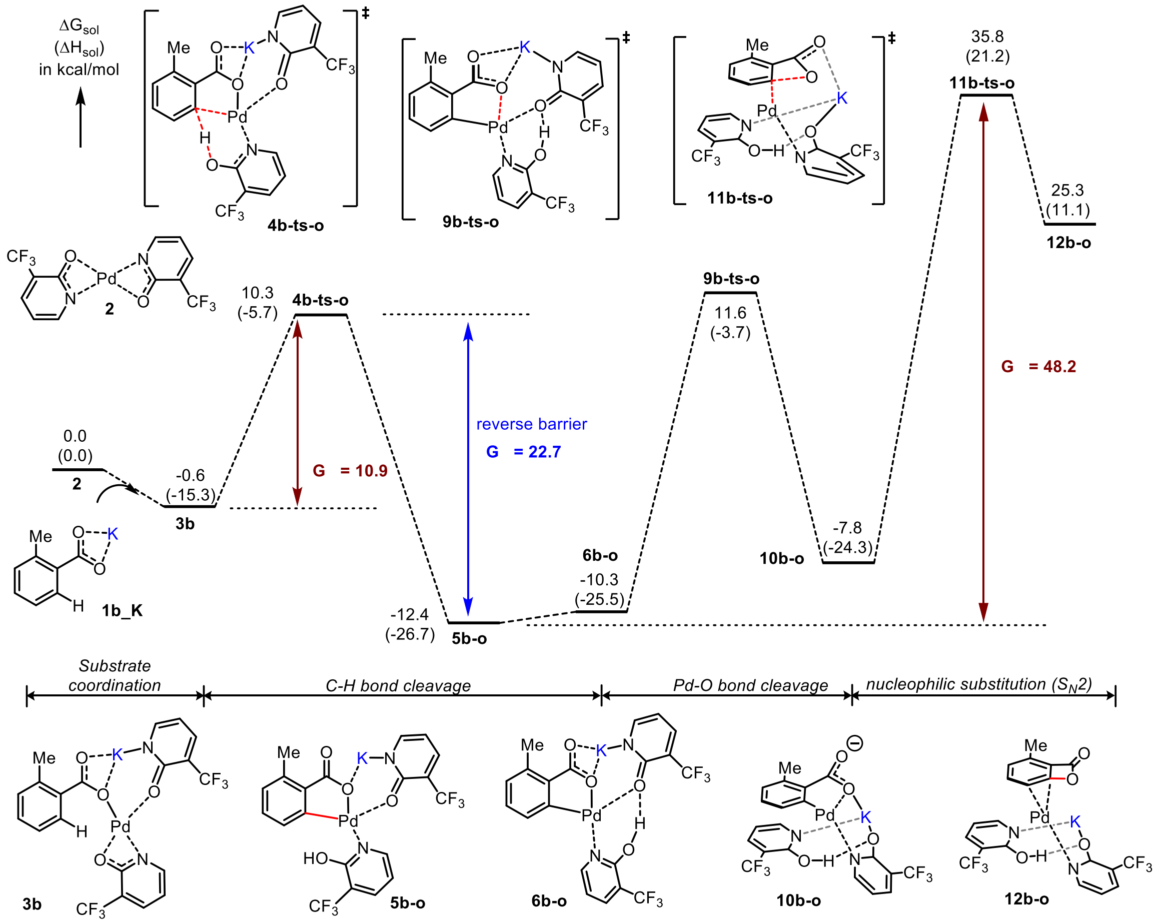

Supplementary Figure 8. Potential energy surface for the ortho C-H lactonization with **1b**.

(b) Potential energy surface for the benzylic C-H lactonization with **1b**.

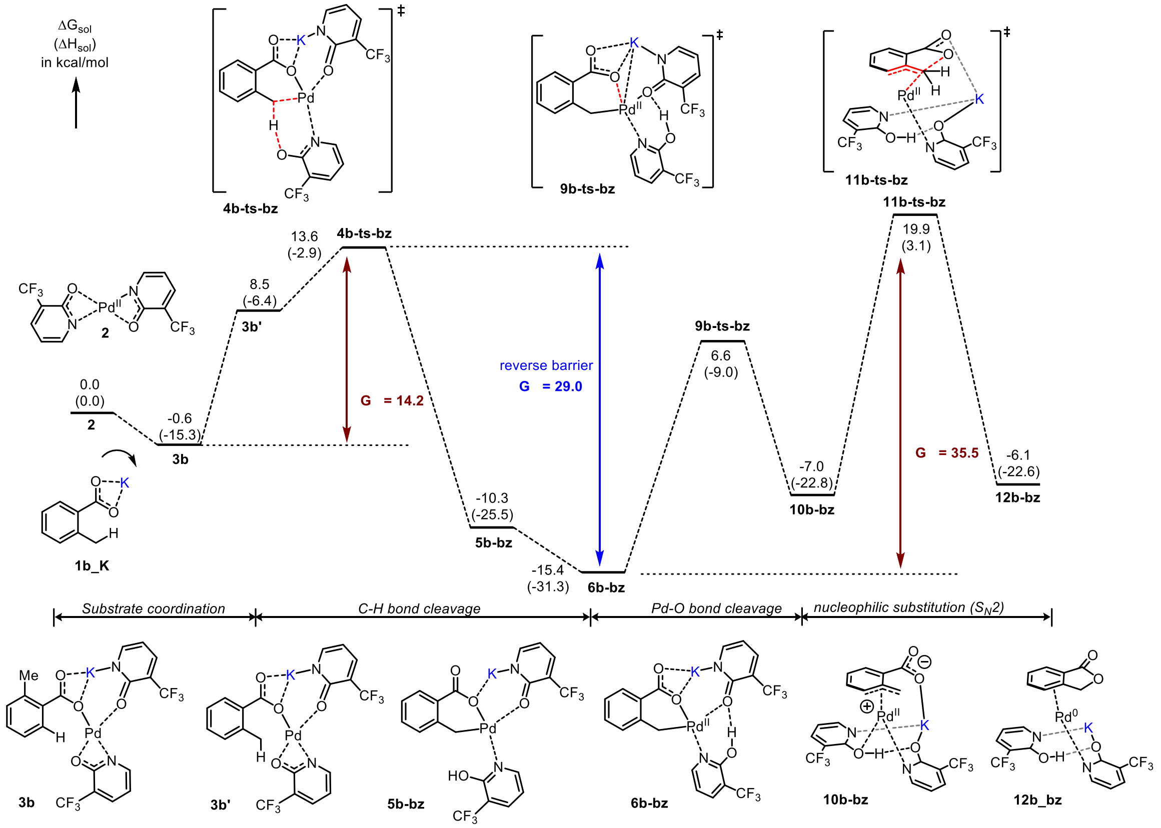

Supplementary Figure 9. Potential energy surface for the benzylic C-H lactonization with **1b**.

(8) General procedure for control experiment with different amounts of water

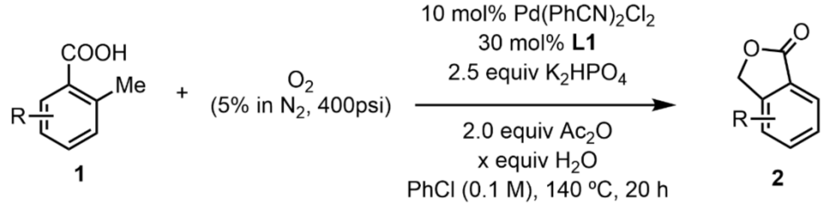

General procedure: Substrate **1** (0.2 mmol), $Pd(PhCN)_2Cl_2$ (10 mol%, 7.6 mg), **L1** (30 mol%, 9.8 mg) and K_2HPO_4 (2.5 equiv, 87.0 mg) were weighed in open air and placed in a 12x75 mm borosilicate test tube (5 mL). Ac_2O (2.0 equiv, 18.9 μL), H_2O (x equiv.) and PhCl (0.1 M, 2.0 mL) were added. The test tube and a stir paddle were placed in a Freeslate Junior with OSR module (Unchained Labs, Pleasanton, CA, USA) equipped with a small volume insert. The reaction was mechanically stirred at 250 rpm, pressurized to 100 psi using a 5% oxygen in nitrogen gas mix, and then heated to 140 °C

with a heating mantle. After one hour at 140 °C, the reaction was further pressurized to 400 psi and stirred for 20 h. Afterwards, the reaction mixture was cooled to room temperature and vented. The crude mixture was diluted with EtOAc and then filtered with Celite. The filtrate was concentrated *in vacuo*, and the resulting mixture purified by column chromatography or pTLC using hexane/EtOAc as the eluent.

(9) General procedure for reaction with different alkali metal bases

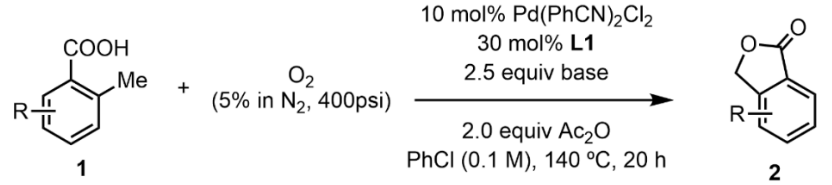

General procedure: Substrate **1** (0.2 mmol), $Pd(PhCN)_2Cl_2$ (10 mol%, 7.6 mg), **L1** (30 mol%, 9.8 mg) and base (2.5 equiv.) were weighed in open air and placed in a 12x75 mm borosilicate test tube (5 mL). Ac_2O (2.0 equiv, 18.9 μL) and PhCl (0.1 M, 2.0 mL) were added. The test tube and a stir paddle were placed in a Freeslate Junior with OSR module (Unchained Labs, Pleasanton, CA, USA) equipped with a small volume insert. The reaction was mechanically stirred at 250 rpm, pressurized to 100 psi using a 5% oxygen in nitrogen gas mix, and then heated to 140 °C with a heating mantle. After one hour at 140 °C, the reaction was further pressurized to 400 psi and stirred for 20 h. Afterwards, the reaction mixture was cooled to room temperature and vented. The crude mixture was diluted with EtOAc and then filtered with Celite. The filtrate was concentrated *in vacuo*, and the resulting mixture purified by column chromatography or pTLC using hexane/EtOAc as the eluent.

(10) Reaction with cesium base

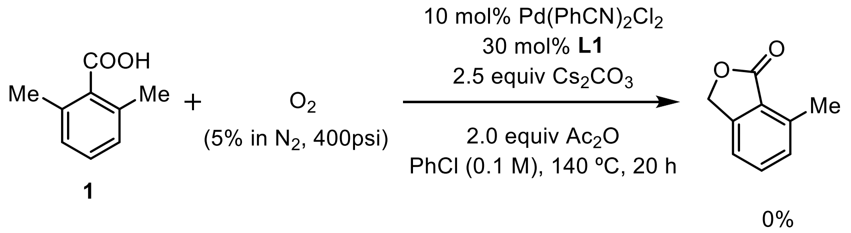

General procedure: Substrate **1** (0.2 mmol), $Pd(PhCN)_2Cl_2$ (10 mol%, 7.6 mg), **L1** (30 mol%, 9.8 mg) and Cs_2CO_3 (2.5 equiv.) were weighed in open air and placed in a 12x75 mm borosilicate test tube (5 mL). Ac_2O (2.0 equiv, 18.9 μL) and PhCl (0.1 M, 2.0 mL) were added. The test tube and a stir paddle were placed in a Freeslate Junior with OSR module (Unchained Labs, Pleasanton, CA, USA) equipped with a small volume insert. The reaction was mechanically stirred at 250 rpm, pressurized to 100 psi using a 5% oxygen in nitrogen gas mix, and then heated to 140 °C with a heating mantle. After one hour at 140 °C, the reaction was further pressurized to 400 psi and stirred for 20 h. Afterwards, the reaction mixture was cooled to room temperature and

vented. The crude mixture was diluted with EtOAc and then filtered with Celite. The filtrate was concentrated *in vacuo*, and the resulting mixture purified by column chromatography or pTLC using hexane/EtOAc as the eluent.

(11) General procedure for deuterium-labeling experiment

(a) With substrate **1b**

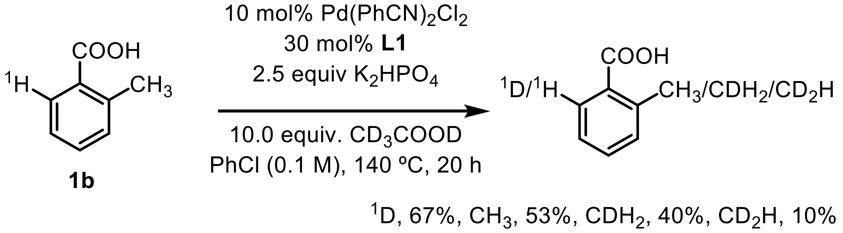

General procedure: 2-methyl benzoic acid (0.2 mmol, 27.2 mg), $Pd(PhCN)_2Cl_2$ (10 mol%, 7.6 mg), **L1** (30 mol%, 9.8 mg) and K_2HPO_4 (2.5 equiv, 87.0 mg) were added to a tube with septum stopper. The tube was evacuated and backfilled with N_2 three times. CD_3COOD (120 uL, 2.0 mmol, 10.0 equiv.) and PhCl (2.0 mL) were added and the reaction mixture was heated to 140 °C for 20 h. After cooling down to room temperature, HCOOH (0.2 mL) was added and stirred for 10 min. The mixture was filtered through a pad of Celite and washed with ethyl acetate, and the solvent was removed under vacuum. The ratio of deuterium was analyzed by the NMR spectrum.

(b) With substrate **1**.

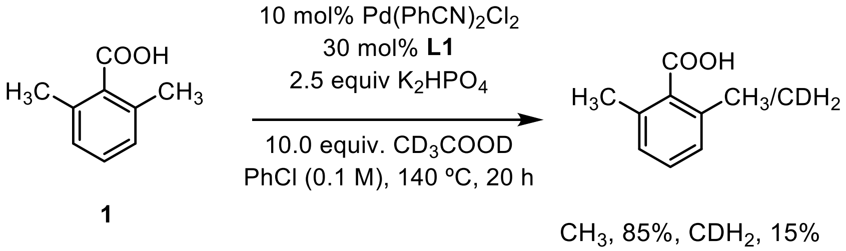

General procedure: 2,6-dimethyl benzoic acid (0.2 mmol, 27.2 mg), $Pd(PhCN)_2Cl_2$ (10 mol%, 7.6 mg), **L1** (30 mol%, 9.8 mg) and K_2HPO_4 (2.5 equiv, 87.0 mg) were added to a tube with septum stopper. The tube was evacuated and backfilled with N_2 three times. CD_3COOD (120 uL, 2.0 mmol, 10.0 equiv.) and PhCl (2.0 mL) were added and the reaction mixture was heated to 140 °C for 20 h. After cooling down to room temperature, HCOOH (0.2 mL) was added and stirred for 10 min. The mixture was filtered through a pad of Celite and washed with ethyl acetate, and the solvent was removed under vacuum. The ratio of deuterium was analyzed by the NMR spectrum.

(12) Results with other possible active catalysts

As we mentioned in the main text, under the reported experimental conditions, the active

catalysts can be complexes Pd(**L1**)$_2$ (**2**), Pd(**L1**) (**2_1L**), Pd(**L1**)$_3^-$ (**2_3L**), Pd(**L1**)**P** (**2_LP**), and Pd(**P**)$_2$ (here, **L1** is the deprotonated pyridone ligand, and **P** = $KHPO_4^-$). Since the previous experiments[37] have indicated no reaction without the ligand, we, confidently, can eliminate complex Pd(**P**)$_2$ as the active catalyst. The data provided in the Figures 4 and 5 for the Pd(**P**)$_2$ as a catalyst, support this experimental finding.

The calculations of thermodynamic stability of the Pd(**L1**) species enabled us to eliminate of this mono-ligated Pd species from the active catalyst list, as well. Indeed, these calculations shown that, under the experimental conditions, Pd(**L1**) converts to other species by:

(a) adding another **L1** ligand to form Pd(**L1**)$_2$: the reaction Pd(**L1**) + **L1** → Pd(**L1**)$_2$ is calculated to be exergonic by 85.2 kcal/mol. In addition, the ligand exchange, i.e. the reaction with the substrate (**1**), Pd(**L1**)$_2$ + **1** → Pd(**L1**)(**1**) + **L1**, is also found to be highly (by 29.8 kcal/mol) endergonic. We should emphasize that the C-H bond activation in Pd(**L1**)(**1**) requires an overall of 62.7 kcal/mol free energy barrier relative to the Pd(**L1**)$_2$ + **1** dissociation limit (see **Figure 10**).

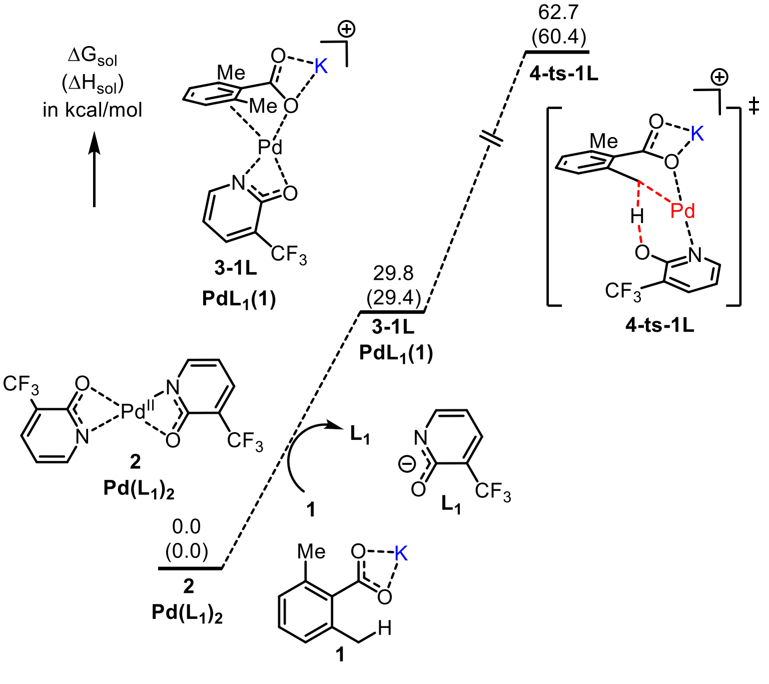

Supplementary Figure 10. The calculated relative energies of the **L1** ligand to substrate (**1**) exchange in Pd(**L1**)$_2$, i.e. reaction Pd(**L1**)$_2$ + **1** → Pd(**L1**)(**1**) + **L1**, and the following C-H bond activation.

(b) by coordinating of one equivalent of **P** to form complex Pd(**L1**)**P.** Reaction Pd(**L1**) + **P** → Pd(**L1**)**P** is calculated to be exergonic by 107.4 kcal/mol. We have studied both the C-H bond activation and the S_N2 nucleophilic substitution steps of the

reaction by utilizing of the resulted Pd(**L1**)**P** complex as the active catalyst (see **Figure 11**). Our results shown that the C-H bond activation requires by 22.7 and 35.8 kcal/mol free energy barriers, for the two pathways involving the pyridone (**4-ts-LP-a**) and phosphate (**4-ts-LP-b**) as the deprotonation base, respectively. From the relatively favorable pathway (i.e., from **4-ts-LP-a**), we have also studied the rate-determining S$_N$2 nucleophilic substitution. Our calculations shown that this process has a barrier of 32.8 kcal/mol (**11-ts-LP**). Comparison to the calculated energies of this reaction with those for the Pd(**L1**)$_2$ complex, in which the C-H bond activation and the S$_N$2 nucleophilic substitution have barriers of 21.1 and 26.9 kcal/mol, respectively (see the main text), allows us to eliminate complex Pd(**L1**)**P (2_LP)** from the active catalyst list.

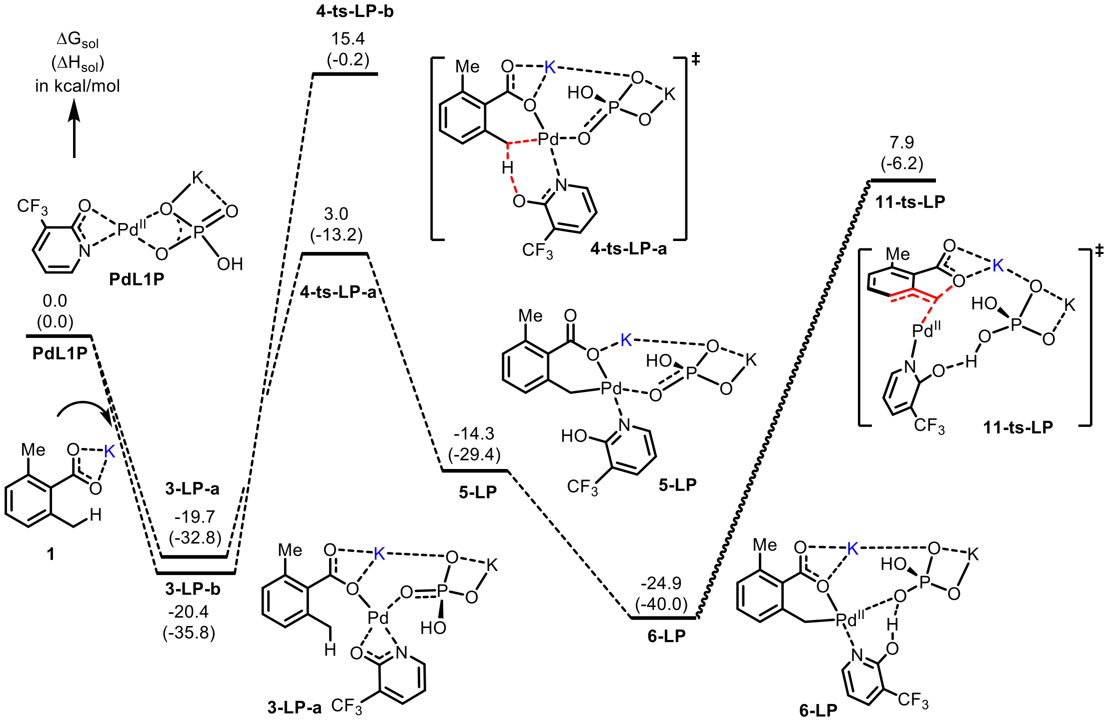

Supplementary Figure 11. The calculated relative energies (in kcal/mol) of the C-H bond activation and following S$_N$2 nucleophilic substitution steps by utilizing of Pd(**L1**)**P, (2_LP)** as the active catalyst.

(c) by adding even two additional **L1** ligands to form Pd(**L1**)$_3^-$ (**2_3L**): The reaction Pd(**L1**)$_2$ + **L1**$^-$ → [Pd(**L1**)$_3$]$^-$ is exergonic endergonic by 1.2 kcal/mol. We have used the resulted anionic Pd(**L1**)$_3^-$ complex as the active catalyst and studied the rate-determining S$_N$2 nucleophilic substitution step. We found that the activation barrier of this reaction is 33.3 kcal/mol (**Figure 12**), which is by 6.4 kcal/mol larger than that with the neutral Pd(**L1**)$_2$ catalyst.

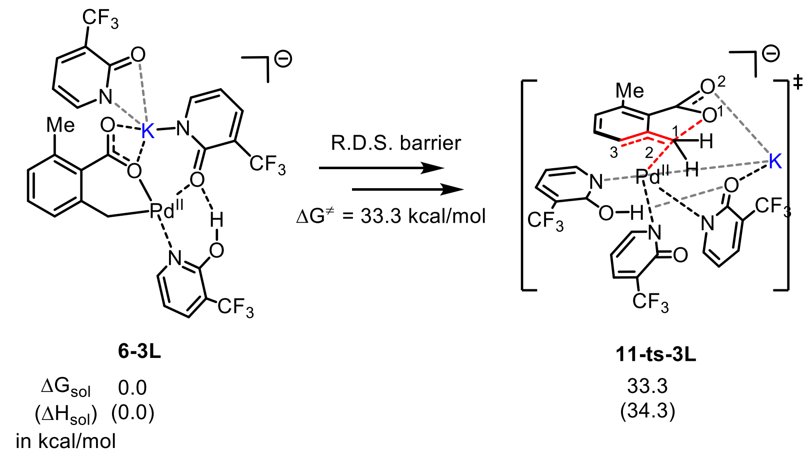

Supplementary Figure 12. The calculated relative energies (in kcal/mol) of the rate-determining S_N2 nucleophilic substitution steps by utilizing of Pd(**L1**)$_3^-$ **(2_3L)** as the active catalyst.

(13) Results for the low-level basis set calculations

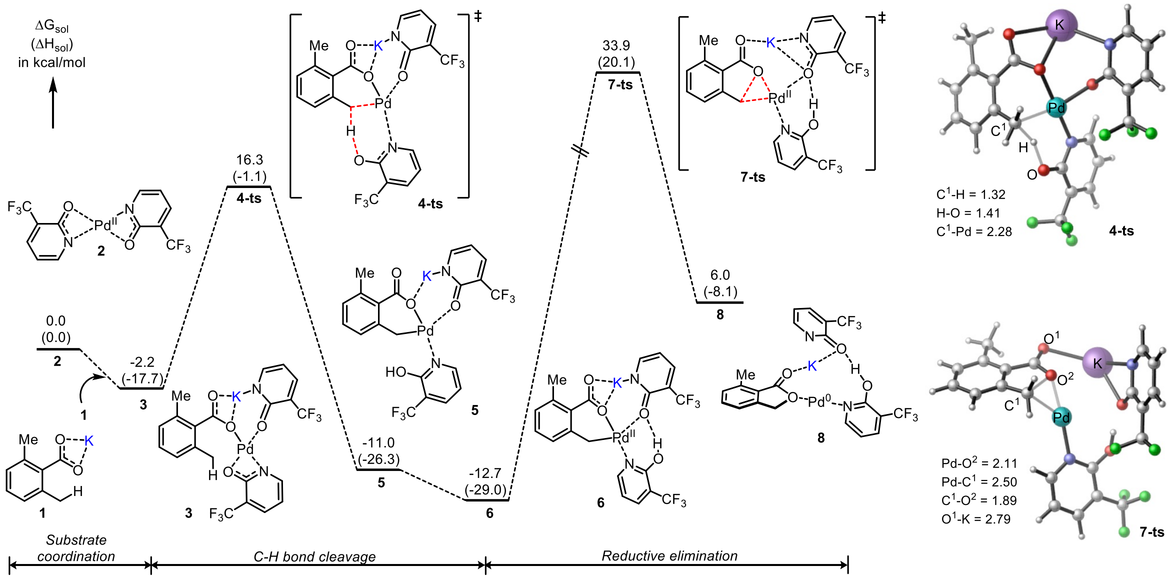

Supplementary Figure 13. Energy profile for the substrate coordination, C-H bond activation, and direct C–O reductive elimination steps of the benzolactonization reaction, and geometries for the key transition states (The energies were obtained with 6-31G(d,p)/Lanl2dz for CHONFK/Pd basis sets (BS1). See the Computational Procedure section for more details).

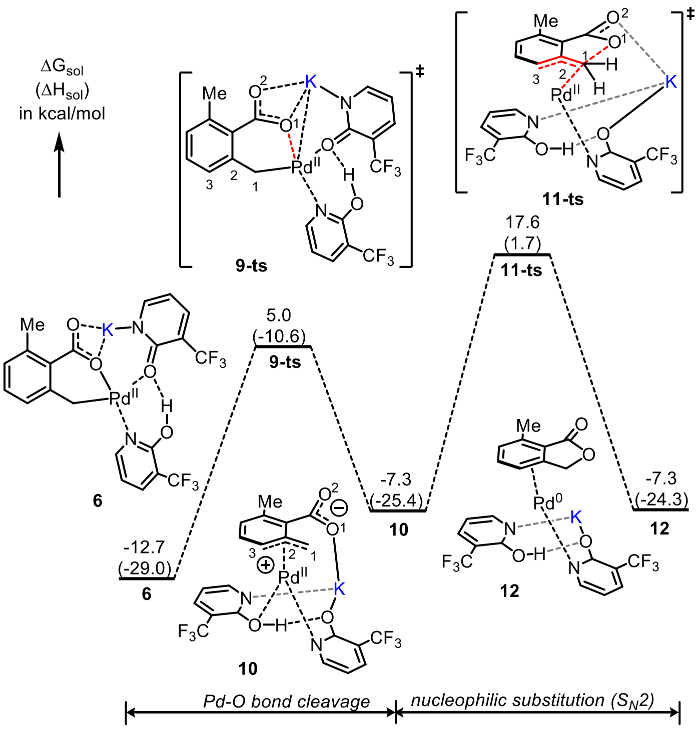

Supplementary Figure 14. Energy profile for the stepwise C–O formation process: Pd–O bond cleavage and nucleophilic substitution [The energies were obtained with 6-31G(d,p)/Lanl2dz for CHONFK/Pd basis sets (BS1)].

(14) Control experiment with different amounts of water.

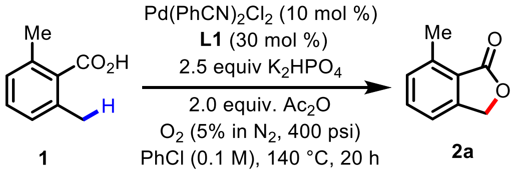

Reaction condition	Yield
standard	70%
2.0 equiv. H_2O	62%
10.0 equiv. H_2O	10%

Supplementary Figure 15. Control experiment with different amounts of water.

(15) Results with the explicit PhCl solvent molecule

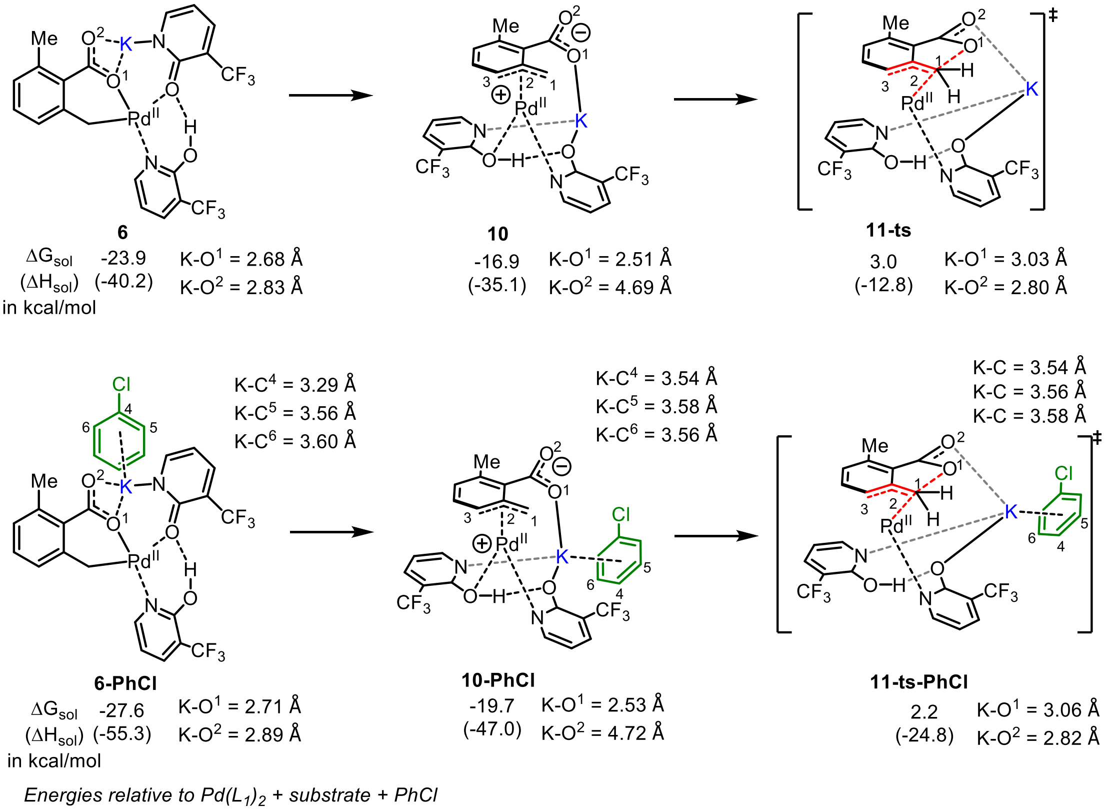

Supplementary Figure 16. The effects of the coordination of PhCl to K cation in the rate-determining step.

We have performed calculations with explicit PhCl solvent surrounding K^+ in the rate-determining step (specially on intermediates **6**, **10**, and transition state **11-ts**). Our results (**Figure 16**) show that the coordination of PhCl to K cation is relatively weak, with the K-C (carbon atoms from PhCl) bond distances varying from 3.29 to 3.60 Å, and the K-O (oxygen atoms from substrate) bond distances elongating only slightly (by 0.02-0.06 Å).

Although the coordination stabilizes these species slightly, lowering down the free energies by 3.7, 2,8, and 0.8 kcal/mol to **6**, **10** and **11-ts**, respectively, this increases the overall activation free energy barrier by 2.9 kcal/mol (**11-ts-PhCl** relative to **6-PhCl**). Thus, our calculation indicates that the weak cation-pi interaction would stabilize those species but increase the overall activation barrier which is unfavorable.

(16) **Supplementary Table 1**. Energetics for all calculated species

Species	Electronic Energy (EE)	EE + ZPE	Enthalpy	Free Energy	High-Level Single Point Energies	Imaginary Frequency (cm^{-1}*i)
**sub**	-499.488112	-499.317100	-499.305823	-499.352847	-499.625468	
**PhCN**	-324.511778	-324.412344	-324.405317	-324.442606	-324.598123	
**PdPhCN2Cl2**	-1696.281741	-1696.077506	-1696.056919	-1696.134303	-1697.705442	
**PdP2**	-2612.676663	-2612.619799	-2612.602104	-2612.668360	-2614.212049	
**O2_t**	-150.318846	-150.315072	-150.311765	-150.335050	-150.372589	
**O2_s**	-150.256485	-150.252747	-150.249440	-150.271690	-150.311362	
**L1**	-660.569992	-660.471737	-660.462078	-660.506216	-660.779146	
**KHPO4_anion**	-1243.011883	-1242.985268	-1242.977483	-1243.017154	-1243.223086	
**KH2PO4**	-1243.537212	-1243.499468	-1243.490778	-1243.533262	-1243.725183	
**KCl_tetramer**	-4240.937097	-4240.932871	-4240.915952	-4240.981957	-4241.236980	
**KCl**	-1060.195841	-1060.195386	-1060.191521	-1060.219036	-1060.276669	
**K2HPO4**	-1842.900966	-1842.873266	-1842.863446	-1842.909566	-1843.137243	
**h2o2**	-151.545416	-151.519801	-151.515961	-151.541301	-151.607988	
**1**	-1098.856740	-1098.697858	-1098.684602	-1098.738022	-1099.040383	
**1b_K**	-1059.536350	-1059.404873	-1059.393397	-1059.443286	-1059.711546	
**1p**	-3112.164887	-3111.935320	-3111.906970	-3111.998159	-3113.839144	
**2**	-1446.723079	-1446.547239	-1446.526427	-1446.600423	-1448.316172	
**2-tsp**	-3112.148208	-3111.923498	-3111.895862	-3111.984126	-3113.821885	-1239.41
**3**	-2545.628994	-2545.292822	-2545.258424	-2545.361086	-2547.386599	
**3b**	-2506.302982	-2505.994605	-2505.961801	-2506.062155	-2508.053731	
**3b’**	-2506.282371	-2505.974902	-2505.942331	-2506.042519	-2508.038281	
**3h**	-1946.245525	-1945.897121	-1945.864453	-1945.964144	-1947.968909	
**3p**	-3112.211249	-3111.981661	-3111.953284	-3112.044705	-3113.883133	
**4-ts**	-2545.592451	-2545.260857	-2545.227795	-2545.327527	-2547.354203	-1135.19
**4b-ts-bz**	-2506.273809	-2505.969987	-2505.938381	-2506.035949	-2508.028124	-1112.44
**4-tsh**	-1946.213774	-1945.869785	-1945.838496	-1945.934563	-1947.941217	-927.00
**4b-ts-o**	-2506.277956	-2505.975067	-2505.942837	-2506.041166	-2508.032360	-1097.17
**5**	-2545.637965	-2545.301360	-2545.267340	-2545.370300	-2547.400375	
**5b-bz**	-2506.316547	-2506.007410	-2505.975074	-2506.074783	-2508.070248	
**5h**	-1946.257361	-1945.908057	-1945.876065	-1945.974500	-1947.982355	
**5b-o**	-2506.317542	-2506.009211	-2505.976273	-2506.077391	-2508.071956	
**6**	-2545.645261	-2545.308517	-2545.275126	-2545.376477	-2547.404150	

**6b-bz**	-2506.327285	-2506.018220	-2505.986340	-2506.084846	-2508.078989	
**6-Cs**	-1965.635398	-1965.299530	-1965.265698	-1965.369620	-1967.634241	
**6-Li**	-1953.285758	-1952.946760	-1952.914410	-1953.012119	-1955.000010	
**6-Na**	-2108.041930	-2107.704409	-2107.671327	-2107.771360	-2109.763700	
**6b-o**	-2506.317819	-2506.009355	-2505.976858	-2506.076441	-2508.069772	
**6p**	-3711.610944	-3711.392462	-3711.362081	-3711.455697	-3713.322115	
**7-ts**	-2545.568476	-2545.235542	-2545.201289	-2545.306816	-2547.322882	-551.93
**7-ts-a**	-2695.907563	-2695.568215	-2695.531177	-2695.641960	-2697.694048	-507.97
**7-ts-b**	-2695.895415	-2695.554890	-2695.518440	-2695.626748	-2697.708797	-423.04
**7-ts-c**	-2697.127767	-2696.765375	-2696.726994	-2696.840771	-2698.939647	-551.46
**7-ts-c-rea**	-2697.212317	-2696.846747	-2696.808729	-2696.919729	-2699.027650	
**7-ts-d**	-2035.293604	-2035.054587	-2035.026976	-2035.118338	-2036.876068	-471.64
**7-ts-e**	-2035.293945	-2035.053317	-2035.026758	-2035.112530	-2036.900117	-411.67
**7-ts-f**	-1884.963466	-1884.731379	-1884.707515	-1884.787906	-1886.519953	-386.01
**8**	-2545.617860	-2545.281715	-2545.247313	-2545.352245	-2547.371227	
**9-ts**	-2545.610208	-2545.275004	-2545.241431	-2545.343971	-2547.373509	-75.83
**9b-ts-bz**	-2506.285790	-2505.978205	-2505.946319	-2506.045304	-2508.041890	-88.77
**9b-ts-o**	-2506.282630	-2505.975909	-2505.943341	-2506.042704	-2508.033377	-45.03
**10**	-2545.634322	-2545.300159	-2545.266914	-2545.365351	-2547.395725	
**10b-bz**	-2506.310150	-2506.003494	-2505.971026	-2506.069758	-2508.063608	
**10b-o**	-2506.318531	-2506.011157	-2505.978521	-2506.076161	-2508.066856	
**11b-ts-o**	-2506.245402	-2505.940824	-2505.907700	-2506.008435	-2507.991991	-228.75
**11-ts**	-2545.599712	-2545.265456	-2545.231484	-2545.333613	-2547.353272	-464.64
**11b-ts-bz**	-2506.275796	-2505.969398	-2505.937207	-2506.034369	-2508.021712	-471.59
**11-ts-Cs**	-1965.584784	-1965.251598	-1965.217162	-1965.322020	-1967.580933	-449.88
**11-ts-Cs-rea**	-1965.617782	-1965.283845	-1965.249370	-1965.353144	-1967.618536	
**11-ts-H**	-1946.189461	-1945.842473	-1945.810259	-1945.908323	-1947.911464	-423.79
**11-ts-H-rea**	-1946.242080	-1945.894787	-1945.862786	-1945.959905	-1947.966806	
**11-ts-Li**	-1953.239323	-1952.902308	-1952.869761	-1952.967320	-1954.946574	-491.25
**11-ts-Li-rea**	-1953.278493	-1952.941170	-1952.908182	-1953.006285	-1954.991922	
**11-ts-Na**	-2107.995815	-2107.660657	-2107.627251	-2107.727043	-2109.710947	-473.70
**11-ts-Na-rea**	-2108.033744	-2107.698516	-2107.664783	-2107.764833	-2109.755015	
**11-tsp**	-3711.560672	-3711.344617	-3711.314056	-3711.407228	-3713.268039	-470.35
**12**	-2545.647333	-2545.310617	-2545.276907	-2545.377158	-2547.396961	
**12b-bz**	-2506.323230	-2506.014551	-2505.982389	-2506.079983	-2508.064929	
**12b-o**	-2506.265917	-2505.959983	-2505.926692	-2506.027894	-2508.009646	
**PdL_1P**	-2029.702287	-2029.585837	-2029.566734	-2029.635930		
**3-LP-a**	-3128.613700	-3128.336646	-3128.303602	-3128.405399		
**3-LP-b**	-3128.617947	-3128.340907	-3128.308388	-3128.406502		

**4-ts-LP-a**	-3128.577152	-3128.303551	-3128.272301	-3128.369149		-1197.05
**4-ts-LP-b**	-3128.555074	-3128.283564	-3128.251706	-3128.349489		-1175.73
**5-LP**	-3128.608922	-3128.330030	-3128.298187	-3128.396671		
**6-LP**	-3128.623207	-3128.346717	-3128.315071	-3128.413593		
**11-ts-LP**	-3128.569271	-3128.293429	-3128.261147	-3128.361424		-451.93
**3-1L**	-1885.465094	-1885.216539	-1885.191639	-1885.273871		
**4-ts-1L**	-1885.410591	-1885.165981	-1885.142310	-1885.221561		-126.72
**6-3L**	-3205.764728	-3205.341624	-3205.298167	-3205.421255		
**11-ts-3L**	-3205.707911	-3205.287350	-3205.243435	-3205.368201		-519.12
**7-ts-b-LP**	-3278.917315	-3278.635502	-3278.601311	-3278.702416		-394.91
**7-ts-b-1L**	-2035.725859	-2035.471822	-2035.445110	-2035.530055		-424.59
**7-ts-b-3L**	-3356.010060	-3355.583806	-3355.537238	-3355.667187		-421.42
**PhCl**	-691.862031	-691.770897	-691.764454	-691.800684		
**6-PhCl**	-3237.533429	-3237.104470	-3237.063576	-3237.183169		
**10-PhCl**	-3237.518247	-3237.091937	-3237.050424	-3237.170461		
**11-ts-PhCl**	-3237.482792	-3237.056562	-3237.015068	-3237.135595		-465.47

(17) **Supplementary Table 2.** Cartesian coordinates for all calculated species

sub

C	2.007512	-1.106111	-0.096946
C	0.612530	-1.211480	-0.061712
C	-0.150618	-0.022307	0.012654
C	0.472808	1.245512	0.078654
C	1.872067	1.298223	0.063782
C	2.634845	0.136495	-0.031816
H	2.604174	-2.011113	-0.170844
H	2.364069	2.264791	0.129367
H	3.719430	0.199251	-0.050741
C	-1.639446	-0.145525	0.061273
O	-2.254430	-0.948925	0.737599
O	-2.273324	0.727173	-0.754969
H	-3.228907	0.583543	-0.628052
C	-0.304856	2.537989	0.191249
H	-0.765977	2.807905	-0.763684
H	-1.114216	2.466931	0.924163
H	0.356703	3.354330	0.493572
C	-0.025927	-2.580087	-0.118084
H	-0.483519	-2.839169	0.840906
H	-0.823609	-2.628457	-0.866222
H	0.720395	-3.340251	-0.363824

PhCN

C	-1.482575	-1.212718	-0.000002
C	-0.089698	-1.221077	0.000018
C	0.608040	-0.000043	-0.000001
C	-0.089660	1.221062	0.000007
C	-1.482504	1.212760	0.000007
C	-2.178110	0.000021	-0.000014
H	-2.025413	-2.152754	-0.000013
H	0.459618	-2.156623	0.000012
H	0.459747	2.156554	0.000004
H	-2.025349	2.152793	0.000001
H	-3.264122	0.000072	-0.000015
C	2.041323	-0.000026	-0.000008
N	3.204946	0.000011	-0.000004

PdPhCN2Cl2

Pd	0.000000	-0.000004	0.000179
Cl	-0.000061	2.379419	0.000162
Cl	0.000058	-2.379465	0.000213
C	-6.662737	-1.214940	-0.004229
C	-5.271302	-1.225976	-0.002642
C	-4.579959	-0.000029	0.001291
C	-5.271246	1.225958	0.003676
C	-6.662680	1.214993	0.002050
C	-7.355685	0.000044	-0.001893
H	-7.207027	-2.153755	-0.007285
H	-4.718517	-2.159292	-0.004430
H	-4.718419	2.159246	0.006629
H	-7.206929	2.153835	0.003811
H	-8.441602	0.000072	-0.003179
C	-3.155993	-0.000060	0.002456
N	-1.998391	-0.000062	0.003189
C	6.662745	1.214959	-0.002533

C	5.271311	1.225998	-0.003907
C	4.579960	0.000050	-0.001449
C	5.271239	-1.225935	0.002323
C	6.662674	-1.214975	0.003650
C	7.355687	-0.000027	0.001239
H	7.207043	2.153772	-0.004360
H	4.718534	2.159317	-0.006732
H	4.718403	-2.159222	0.004169
H	7.206915	-2.153819	0.006569
H	8.441604	-0.000057	0.002330
C	3.155993	0.000089	-0.002382
N	1.998391	0.000058	-0.002907

PdP2

Pd	0.010246	0.026102	0.016134
P	2.492882	1.065932	-0.099331
O	1.221367	1.416462	-0.958285
O	1.899108	-0.040200	0.887557
O	3.744646	0.598322	-0.796135
O	2.880174	2.386435	0.778725
H	2.076024	2.771258	1.159761
K	3.634325	-1.922706	0.013970
P	-2.443119	-1.071565	0.092813
O	-1.195157	-1.362159	0.997541
O	-3.750294	-0.670694	0.741687
O	-1.878482	0.071028	-0.863876
O	-2.679257	-2.416043	-0.806896
H	-3.627726	-2.608856	-0.839001
K	-3.718688	1.862507	-0.056481

O2_t

O	0.000000	0.000000	0.607099
O	0.000000	0.000000	-0.607099

O2_s

O	0.000000	0.000000	0.607783
O	0.000000	0.000000	-0.607783

L1

C	-0.785864	0.930775	0.000062
C	-0.032489	-0.260569	0.000074
C	-0.727144	-1.465272	-0.000063
C	-2.123873	-1.453489	-0.000057
C	-2.766917	-0.219710	-0.000003
N	-2.114754	0.954037	0.000123
H	-0.177316	-2.399707	-0.000002
H	-2.693905	-2.375343	-0.000181
H	-3.852810	-0.159373	0.000190
O	-0.135514	2.109143	-0.000154
H	-0.816008	2.804753	0.000168
C	1.466677	-0.212731	0.000000
F	1.955170	0.430871	-1.085732
F	2.005666	-1.454365	-0.000111
F	1.955285	0.430633	1.085856

KHPO4_anion

P	0.876885	-0.145175	-0.041568
O	2.395089	-0.172187	-0.054137
O	0.166245	-0.066381	-1.420159
O	0.137645	-1.094651	0.929179
O	0.396281	1.393648	0.648859
H	0.665830	2.048794	-0.009844
K	-2.030589	-0.018663	-0.010346

KH2PO4

P	-0.936620	-0.033053	0.160627
O	-2.420241	0.122188	0.238995
O	-0.032076	-0.238785	1.354285
O	-0.458752	-1.221220	-0.897641
H	-0.971575	-1.159494	-1.717430
O	-0.287062	1.246469	-0.722766
H	-0.811479	2.041591	-0.549631
K	2.179863	0.018131	0.003931

KCl_tetramer

K	2.518529	0.786134	-0.499717
Cl	0.186927	2.722557	0.306960
K	-1.672826	1.211215	-1.713147
Cl	0.676452	-0.680097	-2.570809
K	-0.658844	0.663145	2.513976
Cl	1.712429	-1.241118	1.749923
K	-0.185877	-2.660951	-0.300491
Cl	-2.576905	-0.800831	0.513232

KCl

K	0.000000	0.000000	1.356964
Cl	0.000000	0.000000	-1.516606

K2HPO4

P	0.014088	0.675842	-0.135466
O	1.148111	0.982745	-1.124773
O	-1.350050	1.332210	-0.384087
O	-0.114223	-0.848899	0.175169
O	0.613107	1.243188	1.355757
H	0.932294	2.143319	1.197936
K	-2.682147	-0.824477	0.030864
K	2.496927	-0.962623	0.003743

h2o2

O	-0.000000	0.733666	-0.000263
H	0.960729	0.878171	0.002100
O	0.000000	-0.733666	-0.000263
H	-0.960729	-0.878171	0.002100

1

C	-2.812338	1.202974	-0.079066
C	-1.411368	1.222481	-0.070538
C	-0.710583	0.000892	0.000575
C	-1.409665	-1.221660	0.071955
C	-2.810658	-1.204055	0.080938
C	-3.511868	-0.001020	0.001052
H	-3.356303	2.142513	-0.145004
H	-3.353319	-2.144333	0.147064
H	-4.598874	-0.001767	0.001243
C	0.811645	0.001949	0.000261
O	1.390728	0.634164	0.933030

O	1.391103	-0.629558	-0.932830
C	-0.668774	-2.537118	0.150110
H	-0.037276	-2.677149	-0.731315
H	-0.000894	-2.565436	1.020126
H	-1.363786	-3.378169	0.231968
C	-0.672259	2.538924	-0.148997
H	-0.040488	2.679758	0.732104
H	-0.004887	2.568168	-1.019375
H	-1.368415	3.379068	-0.230442
K	3.741860	0.002859	0.000232

1b_K

C	2.718439	-1.633414	-0.073258
C	1.334141	-1.478092	-0.047331
C	0.731957	-0.211097	-0.000294
C	1.551777	0.942629	0.038782
C	2.945100	0.765872	0.025964
C	3.530879	-0.498980	-0.035435
H	3.157914	-2.626479	-0.117761
H	3.583419	1.645739	0.065443
H	4.613593	-0.594916	-0.048972
C	-0.792075	-0.163956	0.000655
O	-1.399489	-1.246644	0.263255
O	-1.358218	0.937851	-0.271146
C	1.006447	2.350527	0.107800
H	0.471569	2.612172	-0.809124
H	0.277510	2.458186	0.915222
H	1.819280	3.068089	0.262609
K	-3.721022	-0.115348	-0.012915
H	0.677805	-2.341770	-0.060197

1p

Pd	0.159150	0.069752	-0.186207
C	-3.827067	3.935180	0.701435
C	-3.851495	2.538826	0.605523
C	-2.833181	1.891690	-0.129967
C	-1.827492	2.644825	-0.767044
C	-1.844929	4.041370	-0.666541
C	-2.837501	4.685641	0.067336
H	-4.598623	4.437567	1.278813
H	-1.073168	4.619631	-1.167872
H	-2.840846	5.769119	0.145760
C	-2.882419	0.383324	-0.236198
O	-3.890108	-0.187558	-0.642099
O	-1.834188	-0.297558	0.185210
H	-1.622099	-1.917970	-0.365610
C	-0.712341	1.996258	-1.550150
H	-1.014641	1.097823	-2.097880
H	-0.272779	2.671051	-2.291533
H	0.189823	1.846394	-0.883923
C	-4.947554	1.759966	1.294854
H	-5.599442	1.274916	0.563248
H	-4.538461	0.963425	1.926284
H	-5.552540	2.417140	1.925434
P	0.341188	-2.886758	-0.091892
O	0.546110	-1.664996	0.874448
O	0.527590	-4.254387	0.496417
O	-1.208726	-2.796120	-0.604978
O	1.279916	-2.639720	-1.371748
H	1.721814	-1.748587	-1.313461
K	-1.852276	-5.303420	0.360644
P	3.152016	0.363528	0.196774
O	2.025701	-0.052838	-0.903498
O	4.486986	0.581492	-0.490273
O	2.624549	1.551318	1.002335
O	3.264238	-0.974011	1.133162
H	2.362900	-1.313161	1.302254
K	4.690510	3.047369	0.409013

2

Pd	-0.000009	-0.000171	0.019053
C	2.489240	-0.179650	0.006873
C	3.883585	-0.399522	-0.009972
C	4.330355	-1.715708	-0.017886
C	3.422075	-2.784618	-0.010930
C	2.062162	-2.504787	0.003926
N	1.635729	-1.234723	0.012784
H	5.397596	-1.909496	-0.030582
H	3.767416	-3.811125	-0.017380
H	1.301867	-3.278611	0.009057
O	1.850397	0.958314	0.015791
C	4.815254	0.772390	-0.013413
F	4.597936	1.587388	-1.071854
F	6.109555	0.384243	-0.066804
F	4.672865	1.533752	1.097118
C	-2.489238	0.179580	0.007821
C	-3.883557	0.399654	-0.009734
C	-4.330158	1.715884	-0.018102
C	-3.421701	2.784659	-0.011104
C	-2.061850	2.504643	0.004203
N	-1.635537	1.234519	0.013707
H	-5.397362	1.909845	-0.031307
H	-3.766861	3.811226	-0.017922
H	-1.301470	3.278387	0.009291
O	-1.850681	-0.958517	0.017128
C	-4.815377	-0.772128	-0.013570
F	-4.597352	-1.587445	-1.071611
F	-6.109568	-0.383803	-0.068049
F	-4.673945	-1.533210	1.097305

2-tsp

Pd	-0.104582	-0.214067	-0.184665
C	2.815644	4.388621	0.704143
C	1.454053	4.180129	0.450284
C	1.053261	2.972520	-0.166660
C	2.016663	2.008496	-0.541492
C	3.371498	2.261878	-0.283255
C	3.770760	3.441474	0.337550
H	3.126125	5.306574	1.196004
H	4.112361	1.523024	-0.578492
H	4.823354	3.623737	0.536818
C	-0.423059	2.755845	-0.419806
O	-1.117715	3.628853	-0.931705
O	-0.954705	1.621173	0.000909
H	-2.487561	0.872128	-0.530430
C	1.645431	0.713066	-1.200508

H	0.879296	0.826016	-1.979617
H	2.487229	0.271256	-1.749180
H	1.862426	-0.283389	-0.351827
C	0.458663	5.240468	0.862092
H	0.013830	5.720774	-0.013489
H	-0.371040	4.814987	1.436199
H	0.943377	6.005176	1.475649
P	-2.892237	-1.233001	0.029045
O	-1.669782	-0.991908	0.962952
O	-4.183154	-1.690981	0.643659
O	-3.211478	0.210598	-0.689551
O	-2.407391	-2.231640	-1.142208
H	-1.440445	-2.412643	-1.055160
K	-5.806519	0.294678	0.247211
P	1.491628	-2.579872	0.327417
O	0.348614	-2.155611	-0.695877
O	2.217332	-3.863594	0.001339
O	2.473950	-1.347523	0.397000
O	0.709900	-2.735140	1.754875
H	-0.155822	-2.287794	1.704509
K	4.617295	-2.835124	-0.131871

3

Pd	0.221114	-0.044533	-0.315897
C	3.161288	0.314824	0.030412
C	3.568175	1.685130	0.021808
C	4.784156	2.046626	-0.546369
C	5.602304	1.066343	-1.107600
C	5.145529	-0.245797	-1.066063
N	3.975929	-0.619240	-0.525365
H	5.089574	3.087405	-0.546011
H	6.556972	1.311925	-1.558485
H	5.750356	-1.045836	-1.491721
O	2.065893	-0.094038	0.622634
C	2.681621	2.747568	0.591848
F	1.604234	2.986055	-0.203137
F	3.323558	3.934044	0.722766
F	2.198194	2.436120	1.819318
C	-1.928698	1.237066	-0.393106
C	-3.172414	1.894552	-0.256010
C	-3.261516	2.932801	0.662048
C	-2.146350	3.300402	1.428104
C	-0.951914	2.614119	1.246592
N	-0.855677	1.629993	0.342064
H	-4.206119	3.451572	0.787574
H	-2.205665	4.105556	2.150622
H	-0.056810	2.847982	1.807980
O	-1.662746	0.218599	-1.157193
C	-4.333340	1.436267	-1.079960
F	-4.082542	1.529889	-2.407427
F	-5.446196	2.165791	-0.833419
F	-4.652938	0.138504	-0.842984
C	-1.818132	-2.225977	2.790198
C	-0.775550	-2.533400	1.909909
C	-1.068757	-2.640355	0.534533
C	-2.376574	-2.479747	0.044661
C	-3.394856	-2.175967	0.957379
C	-3.119130	-2.042680	2.317260
H	-1.605646	-2.129699	3.851901
H	-4.405590	-2.025299	0.590450
H	-3.918757	-1.799037	3.011685
C	0.064849	-2.934615	-0.418556
O	0.469616	-4.096299	-0.577529
O	0.646039	-1.931748	-1.026048
C	-2.681614	-2.641690	-1.424204
H	-1.881122	-2.230669	-2.043576
H	-2.799320	-3.701784	-1.683202
H	-3.604722	-2.121261	-1.688777
C	0.636768	-2.719065	2.416224
H	1.028841	-3.700641	2.124572
H	1.305244	-1.947254	2.014397
H	0.675455	-2.655615	3.507186
K	3.017388	-3.194415	-0.640318

3b

Pd	-0.132261	0.085276	-0.406766
C	-3.046465	-0.454961	0.031942
C	-3.369307	-1.844402	0.126461
C	-4.577400	-2.315268	-0.373351
C	-5.471100	-1.425244	-0.968750
C	-5.093412	-0.089106	-1.031287
N	-3.932482	0.389347	-0.558062
H	-4.817864	-3.369904	-0.293929
H	-6.422857	-1.756322	-1.368126
H	-5.759752	0.641859	-1.488173
O	-1.962827	0.054487	0.563168
C	-2.399255	-2.809792	0.731142
F	-1.324613	-3.014471	-0.076756
F	-2.951950	-4.029529	0.940949
F	-1.913932	-2.398835	1.928348
C	2.100549	-1.046837	-0.463495
C	3.388359	-1.609428	-0.309507
C	3.555352	-2.602015	0.647407
C	2.474147	-3.015322	1.438135
C	1.234594	-2.419185	1.242711
N	1.063443	-1.483255	0.298888
H	4.535082	-3.047592	0.784394
H	2.594388	-3.784359	2.191741
H	0.363060	-2.687281	1.825023
O	1.757268	-0.081737	-1.265773
C	4.514011	-1.097099	-1.150434
F	4.269135	-1.245287	-2.474014
F	5.674455	-1.741927	-0.886754
F	4.743639	0.224661	-0.950478
C	1.655252	2.566001	2.693352
C	0.605782	2.760813	1.788422
C	0.910986	2.776862	0.410981
C	2.228518	2.619931	-0.031361
C	3.261338	2.433239	0.888548
C	2.971755	2.402507	2.253238
H	1.435178	2.541060	3.757796
H	4.277972	2.290583	0.537343
H	3.767767	2.251445	2.977274
C	-0.207113	2.962902	-0.584747
O	-0.694739	4.087608	-0.775882
O	-0.685856	1.907805	-1.191037

C	-0.820000	2.904875	2.266641
H	-1.268175	3.829137	1.884506
H	-1.431211	2.059211	1.925576
H	-0.869819	2.933298	3.358612
K	-3.155522	3.007266	-0.899093
H	2.437494	2.607816	-1.095586

3b'

Pd	-0.486454	0.201605	0.070225
C	-0.791214	-2.729495	0.250412
C	0.220242	-3.306060	-0.574243
C	-0.047866	-4.492611	-1.249314
C	-1.291526	-5.107899	-1.109616
C	-2.220367	-4.494442	-0.277537
N	-1.989072	-3.345892	0.372588
H	0.718027	-4.937124	-1.874859
H	-1.527010	-6.032536	-1.624068
H	-3.203163	-4.937152	-0.126483
O	-0.614229	-1.640618	0.981466
C	1.576158	-2.675694	-0.717691
F	1.532159	-1.507162	-1.407414
F	2.434912	-3.477863	-1.392847
F	2.151944	-2.412254	0.475175
C	2.335757	1.056905	0.071982
C	3.651519	1.152611	0.677345
C	3.901895	0.671990	1.943164
C	2.874405	0.061586	2.685324
C	1.632365	-0.049812	2.093744
N	1.368637	0.426752	0.861174
H	4.898562	0.762705	2.363268
H	3.044064	-0.328098	3.681576
H	0.810019	-0.552725	2.591316
O	2.048136	1.496843	-1.055203
C	4.726473	1.796561	-0.137454
F	4.951966	1.154695	-1.309778
F	5.915705	1.820303	0.521438
F	4.441488	3.084817	-0.454797
C	-4.252190	4.435895	0.143384
C	-4.310438	3.045761	0.095409
C	-3.243715	2.282145	-0.407553
C	-2.092975	2.944306	-0.890999
C	-2.052682	4.344763	-0.842290
C	-3.113442	5.090040	-0.328265
H	-5.088436	5.002157	0.542703
H	-1.170616	4.855595	-1.219160
H	-3.049439	6.174059	-0.301015
C	-3.439501	0.782970	-0.384507
O	-4.581749	0.333730	-0.170484
O	-2.442839	-0.043122	-0.553155
C	-0.899099	2.229131	-1.461780
H	-1.153466	1.397178	-2.118889
H	-0.243716	2.904126	-2.018291
H	-0.154739	1.930365	-0.663594
K	-3.635802	-1.524618	1.551043
H	-5.190832	2.519926	0.446912

3h

Pd	0.098831	-0.095373	-0.852947
C	-2.951518	-0.253458	-0.646065
C	-4.149186	-0.832381	-0.121525
C	-5.374405	-0.267887	-0.403361
C	-5.467824	0.890308	-1.203363
C	-4.306608	1.448996	-1.673214
N	-3.106179	0.879220	-1.397195
H	-6.272398	-0.717301	0.006520
H	-6.424411	1.343889	-1.427986
H	-4.268816	2.353873	-2.267835
O	-1.807927	-0.761900	-0.415199
C	-4.004310	-2.027345	0.776110
F	-3.397921	-3.064638	0.157927
F	-5.204186	-2.468463	1.212902
F	-3.263507	-1.736260	1.872808
C	2.366653	-1.065632	-0.576433
C	3.601967	-1.628092	-0.191122
C	3.585300	-2.888970	0.392287
C	2.374056	-3.565029	0.607065
C	1.187082	-2.945491	0.237873
N	1.209573	-1.742348	-0.350766
H	4.522859	-3.344017	0.694000
H	2.356801	-4.547802	1.062193
H	0.211965	-3.393488	0.398576
O	2.153969	0.100748	-1.117157
C	4.855172	-0.834382	-0.389480
F	5.077156	-0.552777	-1.694568
F	5.944171	-1.493020	0.067726
F	4.806582	0.355480	0.258519
C	0.507773	1.686830	3.030028
C	-0.308245	1.973481	1.927152
C	0.302998	2.438994	0.746450
C	1.697866	2.631491	0.665816
C	2.478040	2.328621	1.785773
C	1.889263	1.856837	2.960307
H	0.049357	1.322127	3.946020
H	3.555865	2.453252	1.729684
H	2.509147	1.623126	3.821725
C	-0.552743	2.712979	-0.469669
O	-1.198240	3.741746	-0.608612
O	-0.587364	1.751877	-1.388450
H	-2.222311	1.352815	-1.673628
C	2.334376	3.155180	-0.598421
H	1.921876	2.665054	-1.483358
H	2.163099	4.233927	-0.703789
H	3.412993	2.979415	-0.593214
C	-1.795573	1.712869	2.010337
H	-2.357274	2.289031	1.271040
H	-2.012864	0.650669	1.848646
H	-2.180807	1.974284	3.000950

3p

Pd	0.039925	0.087951	-0.249984
C	-0.002723	5.385892	0.621318
C	-1.097055	4.518188	0.485760
C	-0.895991	3.272560	-0.143914
C	0.374287	2.924656	-0.667216
C	1.443204	3.820602	-0.511001
C	1.257120	5.042115	0.132516

H	-0.144582	6.338227	1.125613
H	2.417994	3.556735	-0.913755
H	2.091233	5.729017	0.249740
C	-2.034590	2.279341	-0.217199
O	-3.176945	2.622871	-0.515722
O	-1.743112	1.039522	0.121989
H	-2.555952	-0.410826	-0.234012
C	0.580138	1.647487	-1.405888
H	-0.093821	1.542474	-2.265523
H	1.614402	1.500410	-1.727455
H	2.435153	0.218801	1.152520
C	-2.440155	4.931080	1.042069
H	-3.168761	5.072031	0.239487
H	-2.857295	4.162322	1.700726
H	-2.353164	5.862286	1.609441
P	-1.690737	-2.461837	0.015233
O	-0.706051	-1.760894	0.982250
O	-2.451098	-3.678916	0.465482
O	-2.833169	-1.357766	-0.390410
O	-0.872246	-2.794282	-1.348100
H	-0.005509	-2.334889	-1.357248
K	-4.922613	-2.910189	0.347797
P	2.560094	-1.729606	0.104425
O	1.565079	-1.201626	-0.964003
O	3.795538	-2.438819	-0.369199
O	3.145101	-0.398128	0.908243
O	1.755791	-2.571420	1.197016
H	0.772573	-2.343999	1.187382
K	5.766524	-0.915209	0.295279

4-ts

Pd	0.361417	0.121164	0.167630
C	1.514893	-2.570016	-0.388540
C	0.700838	-3.504876	0.327532
C	1.284034	-4.641896	0.877383
C	2.651887	-4.868507	0.728013
C	3.378371	-3.924948	0.012563
N	2.843149	-2.817401	-0.519277
H	0.666304	-5.353236	1.414228
H	3.131328	-5.746195	1.146044
H	4.448683	-4.055395	-0.140094
O	1.051936	-1.493851	-0.985003
C	-0.780274	-3.313298	0.471176
F	-1.103070	-2.262322	1.272235
F	-1.380288	-4.396336	1.023421
F	-1.391339	-3.103389	-0.716978
C	-2.561135	0.470131	-0.025456
C	-3.849990	0.388785	-0.640870
C	-3.990304	-0.168561	-1.898137
C	-2.866520	-0.663666	-2.574042
C	-1.640979	-0.591530	-1.938432
N	-1.493544	-0.052022	-0.712178
H	-4.973074	-0.217084	-2.354879
H	-2.945161	-1.105559	-3.559783
H	-0.736600	-0.992998	-2.379961
O	-2.364674	1.011258	1.110394
C	-5.021126	0.941977	0.109852
F	-5.201948	0.336858	1.307540
F	-6.177676	0.789344	-0.582552
F	-4.892750	2.267427	0.362315
C	1.885878	5.075660	-0.422792
C	2.681292	3.942232	-0.199614
C	2.114307	2.837358	0.486216
C	0.778327	2.907867	0.955827
C	0.024067	4.064753	0.721040
C	0.570282	5.140754	0.028841
H	2.311518	5.918300	-0.960633
H	-0.998037	4.112002	1.087050
H	-0.025689	6.030643	-0.154026
C	2.921023	1.575282	0.670962
O	4.164306	1.606688	0.670290
O	2.313513	0.417478	0.766137
C	0.119965	1.787523	1.707060
H	0.784091	1.220642	2.358757
H	-0.648275	2.208398	2.368798
H	-0.969491	1.208113	1.236056
C	4.097519	3.968937	-0.729186
H	4.827914	3.927709	0.081887
H	4.311164	3.111116	-1.374478
H	4.263726	4.881608	-1.308920
K	4.021475	-0.461907	-1.195559

4b-ts-bz

Pd	-0.339901	0.323082	-0.133647
C	-2.423679	-1.739459	0.393277
C	-2.077220	-2.823959	-0.473209
C	-3.087025	-3.606872	-1.024632
C	-4.422450	-3.338081	-0.726931
C	-4.682569	-2.279467	0.134774
N	-3.730529	-1.503704	0.669810
H	-2.826724	-4.431790	-1.678470
H	-5.227566	-3.932751	-1.142967
H	-5.706745	-2.030987	0.408268
O	-1.548003	-0.964097	0.997196
C	-0.649361	-3.166938	-0.783673
F	-0.046886	-2.248289	-1.588730
F	-0.543409	-4.351180	-1.433672
F	0.112377	-3.270576	0.328335
C	2.537867	-0.328965	0.082648
C	3.711045	-0.877110	0.692362
C	3.621864	-1.566295	1.886687
C	2.374915	-1.735127	2.504765
C	1.260577	-1.211249	1.877337
N	1.334761	-0.538692	0.710477
H	4.520454	-1.971534	2.339533
H	2.272377	-2.269919	3.441092
H	0.260918	-1.334575	2.276459
O	2.569164	0.345083	-0.996321
C	5.022704	-0.665471	0.001753
F	5.045008	-1.200653	-1.241885
F	6.046924	-1.231592	0.687922
F	5.327775	0.647487	-0.136988
C	-0.123858	5.644865	0.290232
C	-1.194855	4.762735	0.164680
C	-1.033881	3.504987	-0.434250
C	0.226692	3.132811	-0.954610

C	1.294606	4.033192	-0.820064
C	1.130005	5.270747	-0.198346
H	-0.264798	6.610954	0.765844
H	2.267313	3.755483	-1.217881
H	1.977780	5.943919	-0.105763
C	-2.235620	2.595068	-0.442439
O	-3.362073	3.062586	-0.191671
O	-2.089967	1.311728	-0.646236
C	0.461897	1.829359	-1.656182
H	-0.370966	1.502557	-2.279496
H	1.308762	1.938102	-2.346370
H	1.306212	0.961373	-1.146640
K	-3.749037	1.047783	1.616403
H	-2.178484	5.028498	0.536829

4-tsh

Pd	-0.020479	0.300801	-0.551191
C	-2.990438	-0.354421	0.030238
C	-4.053209	-1.271106	0.313051
C	-5.362640	-0.837765	0.305112
C	-5.677587	0.505663	0.015832
C	-4.645356	1.364644	-0.268131
N	-3.362201	0.929585	-0.258063
H	-6.156242	-1.544677	0.521562
H	-6.701753	0.855231	0.010019
H	-4.780456	2.412305	-0.511370
O	-1.767906	-0.710675	0.047821
C	-3.691656	-2.700891	0.594499
F	-3.066385	-3.277842	-0.465368
F	-4.785447	-3.448783	0.856012
F	-2.859748	-2.823329	1.650032
C	2.278505	-1.468571	-0.142506
C	2.962515	-2.717254	0.016160
C	2.284712	-3.912566	-0.124002
C	0.914863	-3.911573	-0.425605
C	0.279906	-2.693445	-0.571181
N	0.932851	-1.518946	-0.441810
H	2.819148	-4.848025	0.002412
H	0.355972	-4.832230	-0.539129
H	-0.780309	-2.624599	-0.777408
O	2.849898	-0.340805	-0.024546
C	4.425371	-2.674997	0.336695
F	5.144082	-2.045857	-0.624105
F	4.946060	-3.921435	0.458904
F	4.682968	-2.029286	1.498034
C	2.227835	5.016316	1.214060
C	0.919029	4.746738	0.789701
C	0.696596	3.634382	-0.059038
C	1.786639	2.834480	-0.476862
C	3.080081	3.145407	-0.039425
C	3.301548	4.230353	0.803415
H	2.400617	5.857664	1.879518
H	3.910807	2.522994	-0.359149
H	4.307551	4.459448	1.143419
C	-0.707553	3.324932	-0.506181
O	-1.552015	4.202149	-0.666817
O	-1.045328	2.060827	-0.710026
H	-2.572339	1.583134	-0.486479
C	1.627395	1.634523	-1.365081
H	0.886227	1.751696	-2.158447
H	2.561489	1.394575	-1.885878
H	1.909296	0.607415	-0.636174
C	-0.190083	5.649664	1.278669
H	-0.581759	6.266813	0.465739
H	-1.039878	5.079804	1.664371
H	0.179321	6.306865	2.071342

4b-ts-o

Pd	-0.536988	0.399279	0.416819
C	-2.048480	-2.225191	0.359070
C	-1.381341	-3.093359	-0.559003
C	-2.121281	-4.010362	-1.295948
C	-3.506621	-4.074174	-1.137710
C	-4.088212	-3.194796	-0.232438
N	-3.397985	-2.298568	0.486819
H	-1.615432	-4.674455	-1.988365
H	-4.109504	-4.779669	-1.697897
H	-5.165610	-3.199921	-0.075594
O	-1.421090	-1.373930	1.134423
C	0.106438	-3.035018	-0.729717
F	0.517122	-1.875332	-1.311099
F	0.565768	-4.034048	-1.522601
F	0.772809	-3.139079	0.443691
C	2.309060	0.102237	0.116983
C	3.621313	-0.393568	0.396358
C	3.863640	-1.123387	1.545925
C	2.823537	-1.383264	2.449717
C	1.564440	-0.901215	2.143411
N	1.321784	-0.181568	1.033375
H	4.864024	-1.495267	1.739980
H	2.986834	-1.957314	3.353505
H	0.701353	-1.098330	2.769670
O	2.029442	0.792101	-0.921169
C	4.706343	-0.102238	-0.593868
F	4.432274	-0.602015	-1.821087
F	5.892540	-0.637060	-0.209900
F	4.914349	1.227361	-0.753093
C	-0.235543	5.166812	0.072420
C	-1.244077	4.428844	-0.570071
C	-1.103571	3.027854	-0.624211
C	0.048757	2.393330	-0.085728
C	1.014352	3.169664	0.580094
C	0.871538	4.552529	0.656398
H	-0.334083	6.248042	0.123651
H	1.899468	2.690289	0.984789
H	1.626066	5.153339	1.156055
C	-2.252434	2.152726	-1.059161
O	-3.121298	2.506490	-1.847131
O	-2.296200	0.980021	-0.444355
C	-2.438261	5.157600	-1.137981
H	-2.518688	5.001300	-2.216603
H	-3.374627	4.788162	-0.708829
H	-2.360148	6.230299	-0.939690
K	-3.820193	0.170571	1.565948
H	0.780701	1.460753	-0.643328

5

Pd	-0.436195	0.204000	-0.929931
C	-1.221777	-2.734672	0.063771
C	-0.434039	-3.023467	1.231726
C	-0.928009	-3.874392	2.211538
C	-2.196466	-4.441474	2.067061
C	-2.916693	-4.104934	0.925706
N	-2.470332	-3.287669	-0.037111
H	-0.323016	-4.095155	3.084960
H	-2.608628	-5.112000	2.812482
H	-3.917000	-4.510447	0.774651
O	-0.804610	-1.998225	-0.908635
C	0.916855	-2.406771	1.398321
F	1.725231	-2.581158	0.324742
F	1.585573	-2.911813	2.465020
F	0.852947	-1.056851	1.609816
C	2.496120	0.538072	-0.397355
C	3.881071	0.341912	-0.554283
C	4.323270	-0.500545	-1.564114
C	3.392023	-1.132240	-2.393389
C	2.047029	-0.888048	-2.180411
N	1.608160	-0.056582	-1.210120
H	5.386322	-0.666535	-1.695585
H	3.704826	-1.803619	-3.183741
H	1.275975	-1.363042	-2.774590
O	2.059714	1.332474	0.578516
C	4.836580	1.028481	0.380627
F	4.711636	2.374126	0.327704
F	6.120844	0.735270	0.077798
F	4.636060	0.663815	1.665892
C	-2.336074	4.351772	1.911903
C	-3.070674	3.389039	1.202032
C	-2.420197	2.665708	0.180896
C	-1.067581	2.935402	-0.148395
C	-0.369174	3.906173	0.585154
C	-0.998924	4.606341	1.612524
H	-2.822156	4.898681	2.715482
H	0.669142	4.113220	0.339182
H	-0.448139	5.352938	2.178392
C	-3.138477	1.542432	-0.523141
O	-4.332651	1.614769	-0.839518
O	-2.457833	0.444053	-0.737789
C	-0.388863	2.218704	-1.267491
H	-0.952717	2.285202	-2.205741
H	0.628447	2.580195	-1.436509
H	1.079758	1.286682	0.599455
C	-4.513651	3.141736	1.576619
H	-5.187353	3.463119	0.777569
H	-4.712974	2.077088	1.732919
H	-4.771340	3.682025	2.492345
K	-3.613823	-1.561739	-1.873631

5b-bz

Pd	-0.693044	0.316598	-0.202744
C	-0.557709	-2.937027	0.425094
C	0.708385	-3.442285	-0.038831
C	0.857996	-4.791209	-0.333132
C	-0.225655	-5.661252	-0.196434
C	-1.430225	-5.108503	0.225434
N	-1.608191	-3.814291	0.517937
H	1.821129	-5.161856	-0.668557
H	-0.139090	-6.719314	-0.415691
H	-2.311594	-5.741275	0.330357
O	-0.761166	-1.717174	0.779400
C	1.870922	-2.520939	-0.197219
F	1.654738	-1.571064	-1.156692
F	3.001452	-3.168959	-0.572861
F	2.180970	-1.848801	0.939445
C	2.194951	1.172197	-0.089378
C	3.420775	1.534302	0.499149
C	3.528823	1.503339	1.882188
C	2.428193	1.115790	2.651606
C	1.254928	0.777973	2.001033
N	1.137556	0.816281	0.655678
H	4.467158	1.774774	2.352227
H	2.482447	1.073282	3.732560
H	0.372582	0.456124	2.540790
O	2.079802	1.190218	-1.417084
C	4.575913	1.928247	-0.377934
F	4.938593	0.931217	-1.214274
F	5.664115	2.252301	0.354852
F	4.279031	3.000447	-1.146978
C	-4.406814	4.139582	0.611721
C	-4.517759	2.832846	0.138213
C	-3.406274	2.147577	-0.367596
C	-2.148064	2.794993	-0.435704
C	-2.052401	4.104831	0.063567
C	-3.162255	4.772036	0.583420
H	-5.277870	4.652849	1.008438
H	-1.090725	4.610525	0.025581
H	-3.054109	5.785634	0.960049
C	-3.606057	0.702059	-0.752863
O	-4.757327	0.229703	-0.799159
O	-2.571225	-0.060863	-0.973660
C	-0.944497	2.151336	-1.040647
H	-1.098272	1.917950	-2.101001
H	-0.058854	2.781204	-0.929934
H	1.207971	0.807693	-1.650419
K	-3.703578	-2.048010	0.458059
H	-5.468134	2.310436	0.165871

5h

Pd	-0.002835	0.468662	-0.494805
C	-3.048131	0.001659	0.224745
C	-4.228469	-0.807586	0.364349
C	-5.482230	-0.242962	0.290405
C	-5.637578	1.142432	0.066654
C	-4.505861	1.901996	-0.094606
N	-3.274587	1.340810	-0.018044
H	-6.357862	-0.874466	0.398339
H	-6.617829	1.597377	0.008505
H	-4.522615	2.968170	-0.290957
O	-1.879642	-0.459940	0.308495
C	-4.026743	-2.280579	0.559110
F	-3.344503	-2.837392	-0.475434
F	-5.204806	-2.939920	0.648809

F	-3.328226	-2.564001	1.680312
C	1.994814	-1.750709	-0.038772
C	2.532993	-3.049645	-0.106801
C	1.765674	-4.058524	-0.671640
C	0.483810	-3.766917	-1.146773
C	0.026737	-2.464289	-1.059115
N	0.780226	-1.467570	-0.537569
H	2.163101	-5.065457	-0.727804
H	-0.148989	-4.536009	-1.572592
H	-0.967061	-2.181299	-1.382206
O	2.708004	-0.785389	0.541757
C	3.904255	-3.314393	0.450485
F	4.845848	-2.550187	-0.146855
F	4.265912	-4.604328	0.275870
F	3.968485	-3.056274	1.775060
C	3.184798	4.477335	0.998294
C	1.824065	4.545449	0.657551
C	1.272287	3.511991	-0.130573
C	2.092806	2.452362	-0.596529
C	3.448692	2.424257	-0.236984
C	3.991171	3.429695	0.560443
H	3.609452	5.258743	1.622833
H	4.075149	1.609816	-0.591115
H	5.041086	3.397075	0.838410
C	-0.208131	3.514494	-0.438381
O	-0.832673	4.558177	-0.617540
O	-0.820643	2.346448	-0.459354
H	-2.424343	1.926113	-0.178614
C	1.544718	1.367702	-1.461877
H	1.030304	1.753069	-2.349026
H	2.309658	0.651604	-1.770869
H	2.178370	0.038673	0.552631
C	1.007821	5.708086	1.173408
H	0.694488	6.360638	0.354285
H	0.088065	5.373170	1.661701
H	1.590205	6.295061	1.889705

5b-o

Pd	-0.760385	0.333054	0.324873
C	-0.953776	-2.870320	0.261105
C	0.079525	-3.387615	-0.591412
C	-0.096614	-4.602222	-1.240536
C	-1.287569	-5.313027	-1.074508
C	-2.259320	-4.744012	-0.258455
N	-2.119254	-3.574937	0.381456
H	0.694084	-4.991950	-1.873244
H	-1.455287	-6.265023	-1.565096
H	-3.212284	-5.251573	-0.111258
O	-0.829591	-1.782339	0.945820
C	1.345770	-2.617870	-0.778860
F	1.141232	-1.433654	-1.428967
F	2.256739	-3.293962	-1.520951
F	1.960544	-2.305382	0.388672
C	2.196508	0.867147	0.158809
C	3.544229	0.845647	0.564568
C	3.845885	0.414850	1.848282
C	2.814667	0.017028	2.703987
C	1.514546	0.063832	2.234605
N	1.210946	0.491315	0.989453
H	4.879252	0.386870	2.174062
H	3.018104	-0.329523	3.709825
H	0.674323	-0.253917	2.839600
O	1.895077	1.278169	-1.070647
C	4.615368	1.270174	-0.400684
F	4.625621	0.503035	-1.512475
F	5.843646	1.183974	0.156469
F	4.449576	2.550051	-0.804498
C	-1.968448	4.885432	-0.498176
C	-2.891394	3.840103	-0.646648
C	-2.440447	2.526456	-0.394525
C	-1.103935	2.270844	-0.009090
C	-0.217382	3.337745	0.158787
C	-0.656080	4.641873	-0.093294
H	-2.289251	5.905086	-0.695528
H	0.805896	3.175242	0.479506
H	0.033950	5.473721	0.026223
C	-3.326877	1.322678	-0.454859
O	-4.551334	1.340190	-0.586484
O	-2.679369	0.176910	-0.305179
C	-4.309590	4.147154	-1.061173
H	-4.600775	3.572400	-1.944994
H	-5.019427	3.869724	-0.276331
H	-4.423037	5.213303	-1.279111
K	-3.817895	-1.572221	1.222864
H	0.925450	1.197325	-1.198438

6

Pd	0.678520	-0.048384	-0.736994
C	-1.015004	2.566933	0.419660
C	-2.209030	2.763580	-0.344043
C	-2.698549	4.049659	-0.542170
C	-2.017850	5.143510	-0.006960
C	-0.851028	4.881254	0.701253
N	-0.357951	3.651977	0.909614
H	-3.608483	4.193792	-1.114670
H	-2.375597	6.157791	-0.141810
H	-0.272636	5.700824	1.125699
O	-0.503445	1.393918	0.682213
C	-2.930534	1.599198	-0.944566
F	-2.182162	0.949209	-1.876794
F	-4.074075	1.967400	-1.570383
F	-3.284162	0.669676	-0.020020
C	-1.659386	-1.598102	0.324474
C	-2.664344	-2.589818	0.376228
C	-2.939086	-3.342769	-0.754379
C	-2.220659	-3.098996	-1.928168
C	-1.238354	-2.124921	-1.906492
N	-0.937418	-1.405979	-0.801863
H	-3.718316	-4.095965	-0.724798
H	-2.419435	-3.651294	-2.838769
H	-0.655703	-1.897655	-2.790901
O	-1.430127	-0.861581	1.396422
C	-3.458149	-2.752207	1.641192
F	-2.678442	-3.064844	2.700400
F	-4.378190	-3.739212	1.524537
F	-4.129822	-1.623909	1.963469

C	5.239045	-2.490368	0.609340
C	4.986480	-1.110689	0.543973
C	3.895236	-0.655040	-0.237147
C	3.095542	-1.589240	-0.951871
C	3.383252	-2.957978	-0.849890
C	4.447721	-3.408294	-0.074709
H	6.066652	-2.843050	1.218658
H	2.766557	-3.668513	-1.393878
H	4.659659	-4.471728	-0.002700
C	3.533837	0.812183	-0.248590
O	4.355940	1.677895	0.107546
O	2.322282	1.179047	-0.571187
C	1.960024	-1.172980	-1.826178
H	2.263276	-0.473126	-2.613155
H	1.459804	-2.037056	-2.267926
H	-1.110903	0.074435	1.142330
C	5.892486	-0.201705	1.343941
H	6.455668	0.473684	0.695801
H	5.325075	0.439080	2.025394
H	6.597196	-0.798164	1.931423
K	2.200918	2.816961	1.548239

6b-bz

Pd	0.857788	-0.039471	-0.653035
C	-0.946325	2.555284	0.383589
C	-2.085194	2.716446	-0.466150
C	-2.588105	3.989261	-0.711873
C	-1.974635	5.102523	-0.137099
C	-0.861138	4.873076	0.662706
N	-0.357612	3.657330	0.917703
H	-3.456167	4.107389	-1.351439
H	-2.344233	6.107099	-0.307848
H	-0.337878	5.708253	1.126114
O	-0.421911	1.396245	0.687340
C	-2.736320	1.530122	-1.102577
F	-1.917769	0.898817	-1.987461
F	-3.851819	1.863446	-1.794500
F	-3.115882	0.594286	-0.194718
C	-1.490871	-1.622893	0.335661
C	-2.470983	-2.640270	0.360622
C	-2.686871	-3.407707	-0.773014
C	-1.934419	-3.153675	-1.923094
C	-0.974858	-2.158372	-1.873389
N	-0.729628	-1.425935	-0.763650
H	-3.447060	-4.180609	-0.764638
H	-2.088378	-3.715784	-2.836315
H	-0.365098	-1.924976	-2.737753
O	-1.324299	-0.865937	1.404830
C	-3.302277	-2.817283	1.599478
F	-2.549247	-3.098485	2.686451
F	-4.187457	-3.832682	1.459895
F	-4.018149	-1.707899	1.890566
C	5.650366	-2.242414	0.682122
C	5.219335	-0.917381	0.642050
C	4.075788	-0.546650	-0.076534
C	3.353011	-1.522767	-0.805789
C	3.790973	-2.856332	-0.735061
C	4.921984	-3.217450	-0.002414
H	6.534930	-2.512459	1.251552
H	3.238934	-3.618191	-1.279802
H	5.233968	-4.257874	0.029835
C	3.631921	0.891047	0.035127
O	4.365551	1.720439	0.607301
O	2.461297	1.246962	-0.412822
C	2.180072	-1.187921	-1.666925
H	2.454790	-0.525047	-2.496717
H	1.705888	-2.090550	-2.058323
H	-1.016212	0.072835	1.147480
K	2.093426	2.764727	1.824033
H	5.752952	-0.142876	1.182556

6-Cs

Pd	0.136374	-0.552733	-0.936455
C	-0.545782	2.542049	0.010440
C	-1.581853	3.020667	-0.853592
C	-1.640764	4.371888	-1.178499
C	-0.687876	5.250122	-0.662706
C	0.288362	4.711191	0.170289
N	0.368266	3.416570	0.500372
H	-2.429584	4.733190	-1.829750
H	-0.704656	6.308674	-0.896411
H	1.054094	5.357603	0.598973
O	-0.424505	1.290372	0.362694
C	-2.608905	2.083500	-1.404681
F	-2.086772	1.175735	-2.274333
F	-3.585372	2.732280	-2.085768
F	-3.233740	1.368506	-0.433257
C	-2.458621	-1.162825	0.505838
C	-3.694640	-1.802759	0.758930
C	-4.286657	-2.563633	-0.236448
C	-3.651363	-2.679321	-1.475691
C	-2.428523	-2.054600	-1.646678
N	-1.820163	-1.339357	-0.673815
H	-5.240755	-3.044543	-0.052785
H	-4.091976	-3.244774	-2.287943
H	-1.895801	-2.121213	-2.587391
O	-1.934191	-0.392977	1.437141
C	-4.364506	-1.588929	2.085838
F	-3.593118	-1.993299	3.120907
F	-5.527584	-2.278367	2.170581
F	-4.662858	-0.287907	2.302378
C	4.060262	-3.847957	0.422931
C	4.179270	-2.523020	-0.027322
C	3.115337	-1.959422	-0.767435
C	1.972836	-2.739713	-1.078757
C	1.895700	-4.061419	-0.614617
C	2.930889	-4.611948	0.136683
H	4.867063	-4.278945	1.010167
H	1.017550	-4.656083	-0.853538
H	2.860140	-5.635151	0.496290
C	3.161146	-0.500429	-1.183309
O	4.247372	0.048802	-1.417935
O	2.044500	0.172723	-1.210835
C	0.856862	-2.186878	-1.897577
H	1.194773	-1.777861	-2.856764
H	0.074216	-2.929906	-2.064682

H	-1.348604	0.347229	1.023517
C	5.438478	-1.763145	0.325585
H	6.017094	-1.522784	-0.569376
H	5.218515	-0.803723	0.803275
H	6.061321	-2.353772	1.004540
Cs	2.986879	1.736416	1.571718

6-Li

Pd	0.839820	-0.009798	-0.721041
C	-0.738130	2.585574	0.368168
C	-2.069074	2.908730	-0.034905
C	-2.410805	4.227421	-0.297731
C	-1.444364	5.230726	-0.181325
C	-0.161050	4.847408	0.180895
N	0.193935	3.576501	0.443639
H	-3.425353	4.469337	-0.596506
H	-1.679001	6.270239	-0.377409
H	0.632401	5.587061	0.265471
O	-0.361131	1.379305	0.672650
C	-3.079358	1.813063	-0.173900
F	-2.662022	0.844159	-1.036454
F	-4.263013	2.265132	-0.648480
F	-3.340074	1.190578	0.999060
C	-1.561597	-1.577579	0.153979
C	-2.596248	-2.537425	0.086720
C	-2.743405	-3.305269	-1.057160
C	-1.867551	-3.109320	-2.128798
C	-0.873207	-2.156711	-1.999399
N	-0.703163	-1.415793	-0.879296
H	-3.543191	-4.034622	-1.117808
H	-1.957727	-3.679238	-3.045655
H	-0.174174	-1.964703	-2.804124
O	-1.438606	-0.853694	1.249395
C	-3.555049	-2.650693	1.239129
F	-2.934636	-2.981445	2.393162
F	-4.491199	-3.602060	1.007998
F	-4.215234	-1.492827	1.460150
C	5.613723	-1.900937	0.970921
C	5.147893	-0.578358	0.918497
C	4.025097	-0.283995	0.101234
C	3.410281	-1.315869	-0.672518
C	3.900872	-2.623673	-0.558853
C	4.992715	-2.917211	0.253710
H	6.469793	-2.130616	1.598737
H	3.425346	-3.415688	-1.130418
H	5.360062	-3.937401	0.323870
C	3.436195	1.092889	0.106730
O	3.882400	2.028802	0.810360
O	2.376757	1.358939	-0.616783
C	2.279273	-1.095806	-1.634713
H	2.566679	-0.449740	-2.471880
H	1.897173	-2.046273	-2.012847
H	-1.064961	0.073070	1.060220
C	5.881510	0.445322	1.753842
H	6.230832	1.285939	1.149391
H	5.233603	0.873395	2.523630
H	6.743273	-0.019913	2.240955
Li	2.057344	2.845515	0.580281

6-Na

Pd	0.782283	0.033828	-0.738893
C	-0.973593	2.568824	0.418049
C	-2.287889	2.756825	-0.113477
C	-2.777059	4.041120	-0.313308
C	-1.977225	5.144450	-0.008952
C	-0.698958	4.892775	0.471481
N	-0.203205	3.661389	0.677761
H	-3.777747	4.177356	-0.709336
H	-2.330038	6.159138	-0.151577
H	-0.027526	5.717202	0.704256
O	-0.461984	1.398669	0.675997
C	-3.128393	1.570759	-0.466688
F	-2.526591	0.776815	-1.394553
F	-4.326545	1.925602	-0.988028
F	-3.390963	0.776036	0.599445
C	-1.517612	-1.617939	0.208539
C	-2.513102	-2.619099	0.189756
C	-2.727910	-3.346278	-0.970872
C	-1.958403	-3.068320	-2.103590
C	-0.991303	-2.082543	-2.015812
N	-0.753735	-1.384700	-0.882427
H	-3.498956	-4.107918	-0.995205
H	-2.107398	-3.602539	-3.034236
H	-0.371001	-1.828538	-2.866800
O	-1.331437	-0.918284	1.313577
C	-3.356855	-2.829213	1.415898
F	-2.616744	-3.172753	2.493560
F	-4.261678	-3.819508	1.227492
F	-4.051182	-1.719213	1.748910
C	5.453976	-2.129588	0.803016
C	5.084654	-0.775424	0.777446
C	3.986118	-0.384052	-0.031203
C	3.299537	-1.355577	-0.817571
C	3.698554	-2.697422	-0.741710
C	4.767178	-3.084843	0.061501
H	6.288254	-2.432866	1.429252
H	3.167095	-3.439116	-1.331858
H	5.064238	-4.129057	0.108164
C	3.493100	1.037703	0.004468
O	4.125518	1.941406	0.592367
O	2.349255	1.346946	-0.547108
C	2.177025	-1.023503	-1.749612
H	2.475637	-0.320337	-2.535088
H	1.755688	-1.925948	-2.197093
H	-1.040818	0.034076	1.105917
C	5.885995	0.172263	1.640840
H	6.358496	0.957190	1.045616
H	5.252974	0.685678	2.369822
H	6.663443	-0.379214	2.177848
Na	2.029699	2.987616	0.990250

6b-o

Pd	0.563140	0.472508	0.179756
C	0.247613	-2.615885	0.484179
C	0.074095	-3.256038	-0.780959
C	0.676853	-4.485506	-1.018849

C	1.461522	-5.075735	-0.027506
C	1.617669	-4.375243	1.162283
N	1.050460	-3.184671	1.417359
H	0.535284	-4.976456	-1.975694
H	1.942594	-6.035166	-0.179284
H	2.238743	-4.784447	1.958350
O	-0.338927	-1.485559	0.798848
C	-0.787046	-2.638244	-1.840300
F	-2.100399	-2.651343	-1.502260
F	-0.687212	-3.290032	-3.023147
F	-0.467666	-1.340995	-2.091292
C	-2.467239	0.829396	0.344164
C	-3.691449	1.525535	0.504553
C	-3.676879	2.851429	0.900443
C	-2.449906	3.479774	1.136820
C	-1.290969	2.757676	0.925067
N	-1.284271	1.468574	0.502004
H	-4.611337	3.383923	1.036905
H	-2.394595	4.509321	1.469045
H	-0.318539	3.208553	1.071945
O	-2.509669	-0.445561	0.035468
C	-4.981246	0.787098	0.281658
F	-5.062074	0.263354	-0.961069
F	-6.049713	1.604850	0.436102
F	-5.139920	-0.234792	1.153072
C	3.337796	3.952721	-1.532795
C	3.878396	2.829757	-0.889941
C	2.977376	1.852843	-0.417878
C	1.580974	2.014959	-0.549755
C	1.071732	3.134474	-1.210445
C	1.959917	4.098145	-1.702031
H	4.009825	4.715209	-1.918131
H	0.004009	3.261926	-1.358505
H	1.571823	4.968775	-2.225419
C	3.394848	0.561998	0.206859
O	4.504888	0.324193	0.694000
O	2.432631	-0.346948	0.228693
H	-1.634479	-0.937031	0.241263
K	2.637464	-1.263157	2.668299
C	5.373300	2.688039	-0.740716
H	5.665903	2.670773	0.313352
H	5.728251	1.745137	-1.167597
H	5.890247	3.515227	-1.236044

6p

Pd	-0.657273	-0.488112	-1.406180
C	-3.664651	0.565113	0.747652
C	-2.749619	0.954420	-0.246268
C	-1.935391	2.101731	-0.015412
C	-2.048641	2.831095	1.191580
C	-2.949980	2.377488	2.174103
C	-3.753535	1.259748	1.954540
H	-4.315346	-0.287470	0.562310
H	-3.021337	2.918104	3.113992
H	-4.451984	0.932855	2.720191
C	-0.869675	2.476143	-1.027614
O	-0.492632	3.650559	-1.159697
O	-0.319085	1.517334	-1.719633
C	-1.209505	4.050682	1.505331
H	-0.145564	3.880325	1.315208
H	-1.492215	4.893592	0.869489
H	-1.340654	4.341150	2.552212
C	-2.593966	0.118919	-1.470081
H	-3.270934	-0.738420	-1.484312
H	-2.674913	0.689460	-2.400834
H	-2.458652	-1.537307	1.068957
P	2.456636	-0.698458	-0.047463
O	1.549294	-0.847353	-1.299655
O	3.910532	-1.118542	-0.264079
O	2.372103	0.743426	0.571501
O	1.807053	-1.603030	1.179054
H	1.332581	-2.430448	0.897034
K	4.836645	0.407390	1.594120
P	-1.137084	-3.090176	0.272133
O	-1.238938	-2.494330	-1.140437
O	0.170498	-3.629236	0.774754
O	-1.648474	-1.976894	1.376346
O	-2.333807	-4.195990	0.369126
H	-2.562289	-4.496107	-0.524077
K	0.079266	0.238636	2.017195
K	2.204091	2.003306	-1.809061

7-ts

Pd	-0.762967	-0.406280	0.209706
C	1.160546	2.666944	-0.321271
C	2.034605	2.783783	0.810925
C	2.011986	3.934685	1.586956
C	1.133935	4.973014	1.266696
C	0.305314	4.788870	0.163317
N	0.303040	3.693715	-0.605254
H	2.678786	4.018748	2.439095
H	1.093857	5.885269	1.850921
H	-0.402882	5.566723	-0.121053
O	1.127322	1.638217	-1.098610
C	2.953331	1.657797	1.153093
F	2.271621	0.531118	1.517100
F	3.768535	1.955051	2.196558
F	3.755482	1.293177	0.126148
C	1.959942	-1.631476	-0.371785
C	3.012009	-2.576036	-0.267969
C	2.886247	-3.657651	0.588772
C	1.719347	-3.792395	1.346345
C	0.734817	-2.829110	1.207058
N	0.833526	-1.772532	0.366126
H	3.692262	-4.377900	0.672408
H	1.579720	-4.620743	2.030807
H	-0.185269	-2.887673	1.778038
O	2.099259	-0.646599	-1.227757
C	4.264222	-2.354735	-1.067004
F	4.036423	-2.362945	-2.401248
F	5.182808	-3.323724	-0.824800
F	4.855934	-1.175286	-0.775060
C	-6.243238	-1.916619	0.271484
C	-5.549624	-1.002579	-0.534218
C	-4.385921	-0.435255	0.014656
C	-3.936430	-0.765115	1.299942

C	-4.633835	-1.684673	2.081145
C	-5.796764	-2.252117	1.553140
H	-7.144130	-2.382368	-0.118097
H	-4.283495	-1.948933	3.074197
H	-6.357829	-2.971205	2.143055
C	-3.493451	0.539734	-0.644959
O	-3.673418	1.087253	-1.732450
O	-2.440391	0.865503	0.121317
C	-2.704628	-0.070946	1.740389
H	-2.822929	0.879929	2.254806
H	-1.946375	-0.696839	2.211693
H	1.646172	0.262970	-1.015473
C	-6.032226	-0.666499	-1.920708
H	-6.263217	0.399425	-2.009247
H	-5.260353	-0.875637	-2.667924
H	-6.927874	-1.242307	-2.168777
K	-1.162970	2.113783	-2.374282

7-ts-a

Pd	-0.307136	-1.240295	-1.573159
C	0.182415	0.213172	1.126206
C	0.168153	0.283885	2.551022
C	0.915416	1.255261	3.198383
C	1.664691	2.159063	2.438460
C	1.610588	2.037700	1.055768
N	0.890473	1.107962	0.401711
H	0.916250	1.304721	4.282014
H	2.269211	2.928225	2.905298
H	2.183513	2.721311	0.431366
O	-0.505380	-0.728966	0.532157
C	-0.643222	-0.714761	3.318211
F	-1.955933	-0.681364	2.977531
F	-0.583967	-0.490766	4.655279
F	-0.228814	-1.991110	3.125371
C	-3.161944	0.308223	-1.037255
C	-4.374387	0.980069	-1.335911
C	-4.641625	1.343537	-2.646163
C	-3.710588	1.041938	-3.645106
C	-2.549115	0.382576	-3.276571
N	-2.271205	0.021519	-2.005517
H	-5.566318	1.857477	-2.884000
H	-3.885798	1.306441	-4.681267
H	-1.794760	0.119954	-4.010863
O	-2.935890	-0.040657	0.214939
C	-5.335929	1.291348	-0.225000
F	-5.769162	0.177023	0.405525
F	-6.434934	1.935071	-0.687032
F	-4.783481	2.087982	0.719103
C	3.783699	-1.818677	2.549320
C	3.965869	-1.025854	1.406155
C	3.193469	-1.347415	0.276854
C	2.292796	-2.425815	0.288116
C	2.092401	-3.169331	1.454193
C	2.857108	-2.864333	2.579799
H	4.357867	-1.589209	3.442912
H	1.361758	-3.971629	1.476255
H	2.719258	-3.433674	3.494228
C	3.110854	-0.549049	-0.973889
O	3.723300	0.505654	-1.195779
O	2.267789	-1.047180	-1.849805
C	1.590928	-2.731464	-0.965895
H	2.139514	-3.208071	-1.770856
H	0.585482	-3.161383	-0.847296
H	-1.989377	-0.376300	0.365741
C	4.923013	0.137537	1.427716
H	5.657712	0.065685	0.620784
H	4.389034	1.079560	1.274100
H	5.451286	0.182405	2.384343
K	1.269010	1.373348	-2.515037
O	-0.072587	-1.750647	-3.567237
O	0.287304	-0.725770	-4.287807

7-ts-b

Pd	0.145905	-0.114504	-1.823041
C	0.757861	1.043932	0.932308
C	0.598573	1.279468	2.332324
C	1.419879	2.194287	2.972518
C	2.389870	2.879206	2.233997
C	2.468737	2.608274	0.872154
N	1.687960	1.729028	0.223772
H	1.303132	2.371059	4.036612
H	3.052979	3.600423	2.697808
H	3.206580	3.128410	0.261941
O	0.029955	0.148314	0.332685
C	-0.460840	0.529237	3.075004
F	-1.702369	0.772911	2.579862
F	-0.495067	0.859983	4.389554
F	-0.289007	-0.815033	3.018398
C	-2.841761	-0.223660	-0.945857
C	-4.242142	-0.172071	-1.129570
C	-4.749039	0.368172	-2.303426
C	-3.869818	0.863371	-3.270296
C	-2.507019	0.771418	-3.034465
N	-2.009808	0.217512	-1.907667
H	-5.822145	0.415825	-2.451700
H	-4.235174	1.309474	-4.187845
H	-1.749776	1.155073	-3.717657
O	-2.359320	-0.728559	0.179402
C	-5.143481	-0.675959	-0.039977
F	-4.940111	-1.988039	0.224543
F	-6.449117	-0.541358	-0.374177
F	-4.964275	-0.009589	1.122502
C	2.848305	-2.379206	2.599159
C	3.400053	-1.619133	1.554957
C	2.743959	-1.670674	0.315041
C	1.582542	-2.441855	0.132954
C	1.026918	-3.155144	1.196895
C	1.685669	-3.132456	2.427066
H	3.324370	-2.350368	3.575257
H	0.108001	-3.717425	1.064418
H	1.273538	-3.683208	3.267165
C	3.095002	-0.857539	-0.873307
O	4.120445	-0.189780	-1.018760
O	2.193751	-0.927440	-1.849214
C	0.986548	-2.458655	-1.203903
H	1.489392	-2.977628	-2.012053

H	-0.100127	-2.514952	-1.257306
H	-1.424865	-0.391398	0.365209
C	4.610469	-0.754828	1.793445
H	5.433614	-1.030328	1.128518
H	4.382794	0.295148	1.584601
H	4.947445	-0.842336	2.829868
K	2.727042	1.612943	-2.838512
O	0.324885	-0.021222	-3.780243
O	0.307697	1.355806	-3.903547

7-ts-c

Pd	0.092818	0.500034	-1.033594
C	-1.975511	-2.794404	-0.306148
C	-1.629986	-3.230505	1.027722
C	-2.610795	-3.674952	1.900712
C	-3.949442	-3.691283	1.495032
C	-4.227938	-3.233416	0.209550
N	-3.309724	-2.801559	-0.661144
H	-2.332505	-4.004844	2.897008
H	-4.740822	-4.034974	2.151541
H	-5.259889	-3.209704	-0.142983
O	-1.112269	-2.395882	-1.153515
C	-0.203689	-3.177213	1.454637
F	0.624733	-3.899653	0.651907
F	-0.019429	-3.658644	2.712629
F	0.297430	-1.909354	1.463135
C	3.008686	0.224129	-0.194669
C	4.287384	-0.362240	-0.259568
C	4.400790	-1.677977	-0.692268
C	3.248107	-2.387309	-1.034193
C	2.023355	-1.743320	-0.958099
N	1.909393	-0.448109	-0.575389
H	5.377943	-2.145104	-0.739667
H	3.295947	-3.423272	-1.348313
H	1.084541	-2.241319	-1.178712
O	2.898664	1.482834	0.237566
C	5.487796	0.429964	0.172079
F	5.635794	1.565827	-0.549599
F	6.630167	-0.280717	0.024536
F	5.416385	0.797940	1.471125
C	-2.693494	4.329821	1.899658
C	-3.176873	3.281672	1.102193
C	-2.366414	2.883530	0.025783
C	-1.136571	3.499190	-0.245413
C	-0.667279	4.534301	0.566377
C	-1.466841	4.946228	1.639786
H	-3.283285	4.657317	2.750581
H	0.287538	5.010398	0.364083
H	-1.122258	5.747757	2.286187
C	-2.647571	1.780079	-0.917480
O	-3.699279	1.162385	-1.046889
O	-1.605597	1.540682	-1.736123
C	-0.392712	2.955168	-1.403793
H	-0.632103	3.352124	-2.387165
H	0.679715	2.826019	-1.267889
H	2.017934	1.634124	0.663950
C	-4.483158	2.603620	1.416294
H	-5.193458	2.718447	0.591946
H	-4.340919	1.527037	1.553344
H	-4.926542	3.019181	2.324847
O	0.816458	2.013624	1.924097
O	-0.137507	0.929331	2.073552
H	0.231748	2.766727	1.717000
H	-0.125919	0.537869	1.159241
K	-2.789554	-1.019188	-2.680853

7-ts-c-rea

Pd	-0.445182	0.176915	-1.043293
C	-1.004965	-2.838203	-0.012034
C	-0.048811	-3.252068	0.980386
C	-0.427319	-4.113465	2.004631
C	-1.739483	-4.582603	2.064201
C	-2.615717	-4.151221	1.071073
N	-2.284770	-3.319866	0.076491
H	0.302448	-4.416830	2.748331
H	-2.068940	-5.255917	2.847072
H	-3.650677	-4.492502	1.076602
O	-0.707463	-2.059673	-0.992541
C	1.348263	-2.733726	0.928628
F	1.926622	-2.820122	-0.286293
F	2.171382	-3.358866	1.799512
F	1.405645	-1.393139	1.274852
C	2.449468	0.711722	-0.384959
C	3.846556	0.687097	-0.583623
C	4.359972	0.003126	-1.674767
C	3.487525	-0.650550	-2.549823
C	2.130397	-0.582843	-2.295384
N	1.618099	0.097334	-1.244425
H	5.431598	-0.027053	-1.834150
H	3.854338	-1.203572	-3.405973
H	1.406000	-1.079787	-2.929119
O	1.978642	1.368384	0.667160
C	4.744523	1.372365	0.408447
F	4.483994	2.696076	0.500179
F	6.046484	1.248089	0.063085
F	4.612751	0.856023	1.649961
C	-2.616380	4.216334	1.726095
C	-3.283904	3.207107	1.015351
C	-2.583265	2.525910	-0.006069
C	-1.244542	2.881291	-0.326187
C	-0.617419	3.899323	0.409746
C	-1.298341	4.559395	1.430405
H	-3.138182	4.726859	2.530749
H	0.410951	4.162011	0.178768
H	-0.798902	5.337432	2.000924
C	-3.232324	1.364674	-0.722003
O	-4.433336	1.368560	-1.020134
O	-2.485863	0.320391	-0.968975
C	-0.491942	2.183782	-1.407551
H	-1.021347	2.212108	-2.367496
H	0.513678	2.590159	-1.534838
H	1.035224	1.166466	0.872260
C	-4.705992	2.863568	1.390965
H	-5.399446	3.144712	0.593872
H	-4.833783	1.787385	1.541157
H	-4.994868	3.381529	2.309974

O	-0.419714	1.137109	2.085177
O	-0.976084	-0.179721	2.363330
H	-1.134789	1.530945	1.550621
H	-0.347002	-0.741617	1.878684
K	-3.612126	-1.816933	-1.830493

7-ts-d

Pd	0.018108	-0.757745	-1.071154
C	2.550474	0.891589	-0.090843
C	3.300250	-0.081408	0.652941
C	4.226422	0.341230	1.599834
C	4.420400	1.704356	1.829040
C	3.650978	2.591864	1.081826
N	2.750781	2.219877	0.165244
H	4.799368	-0.395933	2.153077
H	5.139074	2.062301	2.557280
H	3.759826	3.665901	1.229509
O	1.702354	0.598876	-1.019986
C	3.083729	-1.546217	0.442818
F	1.929283	-1.991511	1.035199
F	4.077747	-2.294054	0.981282
F	2.993970	-1.900304	-0.859700
C	-5.180160	-0.570778	1.250356
C	-4.518319	0.549353	0.723942
C	-3.114110	0.506779	0.700555
C	-2.406781	-0.602947	1.195764
C	-3.088452	-1.725676	1.673713
C	-4.482584	-1.690295	1.712366
H	-6.266490	-0.573585	1.274092
H	-2.540145	-2.594884	2.023710
H	-5.032768	-2.545979	2.092616
C	-2.222380	1.530270	0.092483
O	-2.578300	2.613292	-0.385011
O	-0.953608	1.169465	0.106044
C	-0.941057	-0.518941	1.201153
H	-0.461244	0.136931	1.919933
H	-0.401364	-1.461253	1.086464
C	-5.300365	1.721990	0.191236
H	-5.043191	2.642469	0.723548
H	-5.065979	1.904251	-0.861795
H	-6.374970	1.545640	0.290340
O	-1.466026	-2.104154	-1.508560
O	-0.718050	-2.984249	-2.087754
K	0.454276	3.034276	-1.219178

7-ts-e

Pd	-0.033937	-0.708688	-1.063707
C	2.496242	0.894028	-0.093477
C	3.222691	-0.071320	0.679568
C	4.105891	0.359430	1.662966
C	4.279425	1.724065	1.899145
C	3.534177	2.604309	1.120900
N	2.673996	2.222408	0.169615
H	4.662022	-0.373142	2.238770
H	4.965133	2.087897	2.655730
H	3.626917	3.679375	1.270987
O	1.687217	0.595464	-1.060805
C	3.031228	-1.539021	0.459271
F	1.857223	-1.992260	1.003087
F	4.008650	-2.274955	1.045414
F	3.007243	-1.889633	-0.842841
C	-5.255722	-0.492198	1.072407
C	-4.468874	0.594763	0.657765
C	-3.075799	0.422478	0.701040
C	-2.499849	-0.780724	1.147073
C	-3.302500	-1.866388	1.505661
C	-4.688387	-1.700488	1.483432
H	-6.337340	-0.395074	1.043418
H	-2.853932	-2.805411	1.813564
H	-5.332893	-2.525234	1.772318
C	-2.070725	1.391660	0.202760
O	-2.295975	2.543836	-0.173998
O	-0.829746	0.914947	0.215335
C	-1.040121	-0.842190	1.234714
H	-0.527599	-0.277957	2.006262
H	-0.578201	-1.802146	1.011606
C	-5.113468	1.864872	0.168071
H	-4.810862	2.721486	0.777404
H	-4.803616	2.092870	-0.856423
H	-6.202826	1.779125	0.196880
O	-1.238384	-2.226912	-1.597882
O	0.025740	-2.418999	-2.104224
K	0.392749	3.032962	-1.193337

7-ts-f

Pd	0.194842	-0.093247	-0.569663
C	-1.133786	-2.660848	0.328016
C	-0.190112	-3.297303	1.195597
C	-0.637387	-4.004727	2.303321
C	-2.008631	-4.090826	2.557011
C	-2.869129	-3.466698	1.658243
N	-2.464749	-2.777365	0.583043
H	0.079318	-4.486622	2.960323
H	-2.394153	-4.630959	3.414137
H	-3.946575	-3.520356	1.808021
O	-0.767527	-1.998486	-0.731479
C	1.267265	-3.217586	0.874681
F	1.564480	-3.690428	-0.357324
F	2.026803	-3.910348	1.754942
F	1.742749	-1.930721	0.904439
C	-2.639953	3.966085	1.928929
C	-3.343760	3.139810	1.041634
C	-2.590375	2.410742	0.103185
C	-1.188830	2.499059	0.063972
C	-0.506319	3.301969	0.984038
C	-1.244493	4.045940	1.903674
H	-3.194910	4.542756	2.664154
H	0.578706	3.348899	0.972898
H	-0.729906	4.684940	2.615764
C	-3.131784	1.431781	-0.882325
O	-4.312747	1.039144	-0.909657
O	-2.218810	0.981082	-1.695117
C	-0.494082	1.712470	-0.988228
H	-0.418717	2.188869	-1.964366
H	0.716086	1.546861	-0.647642
C	-4.846688	3.046544	1.114685

H	-5.306733	3.281119	0.150466
H	-5.163632	2.028689	1.360381
H	-5.239067	3.733026	1.870426
K	-3.324844	-1.472287	-1.741001

8

Pd	-0.075596	-0.852998	-0.947998
C	0.252163	1.571695	1.365782
C	0.241616	2.587886	0.353884
C	1.433956	3.145621	-0.082152
C	2.643546	2.696281	0.459363
C	2.580478	1.693577	1.424660
N	1.445771	1.139644	1.868773
H	1.420268	3.923048	-0.839556
H	3.596348	3.104353	0.141631
H	3.498922	1.303030	1.861266
O	-0.828635	1.041974	1.824773
C	-1.073426	3.051582	-0.181964
F	-1.807873	2.044320	-0.730296
F	-0.926365	3.984899	-1.158411
F	-1.859682	3.611277	0.770609
C	-3.035917	-0.721047	-0.207415
C	-4.422548	-0.895587	-0.423478
C	-4.886756	-1.239163	-1.685137
C	-3.965047	-1.399725	-2.721227
C	-2.619949	-1.226113	-2.439194
N	-2.145912	-0.910645	-1.207782
H	-5.949384	-1.368211	-1.856357
H	-4.280218	-1.659179	-3.725125
H	-1.869834	-1.350487	-3.210910
O	-2.630873	-0.416825	1.014665
C	-5.369434	-0.686047	0.724026
F	-5.134906	-1.544413	1.745376
F	-6.657133	-0.877522	0.345213
F	-5.294811	0.562764	1.234894
C	6.654619	-0.700927	0.093932
C	5.475114	-1.265536	0.591776
C	4.307037	-0.975357	-0.134846
C	4.307570	-0.172714	-1.277279
C	5.485710	0.383174	-1.760268
C	6.660283	0.104002	-1.055186
H	7.589282	-0.889576	0.614483
H	5.496465	1.011344	-2.645228
H	7.601520	0.521429	-1.401046
C	2.931863	-1.390790	0.144183
O	2.462978	-2.082425	1.026017
O	2.123035	-0.838378	-0.828223
C	2.901517	-0.046483	-1.777621
H	2.516616	0.975181	-1.744223
H	2.740250	-0.471598	-2.771875
H	-1.857946	0.271542	1.114093
C	5.450161	-2.117550	1.830972
H	4.802771	-1.674448	2.595466
H	5.044522	-3.111796	1.617146
H	6.453943	-2.231270	2.247650
K	0.103917	-1.408727	2.282231

9-ts

Pd	-0.897853	-0.620494	-0.252375
C	0.911034	1.973443	0.469703
C	1.683600	3.100715	0.052352
C	2.269160	3.928880	0.995973
C	2.096115	3.642917	2.353871
C	1.332351	2.530786	2.681890
N	0.747632	1.710307	1.788151
H	2.855641	4.783887	0.676727
H	2.538668	4.261472	3.126296
H	1.181280	2.276999	3.731515
O	0.393878	1.193406	-0.441312
C	1.841015	3.361535	-1.414654
F	2.429322	2.324914	-2.064200
F	2.613256	4.451793	-1.653360
F	0.657775	3.583728	-2.034983
C	1.993889	-1.544624	-0.279350
C	3.113897	-2.368112	-0.005828
C	2.965274	-3.481252	0.803489
C	1.707356	-3.774731	1.340226
C	0.651202	-2.939130	1.029752
N	0.778593	-1.849717	0.233869
H	3.821746	-4.109809	1.018632
H	1.549761	-4.635519	1.978876
H	-0.343782	-3.136731	1.410312
O	2.159957	-0.496920	-1.057051
C	4.451866	-1.989661	-0.579699
F	4.437071	-1.951593	-1.929457
F	5.409139	-2.876354	-0.218784
F	4.859067	-0.773745	-0.151338
C	-5.345884	-0.042199	-2.156365
C	-5.022925	0.257143	-0.828885
C	-3.990733	-0.467405	-0.206388
C	-3.291181	-1.482145	-0.917205
C	-3.617115	-1.722050	-2.273896
C	-4.644199	-1.016670	-2.881085
H	-6.150023	0.506729	-2.640163
H	-3.066543	-2.482640	-2.820966
H	-4.908093	-1.215671	-3.915642
C	-3.518626	-0.099211	1.196840
O	-3.703870	-0.908161	2.140371
O	-2.886813	1.001825	1.256380
C	-2.188648	-2.197674	-0.269861
H	-2.353257	-2.437847	0.783537
H	-1.777975	-3.035129	-0.837170
H	1.432891	0.216085	-0.922498
C	-5.749306	1.345449	-0.076342
H	-6.184468	0.960223	0.853262
H	-5.052960	2.141874	0.204765
H	-6.555001	1.776459	-0.676757
K	-1.251201	0.125549	3.086591

9b-ts-bz

Pd	-1.068810	-0.525823	-0.213013
C	0.860572	1.998342	0.449009
C	1.688883	3.070075	-0.005311
C	2.292230	3.916887	0.910243
C	2.081019	3.704434	2.276117
C	1.262253	2.643980	2.640374

N	0.659559	1.806963	1.774304
H	2.920763	4.730335	0.563637
H	2.534937	4.340345	3.027570
H	1.078070	2.449641	3.697203
O	0.325644	1.198869	-0.434739
C	1.881863	3.254601	-1.479640
F	2.434764	2.164001	-2.069300
F	2.704744	4.298253	-1.754308
F	0.719752	3.497751	-2.131306
C	1.755814	-1.628539	-0.222643
C	2.817647	-2.525863	0.051595
C	2.598471	-3.615694	0.876441
C	1.328540	-3.812430	1.429027
C	0.330061	-2.910238	1.115015
N	0.525823	-1.843880	0.301652
H	3.410168	-4.300970	1.092026
H	1.117420	-4.650982	2.081666
H	-0.672700	-3.032322	1.506720
O	1.989424	-0.602070	-1.010780
C	4.172209	-2.251857	-0.542497
F	4.142963	-2.233928	-1.892616
F	5.068776	-3.198388	-0.178062
F	4.669966	-1.061844	-0.139018
C	-5.454180	0.244480	-2.231690
C	-5.104922	0.559595	-0.918927
C	-4.142017	-0.191642	-0.239694
C	-3.504767	-1.280742	-0.899586
C	-3.838041	-1.549390	-2.248521
C	-4.811744	-0.806842	-2.902747
H	-6.215323	0.827686	-2.742483
H	-3.331734	-2.363954	-2.759977
H	-5.073533	-1.037310	-3.931165
C	-3.672225	0.214408	1.151760
O	-3.907847	-0.548753	2.122709
O	-2.990423	1.285729	1.175628
C	-2.449156	-2.029280	-0.212102
H	-2.643052	-2.223537	0.845815
H	-2.076400	-2.906757	-0.744001
H	1.309894	0.158836	-0.885551
K	-1.445996	0.377216	3.084889
H	-5.575147	1.399640	-0.415854

9b-ts-o

Pd	-0.528419	-0.426359	0.459972
C	1.881235	-2.059890	-0.687001
C	2.471133	-2.696628	0.441338
C	2.999968	-3.975274	0.305283
C	2.934719	-4.614709	-0.933232
C	2.320335	-3.934318	-1.980930
N	1.805001	-2.700930	-1.875152
H	3.454927	-4.464234	1.160182
H	3.338075	-5.610394	-1.077484
H	2.230875	-4.402331	-2.959513
O	1.371934	-0.849033	-0.631232
C	2.478355	-2.002434	1.765610
F	3.000175	-0.755127	1.708371
F	3.174863	-2.680938	2.700903
F	1.209869	-1.853385	2.271375
C	0.897570	2.179318	-0.102553
C	1.045370	3.564813	-0.339130
C	-0.048536	4.301750	-0.765728
C	-1.273008	3.659084	-0.969411
C	-1.365105	2.306820	-0.695114
N	-0.310142	1.586099	-0.237802
H	0.059330	5.363438	-0.956019
H	-2.141898	4.196732	-1.329473
H	-2.282233	1.755689	-0.857394
O	1.954955	1.477635	0.265485
C	2.398172	4.196694	-0.159823
F	2.875380	4.031551	1.092964
F	2.355111	5.528435	-0.395173
F	3.315789	3.675362	-1.005394
C	-4.994223	0.044264	1.876322
C	-4.680479	-0.421308	0.590723
C	-3.325189	-0.480083	0.222228
C	-2.341303	-0.085822	1.133719
C	-2.648829	0.409381	2.397794
C	-3.999903	0.455014	2.768561
H	-6.037384	0.094002	2.178860
H	-1.873352	0.747385	3.080004
H	-4.274657	0.822018	3.754112
C	-2.829097	-0.942288	-1.145945
O	-2.984775	-0.172004	-2.126562
O	-2.193671	-2.048625	-1.149925
H	1.850666	0.499555	0.028967
K	-0.421807	-1.154333	-2.928695
C	-5.749756	-0.836295	-0.390092
H	-5.723772	-0.198706	-1.280633
H	-5.592419	-1.865701	-0.733253
H	-6.747394	-0.774181	0.053571

10

Pd	-0.189342	-0.066811	-1.445740
C	-1.507270	1.395972	1.011890
C	-2.839387	1.774275	0.731906
C	-3.823946	1.478737	1.666519
C	-3.471790	0.817483	2.845096
C	-2.136640	0.479205	3.026804
N	-1.167068	0.751399	2.133436
H	-4.852590	1.761287	1.472988
H	-4.212586	0.569561	3.596254
H	-1.820665	-0.043201	3.927239
O	-0.560069	1.682590	0.108874
C	-3.164084	2.502775	-0.540324
F	-2.541540	3.700270	-0.616439
F	-4.492275	2.736924	-0.653966
F	-2.799220	1.806494	-1.647506
C	2.481648	0.835561	-0.374503
C	3.906730	0.784836	-0.292416
C	4.662882	0.496198	-1.415313
C	4.017812	0.244679	-2.632052
C	2.633294	0.243349	-2.646681
N	1.878417	0.513056	-1.559167
H	5.744828	0.462104	-1.344592
H	4.574966	0.024091	-3.534538
H	2.084985	0.003510	-3.551476

O	1.771611	1.143215	0.657156
C	4.539439	0.996994	1.046626
F	4.252935	2.195316	1.596113
F	5.884589	0.891631	0.998549
F	4.119057	0.055538	1.957263
C	-3.423595	-1.830067	0.203310
C	-2.268388	-2.523894	0.626841
C	-1.174501	-2.626032	-0.236119
C	-1.251999	-2.036729	-1.546435
C	-2.420724	-1.306539	-1.920180
C	-3.504294	-1.216111	-1.038660
H	-4.268123	-1.763548	0.884229
H	-2.495060	-0.898862	-2.924568
H	-4.394967	-0.668018	-1.327278
C	0.123496	-3.321867	0.190908
O	0.504488	-4.278301	-0.507301
O	0.678006	-2.822466	1.218209
C	-0.085720	-1.883584	-2.396003
H	0.805366	-2.455173	-2.153824
H	-0.251381	-1.722019	-3.462044
H	0.440388	1.478523	0.434786
C	-2.225562	-3.098746	2.022827
H	-1.708725	-4.061385	2.051180
H	-1.683587	-2.427279	2.697175
H	-3.235983	-3.232632	2.420808
K	1.349201	-0.753666	2.461682

10b-bz

Pd	0.298712	0.673914	-1.275393
C	1.700067	-1.517289	0.562486
C	3.058665	-1.679275	0.210816
C	4.007207	-1.719430	1.224909
C	3.593239	-1.593000	2.552951
C	2.236392	-1.422320	2.797914
N	1.300757	-1.376456	1.831084
H	5.054951	-1.847953	0.977285
H	4.303963	-1.621258	3.370758
H	1.871933	-1.309514	3.816782
O	0.786534	-1.492404	-0.416315
C	3.448561	-1.820402	-1.231986
F	2.879517	-2.898666	-1.814079
F	4.787519	-1.949485	-1.375981
F	3.082644	-0.741534	-1.973926
C	-2.294678	-0.721301	-0.690475
C	-3.719903	-0.799010	-0.652377
C	-4.478468	-0.230508	-1.660916
C	-3.836413	0.428393	-2.716473
C	-2.455986	0.529733	-2.677307
N	-1.699975	-0.009393	-1.696199
H	-5.560731	-0.295141	-1.624324
H	-4.395547	0.877744	-3.528213
H	-1.913610	1.073062	-3.443836
O	-1.576366	-1.281096	0.222507
C	-4.354469	-1.463845	0.528253
F	-3.977108	-2.749960	0.689516
F	-5.702617	-1.449230	0.458770
F	-4.028428	-0.827660	1.702655
C	3.117067	1.899469	1.273553
C	1.853753	2.277012	1.766810
C	0.815413	2.642049	0.922114
C	1.050814	2.628122	-0.498620
C	2.322203	2.187251	-0.983283
C	3.351156	1.839240	-0.092601
H	3.904232	1.629214	1.971077
H	2.520361	2.219651	-2.051533
H	4.313565	1.521732	-0.479711
C	-0.563251	2.988989	1.504898
O	-1.173635	3.949413	0.998650
O	-0.931983	2.231122	2.456401
C	-0.018781	2.697059	-1.474583
H	-0.997819	3.017924	-1.134669
H	0.240392	2.977106	-2.496491
H	-0.224656	-1.463369	-0.067780
K	-1.312226	-0.260847	2.647902
H	1.663357	2.264707	2.835359

10b-o

Pd	-0.118000	-0.710098	1.325545
C	-1.056828	-0.400407	-1.888272
C	-2.360962	-0.939623	-1.759768
C	-3.454359	-0.101653	-1.923095
C	-3.240420	1.250354	-2.204629
C	-1.927791	1.697183	-2.283973
N	-0.850666	0.903326	-2.128939
H	-4.458528	-0.494969	-1.812395
H	-4.067907	1.938035	-2.333737
H	-1.719116	2.748351	-2.469956
O	-0.027780	-1.225055	-1.752826
C	-2.531264	-2.387322	-1.411517
F	-2.058602	-3.211998	-2.373906
F	-3.832713	-2.707601	-1.221173
F	-1.882602	-2.733501	-0.263704
C	2.722399	-0.626485	0.088901
C	4.135478	-0.446483	0.259664
C	4.789581	-0.992880	1.349163
C	4.055841	-1.712756	2.301235
C	2.682433	-1.788816	2.132476
N	2.020972	-1.255996	1.084818
H	5.860308	-0.856656	1.461812
H	4.532159	-2.165306	3.162654
H	2.062479	-2.284953	2.875595
O	2.127256	-0.175260	-0.950508
C	4.855806	0.384128	-0.751868
F	4.776439	-0.099322	-2.009649
F	6.169230	0.522944	-0.463407
F	4.348088	1.663666	-0.810964
C	-4.476250	1.129333	1.631150
C	-3.381407	1.949817	1.326523
C	-2.107609	1.344223	1.295294
C	-1.955258	-0.031029	1.540190
C	-3.054735	-0.833537	1.837396
C	-4.319568	-0.236453	1.885093
H	-5.470248	1.567559	1.665473
H	-2.947564	-1.902112	2.002111
H	-5.192423	-0.843354	2.113350
C	-0.838811	2.040917	0.952316

O	0.243200	1.287106	1.072553
O	-0.736848	3.206401	0.551061
H	0.836229	-0.753817	-1.459183
K	1.497569	2.362021	-1.115160
C	-3.584954	3.412872	1.025857
H	-2.964237	4.044344	1.667889
H	-3.288494	3.642858	-0.002178
H	-4.633748	3.693298	1.160588

11b-ts-o

Pd	-0.055277	-1.921051	0.175738
C	1.235073	1.658842	1.044438
C	2.608698	1.560373	1.379539
C	3.486279	2.516321	0.886993
C	3.000727	3.529073	0.055784
C	1.648904	3.511352	-0.262431
N	0.775412	2.605862	0.213543
H	4.539211	2.461858	1.140030
H	3.656709	4.290562	-0.349739
H	1.236739	4.258580	-0.937398
O	0.395369	0.784121	1.577889
C	3.090553	0.415098	2.217969
F	2.540541	0.400270	3.453518
F	4.435866	0.452525	2.386619
F	2.808168	-0.787420	1.654234
C	-2.663725	-0.298105	0.576582
C	-4.085965	-0.132268	0.499418
C	-4.932434	-1.209523	0.695511
C	-4.386731	-2.470932	0.963639
C	-3.005477	-2.589696	0.975696
N	-2.160217	-1.555568	0.777746
H	-6.007151	-1.070434	0.638833
H	-5.014956	-3.337347	1.132448
H	-2.536579	-3.555133	1.142733
O	-1.874471	0.698651	0.431400
C	-4.612963	1.225035	0.165318
F	-4.229520	2.185895	1.033663
F	-5.962549	1.255672	0.110484
F	-4.178391	1.650578	-1.072106
C	3.706268	-2.122248	-1.166457
C	3.282296	-0.812490	-1.520028
C	1.920317	-0.633766	-1.677351
C	1.011728	-1.682109	-1.420575
C	1.413794	-3.028705	-1.283973
C	2.820707	-3.186917	-1.089303
H	4.768113	-2.306632	-1.027105
H	0.776795	-3.891607	-1.443612
H	3.202110	-4.190069	-0.916825
C	1.022358	0.294177	-2.401406
O	-0.070954	-0.433598	-2.550833
O	1.177743	1.450031	-2.795978
H	-0.521833	0.789196	1.127179
K	-1.376249	2.018766	-1.724506
C	4.266365	0.298977	-1.760095
H	5.024142	0.002220	-2.493777
H	3.761675	1.200214	-2.114400
H	4.790993	0.542078	-0.828882

11-ts

Pd	-0.528598	2.421402	-0.513915
C	1.510274	-1.692646	-0.582066
C	2.767502	-1.902050	-1.204318
C	3.526542	-3.000359	-0.826429
C	3.033552	-3.871371	0.148767
C	1.790706	-3.587834	0.699476
N	1.036626	-2.528634	0.353245
H	4.492337	-3.172015	-1.288679
H	3.597528	-4.739690	0.469144
H	1.371157	-4.240232	1.463062
O	0.802417	-0.636159	-0.950681
C	3.264671	-0.926199	-2.227871
F	2.411669	-0.782078	-3.267408
F	4.455945	-1.309681	-2.748737
F	3.452071	0.312347	-1.704831
C	-2.409064	0.059485	-0.634672
C	-3.632519	-0.646974	-0.857701
C	-4.575286	-0.161705	-1.749416
C	-4.319674	1.035105	-2.427701
C	-3.133644	1.701561	-2.150823
N	-2.205161	1.252280	-1.282569
H	-5.498244	-0.708049	-1.914606
H	-5.026453	1.445722	-3.138959
H	-2.904205	2.645688	-2.636361
O	-1.517174	-0.392145	0.169182
C	-3.862774	-1.895624	-0.071125
F	-2.883412	-2.827409	-0.234285
F	-5.028999	-2.498201	-0.379429
F	-3.899643	-1.654166	1.281013
C	3.118741	2.381323	0.494876
C	2.722462	1.298666	1.325425
C	1.422550	1.307473	1.820999
C	0.521151	2.345526	1.459417
C	0.958726	3.491195	0.727543
C	2.292372	3.459899	0.237621
H	4.129074	2.377315	0.095318
H	0.396613	4.424482	0.740279
H	2.668809	4.309263	-0.325168
C	0.799260	0.397356	2.845293
O	-0.328337	0.852716	3.313546
O	1.285102	-0.699004	3.185625
C	-0.857551	2.163854	1.876283
H	-1.434248	1.305424	1.572550
H	-1.406924	2.998812	2.308263
H	-0.079298	-0.553954	-0.462227
C	3.735248	0.228870	1.644667
H	3.270549	-0.628181	2.128347
H	4.227649	-0.092233	0.721663
H	4.515048	0.624118	2.308317
K	-1.072019	-1.880546	2.239825

11b-ts-bz

Pd	-0.037314	-1.934932	0.825920
C	-1.506192	0.909215	-1.507205
C	-2.841697	0.547537	-1.807888
C	-3.840249	1.499836	-1.662116
C	-3.503018	2.777621	-1.206626

C	-2.170505	3.029174	-0.901260
N	-1.183158	2.127940	-1.052225
H	-4.869422	1.240466	-1.885066
H	-4.255297	3.546738	-1.074427
H	-1.873014	3.995067	-0.502689
O	-0.558754	0.000550	-1.686831
C	-3.147747	-0.859858	-2.221624
F	-2.539940	-1.208263	-3.379601
F	-4.477770	-1.048846	-2.404560
F	-2.752656	-1.765860	-1.290923
C	2.357493	-0.596733	-0.461032
C	3.737317	-0.415910	-0.781119
C	4.566888	-1.511507	-0.956208
C	4.035072	-2.797513	-0.807759
C	2.697922	-2.916217	-0.451395
N	1.874812	-1.864356	-0.265832
H	5.613172	-1.366937	-1.205001
H	4.645661	-3.681947	-0.945793
H	2.252145	-3.894354	-0.295805
O	1.569864	0.410404	-0.332500
C	4.240378	0.987715	-0.877040
F	3.574454	1.740598	-1.793943
F	5.549712	1.050121	-1.194469
F	4.097419	1.665333	0.309410
C	-3.570708	-0.563076	1.662924
C	-2.888117	0.668508	1.731384
C	-1.553164	0.682705	2.085473
C	-0.863793	-0.521479	2.364302
C	-1.575978	-1.759982	2.429360
C	-2.942082	-1.742835	2.041469
H	-4.615644	-0.580948	1.368687
H	-1.170455	-2.623262	2.956939
H	-3.506760	-2.669916	2.074507
C	-0.713423	1.887939	2.373477
O	0.412837	1.586786	2.961274
O	-1.046336	3.040296	2.047507
C	0.564637	-0.376397	2.604082
H	1.232767	-0.004907	1.844573
H	1.024722	-0.870680	3.458099
H	0.279096	0.221139	-1.155592
K	1.312575	2.866927	0.369453
H	-3.401124	1.605203	1.540019

11-ts-Cs

Pd	-0.377092	2.103150	-1.045567
C	-1.336009	-0.434519	1.818650
C	-2.667650	-0.115185	2.186302
C	-3.568043	-1.150110	2.396504
C	-3.144185	-2.470198	2.219366
C	-1.832781	-2.680499	1.808384
N	-0.938501	-1.696623	1.614589
H	-4.590337	-0.926113	2.681089
H	-3.820025	-3.304504	2.367725
H	-1.475987	-3.690546	1.619563
O	-0.473203	0.560160	1.686596
C	-3.078810	1.320963	2.303801
F	-2.383860	1.988105	3.255022
F	-4.391743	1.441546	2.622828
F	-2.900070	2.005048	1.144397
C	2.274357	1.302079	0.241272
C	3.678842	1.424869	0.500572
C	4.309957	2.654608	0.418497
C	3.563177	3.786487	0.072738
C	2.214384	3.613764	-0.204354
N	1.579181	2.426107	-0.141455
H	5.372611	2.732758	0.624195
H	4.016399	4.768518	0.006916
H	1.599516	4.459663	-0.498356
O	1.672002	0.178521	0.350442
C	4.433970	0.181301	0.839955
F	3.953527	-0.458261	1.935992
F	5.744878	0.425017	1.079004
F	4.396192	-0.737056	-0.171178
C	-4.004068	0.639926	-1.585700
C	-3.373188	-0.627509	-1.498404
C	-2.028624	-0.706324	-1.855585
C	-1.329579	0.446971	-2.290277
C	-2.005890	1.680269	-2.516693
C	-3.363413	1.747539	-2.114585
H	-5.047147	0.719815	-1.290529
H	-1.584311	2.454437	-3.158695
H	-3.912433	2.673945	-2.256938
C	-1.186377	-1.946552	-2.039058
O	-0.086841	-1.705889	-2.698556
O	-1.513407	-3.064498	-1.612651
C	0.103073	0.278734	-2.491720
H	0.761623	-0.026567	-1.691958
H	0.575754	0.656957	-3.395788
H	0.359851	0.306910	1.155090
C	-4.192854	-1.833296	-1.114271
H	-3.588313	-2.577818	-0.601225
H	-5.037648	-1.540041	-0.483892
H	-4.602440	-2.311776	-2.013821
Cs	1.754144	-2.884596	-0.153688

11-ts-Cs-rea

Pd	0.488959	-1.388344	-0.182991
C	1.086106	1.243732	1.602195
C	2.463019	1.200344	1.923736
C	3.197873	2.376702	1.833955
C	2.558569	3.554832	1.442303
C	1.192787	3.497329	1.184160
N	0.465627	2.372089	1.256835
H	4.255950	2.370477	2.070797
H	3.101795	4.488980	1.356916
H	0.648920	4.397061	0.902529
O	0.377791	0.107893	1.654035
C	3.087754	-0.064499	2.433485
F	2.564054	-0.435766	3.627681
F	4.422771	0.073993	2.615319
F	2.921805	-1.121418	1.601956
C	-2.396925	-1.040367	0.702580
C	-3.790554	-1.374339	0.731175
C	-4.212518	-2.664265	0.465755
C	-3.264953	-3.648401	0.156577
C	-1.935178	-3.269713	0.084353

N	-1.502275	-2.013190	0.325109
H	-5.269844	-2.905115	0.500490
H	-3.553921	-4.673078	-0.044061
H	-1.169744	-3.988473	-0.186127
O	-1.994539	0.137682	0.998913
C	-4.764085	-0.279305	1.035140
F	-4.576207	0.282667	2.250059
F	-6.046079	-0.717236	1.000638
F	-4.685967	0.738774	0.128625
C	4.583973	-0.930849	-1.035127
C	3.594914	-0.065081	-1.489359
C	2.330035	-0.598846	-1.862277
C	2.097816	-2.007555	-1.725928
C	3.131082	-2.846952	-1.216141
C	4.362355	-2.315351	-0.901309
H	5.556444	-0.524826	-0.767171
H	2.929604	-3.907788	-1.095644
H	5.158442	-2.954710	-0.531140
C	1.271967	0.321390	-2.512225
O	0.902283	0.016638	-3.665444
O	0.896728	1.288050	-1.790690
C	0.742652	-2.502383	-1.882240
H	0.124486	-2.045395	-2.652853
H	0.586421	-3.572047	-1.743414
H	-0.649502	0.235999	1.411000
C	3.869981	1.413109	-1.615176
H	3.129454	1.985976	-1.054483
H	4.873542	1.658297	-1.255660
H	3.792292	1.737960	-2.660025
Cs	-1.942753	2.313815	-1.302436

11-ts-H

Pd	-0.672202	-1.186856	-0.673130
C	-0.230073	2.066963	-0.598134
C	0.474901	2.864075	0.369051
C	-0.201850	3.767205	1.160706
C	-1.599506	3.933817	1.053568
C	-2.273580	3.157660	0.147143
N	-1.595568	2.276705	-0.629347
H	0.353328	4.354739	1.884309
H	-2.131728	4.641538	1.675445
H	-3.346431	3.186541	0.000904
O	0.259138	1.222412	-1.390763
C	1.948228	2.631278	0.540504
F	2.187365	1.413780	1.095917
F	2.510481	3.547681	1.357901
F	2.618176	2.667623	-0.627762
C	2.426663	-1.053202	-0.260670
C	3.689971	-1.378385	0.281123
C	3.763586	-2.255054	1.352212
C	2.582632	-2.786875	1.877501
C	1.379861	-2.403717	1.308729
N	1.289809	-1.559609	0.254667
H	4.727382	-2.505416	1.780952
H	2.594890	-3.472758	2.716249
H	0.440086	-2.779784	1.695736
O	2.387485	-0.243354	-1.308909
C	4.913837	-0.700849	-0.267579
F	5.105290	-0.958073	-1.580288
F	6.033356	-1.105322	0.379281
F	4.844360	0.643389	-0.133304
C	-4.091557	0.493166	-1.775994
C	-4.462642	0.285981	-0.418818
C	-3.860713	-0.770467	0.258938
C	-2.893408	-1.584733	-0.379251
C	-2.610605	-1.445122	-1.767808
C	-3.227671	-0.362080	-2.444811
H	-4.567110	1.301695	-2.325896
H	-2.134426	-2.242988	-2.338093
H	-3.045078	-0.227052	-3.506633
C	-4.300744	-1.374912	1.579746
O	-3.792980	-2.554442	1.775017
O	-5.098048	-0.798837	2.330474
H	-2.114972	1.680518	-1.273237
C	-2.223511	-2.555943	0.470653
H	-1.732479	-2.273200	1.390202
H	-2.080517	-3.580504	0.136496
H	1.557007	0.318775	-1.330716
C	-5.512049	1.161649	0.216820
H	-6.338053	0.558208	0.602205
H	-5.107429	1.692099	1.083888
H	-5.899520	1.892631	-0.499114

11-ts-H-rea

Pd	-0.868148	-1.134842	0.007981
C	-0.416911	2.059196	0.275374
C	0.349198	3.263418	0.161082
C	-0.066451	4.416651	0.796533
C	-1.242088	4.423956	1.569296
C	-1.941290	3.245407	1.684254
N	-1.535642	2.117734	1.056582
H	0.525913	5.320033	0.697981
H	-1.581980	5.319582	2.073345
H	-2.844671	3.142149	2.275264
O	-0.112086	0.972650	-0.319471
C	1.628956	3.216742	-0.621562
F	2.536695	2.394147	-0.040956
F	2.204219	4.435562	-0.718469
F	1.444264	2.761756	-1.882150
C	2.222992	-1.183225	-0.009413
C	3.527306	-1.605975	0.327561
C	3.693734	-2.588284	1.291679
C	2.567363	-3.130836	1.917063
C	1.319114	-2.663568	1.544861
N	1.142125	-1.716928	0.593728
H	4.691495	-2.915753	1.561046
H	2.656985	-3.895256	2.679609
H	0.414947	-3.047098	2.002705
O	2.082423	-0.265063	-0.955282
C	4.702105	-0.942998	-0.337135
F	4.696906	-1.116853	-1.677096
F	5.872971	-1.443173	0.122399
F	4.722988	0.389036	-0.109691
C	-4.093613	-0.334010	-2.330465
C	-3.992577	-0.049974	-0.974389
C	-3.327880	-0.986375	-0.125275

C	-2.760516	-2.180921	-0.701893
C	-2.854183	-2.395994	-2.107960
C	-3.533431	-1.496753	-2.898511
H	-4.626979	0.360874	-2.974184
H	-2.392464	-3.283050	-2.531311
H	-3.631368	-1.671615	-3.965818
C	-3.461711	-0.852686	1.405525
O	-4.035672	-1.776422	1.993348
O	-2.983200	0.212371	1.931993
H	-2.126925	1.212675	1.276376
C	-1.852628	-2.951172	0.120999
H	-2.100558	-3.088915	1.171982
H	-1.338255	-3.789589	-0.346371
H	1.261613	0.285733	-0.812788
C	-4.608474	1.214554	-0.424770
H	-5.062670	1.058053	0.556580
H	-3.851241	1.996969	-0.304568
H	-5.372584	1.596834	-1.107314

11-ts-Li

Pd	1.530150	-2.110791	-0.896985
C	-2.176915	1.166209	-0.251875
C	-3.550874	1.191949	-0.593332
C	-4.378542	2.113013	0.031884
C	-3.842063	2.988584	0.981112
C	-2.491711	2.875529	1.276632
N	-1.671078	1.985254	0.685418
H	-5.433507	2.142584	-0.217366
H	-4.459040	3.723091	1.484947
H	-2.031314	3.513584	2.027160
O	-1.391335	0.293597	-0.864572
C	-4.088566	0.188800	-1.569901
F	-3.485836	0.258673	-2.776754
F	-5.415109	0.357275	-1.780499
F	-3.923532	-1.084516	-1.124200
C	2.066344	0.828199	-0.645186
C	2.821868	2.036773	-0.580664
C	4.000212	2.168892	-1.298742
C	4.437424	1.099355	-2.086680
C	3.670066	-0.058178	-2.104002
N	2.526725	-0.210910	-1.405959
H	4.567135	3.092680	-1.247139
H	5.350050	1.160475	-2.667446
H	3.975721	-0.916397	-2.695041
O	0.978785	0.689119	0.029572
C	2.270180	3.155227	0.240239
F	1.096832	3.635969	-0.243047
F	3.114762	4.192881	0.351968
F	1.972607	2.761707	1.538044
C	-1.766876	-3.017371	0.561763
C	-1.461395	-1.951803	1.453339
C	-0.123498	-1.721978	1.741987
C	0.895808	-2.528410	1.159641
C	0.580330	-3.669369	0.355713
C	-0.796820	-3.864185	0.055381
H	-2.809793	-3.195466	0.315358
H	1.301167	-4.468231	0.190114
H	-1.087722	-4.713650	-0.555818
C	0.500185	-0.746241	2.698198
O	1.660113	-1.113171	3.116062
O	-0.039368	0.354105	2.995682
C	2.250545	-2.134021	1.491027
H	2.647390	-1.157920	1.259854
H	2.988580	-2.896706	1.734762
H	-0.431970	0.375935	-0.559314
C	-2.597325	-1.192386	2.089838
H	-2.261319	-0.253570	2.527248
H	-3.375801	-0.991498	1.348943
H	-3.055151	-1.795735	2.884253
Li	0.175277	1.532728	1.510927

11-ts-Li-rea

Pd	-0.098351	-0.405240	-1.447814
C	-1.456370	1.579983	0.621530
C	-2.842327	1.703462	0.398615
C	-3.700110	1.579237	1.484376
C	-3.169573	1.333321	2.752899
C	-1.793403	1.190598	2.869521
N	-0.945691	1.296256	1.827926
H	-4.769997	1.675515	1.336971
H	-3.808765	1.238551	3.622593
H	-1.336234	0.966904	3.829212
O	-0.615295	1.735666	-0.411143
C	-3.354151	1.962378	-0.988457
F	-2.900605	3.129206	-1.494708
F	-4.704525	2.008499	-1.021032
F	-2.975817	0.989112	-1.858993
C	2.490458	0.726572	-0.458503
C	3.886119	0.640799	-0.201721
C	4.730603	0.065097	-1.135698
C	4.193129	-0.438533	-2.326511
C	2.820223	-0.386106	-2.498274
N	1.981770	0.162589	-1.590282
H	5.795238	0.004149	-0.937130
H	4.822029	-0.886851	-3.086240
H	2.349200	-0.806809	-3.380221
O	1.694946	1.308189	0.386293
C	4.388244	1.167778	1.108081
F	4.210501	2.501127	1.242340
F	5.705855	0.924525	1.278604
F	3.745657	0.589192	2.166645
C	-3.247821	-1.957058	0.795012
C	-2.013805	-2.179966	1.439094
C	-0.863737	-2.359031	0.664009
C	-0.970284	-2.380247	-0.769296
C	-2.231686	-2.119822	-1.379690
C	-3.361591	-1.902945	-0.590595
H	-4.133246	-1.808796	1.407388
H	-2.312469	-2.154068	-2.462571
H	-4.321143	-1.702853	-1.055911
C	0.513286	-2.510251	1.314400
O	1.102402	-3.587731	1.171004
O	0.929402	-1.474319	1.943052
C	0.200747	-2.417931	-1.626304
H	1.145150	-2.740041	-1.196291
H	0.053627	-2.706266	-2.668066

H	0.377922	1.655817	-0.078242
C	-1.945452	-2.235945	2.946249
H	-1.162187	-1.576241	3.325194
H	-2.899242	-1.955955	3.400537
H	-1.695413	-3.250269	3.280195
Li	0.970299	0.326201	1.852239

11-ts-Na

Pd	0.891461	-2.285023	-0.880479
C	-1.813281	1.554444	-0.262356
C	-3.106979	1.759645	-0.804338
C	-3.865453	2.825471	-0.342883
C	-3.338515	3.670068	0.638571
C	-2.069363	3.386732	1.122057
N	-1.315656	2.355160	0.693725
H	-4.858616	2.992135	-0.745030
H	-3.901065	4.513884	1.020308
H	-1.623405	4.007109	1.896405
O	-1.101275	0.535094	-0.715847
C	-3.648290	0.798235	-1.819955
F	-2.861185	0.693150	-2.913565
F	-4.877151	1.168517	-2.253258
F	-3.776952	-0.453674	-1.310153
C	2.246035	0.381179	-0.631316
C	3.274109	1.368913	-0.721916
C	4.281468	1.252160	-1.666690
C	4.282557	0.150287	-2.528156
C	3.274454	-0.793761	-2.382999
N	2.288827	-0.703085	-1.468290
H	5.054835	2.010610	-1.731559
H	5.048630	0.023128	-3.283782
H	3.244079	-1.673704	-3.018855
O	1.297668	0.479858	0.230822
C	3.217971	2.516605	0.231290
F	2.062950	3.239484	0.127294
F	4.235345	3.382130	0.073482
F	3.252036	2.109678	1.544357
C	-2.553507	-2.606719	0.523012
C	-2.070026	-1.662307	1.470785
C	-0.721536	-1.713671	1.798863
C	0.140755	-2.653477	1.166422
C	-0.365828	-3.669133	0.297653
C	-1.751725	-3.595993	-0.015586
H	-3.605686	-2.570987	0.256296
H	0.202147	-4.573751	0.085261
H	-2.185405	-4.347738	-0.668800
C	0.034857	-0.966035	2.864590
O	1.140112	-1.540016	3.200989
O	-0.353941	0.131371	3.338058
C	1.546560	-2.508170	1.484964
H	2.076669	-1.584072	1.317003
H	2.156951	-3.390177	1.670643
H	-0.171968	0.484353	-0.318330
C	-3.051300	-0.700533	2.090453
H	-2.547175	0.068622	2.673299
H	-3.648301	-0.229106	1.303253
H	-3.749886	-1.235424	2.746228
Na	0.623191	1.653089	1.981935

11-ts-Na-rea

Pd	-0.138525	-0.085711	-1.475509
C	-1.530770	1.383447	0.955569
C	-2.898941	1.620555	0.702147
C	-3.823332	1.285097	1.683172
C	-3.376219	0.722885	2.881035
C	-2.012152	0.511350	3.032909
N	-1.098898	0.822838	2.092631
H	-4.878949	1.462823	1.510953
H	-4.067871	0.450308	3.669340
H	-1.620781	0.062472	3.942067
O	-0.637238	1.719184	0.015669
C	-3.322879	2.239288	-0.598657
F	-2.796350	3.470963	-0.774255
F	-4.667314	2.364881	-0.676047
F	-2.940505	1.498177	-1.671537
C	2.463922	0.898171	-0.345020
C	3.871202	0.768422	-0.162324
C	4.683802	0.423415	-1.228548
C	4.105915	0.188038	-2.482065
C	2.726540	0.249690	-2.586716
N	1.917524	0.574119	-1.553353
H	5.754977	0.330846	-1.084375
H	4.708642	-0.072658	-3.343650
H	2.225192	0.016076	-3.519866
O	1.704838	1.276553	0.631930
C	4.416995	0.969289	1.216716
F	4.204163	2.208349	1.705612
F	5.743733	0.736857	1.278763
F	3.835795	0.104335	2.118057
C	-3.252369	-2.028974	0.295549
C	-2.042604	-2.523735	0.829515
C	-0.904767	-2.570101	0.019381
C	-0.998560	-2.155552	-1.354257
C	-2.228914	-1.622196	-1.843399
C	-3.350368	-1.565241	-1.009735
H	-4.128534	-1.998184	0.937643
H	-2.308809	-1.345401	-2.891131
H	-4.287266	-1.167117	-1.384870
C	0.450539	-3.024969	0.571214
O	0.992607	-3.995684	0.020891
O	0.890933	-2.330529	1.546194
C	0.168829	-1.978523	-2.196976
H	1.110719	-2.411101	-1.872756
H	0.016697	-1.967975	-3.277025
H	0.368907	1.587405	0.336638
C	-1.989872	-2.976349	2.268954
H	-1.332230	-2.327432	2.852759
H	-2.984971	-2.971631	2.722153
H	-1.580791	-3.989391	2.347621
Na	1.153275	-0.289756	2.156308

11-tsp

C	3.077530	-3.064700	0.283897
C	3.471387	-1.697432	0.146612
C	2.652125	-0.868286	-0.600053
C	1.444167	-1.370257	-1.193427

C	1.121507	-2.774811	-1.166217
C	1.977335	-3.590444	-0.365197
H	3.719437	-3.726059	0.860830
H	0.486732	-3.234281	-1.919952
H	1.777579	-4.656830	-0.303948
C	2.870732	0.552427	-1.018210
O	2.251805	0.859007	-2.120279
O	3.516890	1.382509	-0.333905
C	0.674297	-0.411275	-1.958917
H	0.213558	0.472851	-1.519641
H	0.296385	-0.710753	-2.938140
C	4.778964	-1.254214	0.759776
H	5.051672	-0.245157	0.449983
H	4.721404	-1.276729	1.857087
H	5.586043	-1.941695	0.482022
K	2.235564	1.100073	2.254859
Pd	-0.245943	-1.536885	0.040160
H	-2.226466	-1.269365	-1.425824
P	-0.498878	1.903861	0.806858
O	-0.146846	0.459484	1.255473
O	0.547911	2.922604	1.301517
O	-0.725338	2.027467	-0.729307
O	-1.948623	2.267930	1.520215
H	-2.491687	1.445839	1.550860
K	1.525321	3.399324	-1.185897
P	-3.423409	-1.141317	0.379091
O	-2.340267	-2.057012	0.921805
O	-3.710832	0.227631	0.955065
O	-3.119477	-0.918142	-1.245668
O	-4.844810	-1.942237	0.356807
H	-4.679118	-2.888111	0.226049
K	-3.264014	1.938965	-1.139452

12

Pd	0.122033	-2.280900	0.417164
C	-1.838117	0.976612	-1.332161
C	-3.201147	0.674781	-1.588773
C	-4.144513	1.685199	-1.479880
C	-3.733575	2.969445	-1.110151
C	-2.383341	3.167531	-0.853994
N	-1.445775	2.206602	-0.960080
H	-5.189754	1.468560	-1.670495
H	-4.442126	3.783569	-1.011149
H	-2.026671	4.146743	-0.540807
O	-0.950069	0.011435	-1.479374
C	-3.598660	-0.732057	-1.924114
F	-2.993669	-1.194282	-3.042069
F	-4.935067	-0.836828	-2.130811
F	-3.292144	-1.600777	-0.928406
C	2.212779	-0.466628	-0.850172
C	3.579530	-0.142212	-1.130820
C	4.470100	-1.123747	-1.540036
C	4.020049	-2.440505	-1.668749
C	2.697236	-2.709420	-1.337547
N	1.817048	-1.777890	-0.921829
H	5.500940	-0.863521	-1.757643
H	4.678422	-3.236535	-1.996066
H	2.311989	-3.723766	-1.391516
O	1.367118	0.441750	-0.519306
C	4.014103	1.271654	-0.952050
F	3.321967	2.154672	-1.736319
F	5.319507	1.461291	-1.224738
F	3.818262	1.719224	0.332287
C	-2.259836	-1.558973	2.037427
C	-2.127086	-0.190271	2.194164
C	-0.817976	0.279056	2.503968
C	0.265345	-0.576158	2.661614
C	0.133818	-1.982500	2.565538
C	-1.163316	-2.471323	2.197388
H	-3.233284	-1.969423	1.785042
H	0.902190	-2.638834	2.966024
H	-1.390754	-3.528856	2.311215
C	-0.319279	1.647133	2.547203
O	1.044877	1.616450	2.771629
O	-0.871371	2.720646	2.359145
C	1.499170	0.243594	2.885986
H	2.265548	0.076671	2.124231
H	1.942417	0.107884	3.879057
H	-0.033285	0.220997	-1.071298
C	-3.286591	0.755796	2.035753
H	-3.042919	1.570867	1.350351
H	-4.167725	0.230414	1.657830
H	-3.551768	1.222764	2.992607
K	1.119896	2.933627	0.142454

12b-bz

Pd	-0.258756	-1.908080	0.915778
C	-1.895234	0.665813	-1.402141
C	-3.272140	0.327472	-1.405559
C	-4.208824	1.329914	-1.198119
C	-3.772571	2.640081	-0.980324
C	-2.403371	2.875272	-0.974310
N	-1.476351	1.922817	-1.186306
H	-5.265782	1.087391	-1.194243
H	-4.474269	3.448428	-0.809982
H	-2.018970	3.871506	-0.773790
O	-1.012139	-0.293673	-1.622308
C	-3.685599	-1.102779	-1.584975
F	-3.236389	-1.634252	-2.744403
F	-5.035355	-1.235015	-1.589717
F	-3.225323	-1.902597	-0.587988
C	2.084798	-0.820401	-0.696814
C	3.494275	-0.719643	-0.929324
C	4.295818	-1.851332	-0.907395
C	3.711261	-3.094419	-0.648400
C	2.348043	-3.127606	-0.378738
N	1.551223	-2.041260	-0.376196
H	5.361818	-1.764750	-1.091692
H	4.297620	-4.005597	-0.633988
H	1.857435	-4.066373	-0.136992
O	1.324883	0.212857	-0.764674
C	4.067812	0.636486	-1.160550
F	3.522368	1.281636	-2.235165
F	5.400098	0.619087	-1.358853
F	3.846499	1.480731	-0.098085
C	-2.494692	-0.423530	2.200282

C	-2.137292	0.895312	2.012656
C	-0.781619	1.242910	2.226445
C	0.166161	0.311788	2.624736
C	-0.180002	-1.037930	2.891639
C	-1.542611	-1.412491	2.627820
H	-3.519005	-0.737900	2.025834
H	0.480502	-1.668381	3.481832
H	-1.922432	-2.355473	3.014684
C	-0.097528	2.495047	1.936128
O	1.251807	2.325545	2.174378
O	-0.505301	3.553685	1.483302
C	1.508318	0.978209	2.652042
H	2.229875	0.512529	1.975140
H	1.947392	1.045884	3.653845
H	-0.075758	-0.056410	-1.274626
K	1.272624	2.785603	-0.798547
H	-2.850731	1.640838	1.679788

12b-o

Pd	-0.330444	-1.961432	0.052231
C	1.311148	1.726599	0.694692
C	2.638019	1.792796	1.191954
C	3.542481	2.647575	0.579531
C	3.131576	3.406582	-0.519964
C	1.828177	3.239788	-0.968553
N	0.928915	2.422144	-0.389958
H	4.559458	2.710894	0.950401
H	3.809165	4.086127	-1.023595
H	1.479803	3.784088	-1.843585
O	0.447300	0.955704	1.329037
C	3.044886	0.911763	2.334105
F	2.384636	1.193693	3.479906
F	4.370694	1.031278	2.604999
F	2.826532	-0.400068	2.070524
C	-2.639502	-0.118340	0.643376
C	-4.040808	0.159227	0.673400
C	-4.923925	-0.754003	1.227596
C	-4.426575	-1.953195	1.749805
C	-3.063322	-2.197008	1.638203
N	-2.189599	-1.330958	1.087461
H	-5.986608	-0.535185	1.251652
H	-5.080824	-2.688369	2.203353
H	-2.637904	-3.132631	1.989912
O	-1.805769	0.738850	0.174304
C	-4.504036	1.436257	0.052819
F	-3.982463	2.541842	0.630606
F	-5.848701	1.568950	0.088812
F	-4.152564	1.515568	-1.276994
C	3.207624	-2.651458	-0.310671
C	3.284642	-1.337971	-0.858702
C	2.126504	-0.922584	-1.478898
C	0.951313	-1.719623	-1.604497
C	0.945117	-3.098195	-1.275029
C	2.115903	-3.483927	-0.526668
H	4.057215	-3.032420	0.248822
H	0.300618	-3.849042	-1.721373
H	2.160274	-4.505095	-0.157453
C	1.573165	-0.058280	-2.531153
O	0.415360	-0.886684	-2.686300
O	1.782908	0.942377	-3.165500
H	-0.450633	0.877021	0.844345
K	-1.251092	1.565060	-2.153106
C	4.542392	-0.522209	-0.785443
H	5.392841	-1.081151	-1.191407
H	4.443972	0.417400	-1.333464
H	4.778312	-0.286342	0.257806

PdL1P

Pd	-0.611030	-0.370962	0.146931
C	1.887627	-0.139902	0.005763
C	3.295387	-0.134228	-0.140966
C	3.903958	-1.269897	-0.661330
C	3.141897	-2.387930	-1.030530
C	1.763554	-2.337121	-0.865184
N	1.178822	-1.242655	-0.362217
H	4.982196	-1.283383	-0.780651
H	3.610848	-3.275823	-1.436802
H	1.110834	-3.162677	-1.129525
O	1.127389	0.807435	0.462783
C	4.068158	1.079618	0.267274
F	3.682331	2.183149	-0.417949
F	5.396340	0.926734	0.055931
F	3.906803	1.366793	1.580885
P	-3.289349	-0.551203	0.275905
O	-2.219254	-1.599220	-0.229090
O	-2.320216	0.651537	0.686080
O	-4.388468	-0.113237	-0.651836
O	-3.975757	-1.157880	1.619199
H	-3.302989	-1.571644	2.182447
K	-3.590912	2.392735	-0.984969

3-LP-a

Pd	0.132177	0.030794	-0.486326
C	2.364754	-1.067707	-0.163571
C	3.541879	-1.783170	0.161116
C	3.415462	-2.938033	0.925459
C	2.158731	-3.380427	1.364022
C	1.033005	-2.637168	1.028020
N	1.166106	-1.526416	0.292898
H	4.308398	-3.499387	1.180260
H	2.059885	-4.284418	1.952957
H	0.015568	-2.905380	1.301010
O	2.244712	0.018091	-0.864283
C	4.863442	-1.272860	-0.316850
F	4.921135	-1.190364	-1.668195
F	5.884837	-2.071409	0.073823
F	5.129853	-0.030996	0.155387
C	-0.097203	3.398708	2.755772
C	-0.866398	3.055416	1.638211
C	-0.249570	3.066278	0.368903
C	1.102769	3.433191	0.214322
C	1.832596	3.789863	1.355864
C	1.242241	3.765686	2.618448
H	-0.556685	3.381599	3.740862
H	2.873165	4.085331	1.247970
H	1.823414	4.038676	3.495290

C	-1.058039	2.672277	-0.843460
O	-2.064287	3.320057	-1.174523
O	-0.665506	1.628661	-1.519840
C	1.775401	3.430178	-1.139186
H	2.058747	2.412298	-1.424325
H	1.121174	3.821109	-1.924806
H	2.683533	4.040112	-1.119904
C	-2.310288	2.640468	1.803462
H	-2.969875	3.264911	1.193752
H	-2.446232	1.600887	1.482390
H	-2.621932	2.721269	2.849010
K	-3.431898	0.994610	-1.830044
P	-2.549891	-1.594953	-0.213194
O	-1.764043	-0.260557	0.152166
O	-2.173395	-2.794767	0.644821
O	-4.042321	-1.252268	-0.267833
O	-2.154543	-1.876813	-1.806581
H	-1.213571	-2.096267	-1.887819
K	-4.678111	-3.155061	1.432583

3-LP-b

Pd	-0.214327	-0.292308	-0.429104
C	-2.966524	0.835452	-0.399567
C	-3.697544	-0.127566	0.370156
C	-4.770660	0.292997	1.150161
C	-5.135445	1.638627	1.173908
C	-4.392602	2.513045	0.388849
N	-3.349456	2.139278	-0.361826
H	-5.328346	-0.435756	1.728508
H	-5.969087	1.990744	1.770611
H	-4.640088	3.573239	0.362178
O	-1.966227	0.546643	-1.194262
C	-3.362441	-1.588770	0.342836
F	-3.117795	-2.056516	-0.889305
F	-4.356207	-2.348158	0.865267
F	-2.251477	-1.883313	1.107714
C	3.992110	0.448305	2.169204
C	2.695732	0.913576	1.894273
C	2.478543	1.638069	0.704183
C	3.525073	1.853642	-0.221209
C	4.801707	1.354919	0.076559
C	5.041158	0.672644	1.275961
H	4.175602	-0.090357	3.095205
H	5.616068	1.524257	-0.623917
H	6.042752	0.321375	1.511263
C	1.135003	2.279066	0.445432
O	1.089677	3.506222	0.240300
O	0.055534	1.559387	0.466422
C	3.287687	2.582991	-1.524017
H	3.030322	3.629657	-1.342985
H	4.175699	2.544117	-2.161256
H	2.453736	2.137096	-2.080497
C	1.576461	0.610892	2.859561
H	0.908584	1.464577	3.003236
H	0.972237	-0.212953	2.466273
H	1.974884	0.313856	3.833975
P	0.819958	-2.757765	-0.496561
O	-0.331038	-2.131918	-1.368249
O	1.940542	-3.508634	-1.163373
O	1.350326	-1.452794	0.263257
O	0.102477	-3.739261	0.588003
H	-0.767664	-3.371935	0.811832
K	3.856074	-1.715133	-0.644895
K	-1.081832	3.257088	-1.423634

4-ts-LP-a

Pd	-0.045611	0.059267	-0.360894
C	2.651084	-0.998857	0.002486
C	3.652411	-1.980780	0.274434
C	3.297650	-3.305189	0.459030
C	1.952101	-3.686640	0.379111
C	1.003761	-2.710886	0.125647
N	1.343007	-1.415487	-0.051225
H	4.068235	-4.041677	0.660513
H	1.646544	-4.717790	0.509747
H	-0.054850	-2.940668	0.055102
O	2.920196	0.234028	-0.195832
C	5.079174	-1.531839	0.338399
F	5.499061	-0.976390	-0.824036
F	5.918326	-2.565370	0.600141
F	5.289099	-0.607007	1.305695
C	0.839452	5.314754	0.777314
C	-0.360586	4.638580	0.518138
C	-0.320475	3.437061	-0.238069
C	0.922501	2.945003	-0.719127
C	2.094010	3.663884	-0.443292
C	2.056956	4.839595	0.298384
H	0.812023	6.226836	1.367140
H	3.042687	3.285456	-0.813166
H	2.975364	5.381170	0.507735
C	-1.605668	2.694405	-0.499870
O	-2.705329	3.270239	-0.475069
O	-1.572715	1.400991	-0.712178
C	1.079441	1.665220	-1.492042
H	0.292005	1.481164	-2.225560
H	2.007462	1.683048	-2.075578
H	1.778130	0.802760	-0.736572
C	-1.629237	5.229957	1.091965
H	-2.289864	5.600090	0.304288
H	-2.207872	4.488511	1.651341
H	-1.384849	6.057540	1.764605
K	-3.702873	1.011227	1.070670
P	-2.594152	-1.780928	-0.287860
O	-1.497802	-1.031944	0.590123
O	-2.305601	-3.259705	-0.505420
O	-3.973727	-1.496854	0.349540
O	-2.552025	-1.058046	-1.773262
H	-2.438616	-0.095694	-1.669516
K	-4.601068	-4.049706	0.444227

4-ts-LP-b

Pd	0.061133	-0.179829	-0.868377
C	-2.664050	-1.258180	-0.229671
C	-2.592596	-1.831783	1.079249
C	-3.710640	-1.812103	1.904718
C	-4.894891	-1.229094	1.452390

C	-4.890701	-0.682517	0.173500
N	-3.824979	-0.680588	-0.638647
H	-3.657573	-2.255540	2.893236
H	-5.785339	-1.200311	2.069891
H	-5.788793	-0.210629	-0.222619
O	-1.683123	-1.304655	-1.090069
C	-1.331137	-2.471288	1.563988
F	-0.852364	-3.407545	0.692706
F	-1.493615	-3.104170	2.747310
F	-0.315965	-1.583199	1.745810
C	1.301989	4.501650	2.002860
C	0.171180	4.130661	1.264636
C	0.297291	3.099098	0.307655
C	1.542587	2.472652	0.081557
C	2.654565	2.885708	0.828897
C	2.536460	3.889212	1.786171
H	1.209721	5.280306	2.755303
H	3.617983	2.415321	0.648927
H	3.404213	4.195634	2.364216
C	-0.918721	2.664795	-0.466730
O	-1.665633	3.488397	-1.014175
O	-1.203964	1.386842	-0.500886
C	1.712998	1.383710	-0.933393
H	1.158343	1.574405	-1.860268
H	2.753120	1.301575	-1.268377
H	1.917449	0.262231	-0.274404
C	-1.147630	4.820170	1.526874
H	-1.450944	5.426612	0.669077
H	-1.953000	4.097544	1.696712
H	-1.076758	5.465905	2.406701
P	2.475765	-2.019524	-0.505550
O	1.224046	-1.761295	-1.432117
O	3.758077	-2.395283	-1.223941
O	2.669160	-0.747137	0.404262
O	2.067281	-3.282481	0.451520
H	1.108399	-3.282630	0.588064
K	5.324010	-0.793153	0.084635
K	-2.924012	1.143767	-2.503199

5-LP

Pd	0.114439	0.157657	0.361411
C	-2.507182	-1.236086	-0.121218
C	-3.431678	-2.296474	-0.142018
C	-2.990405	-3.568305	0.198191
C	-1.649547	-3.764269	0.537688
C	-0.796572	-2.672832	0.547719
N	-1.229540	-1.424832	0.248017
H	-3.688554	-4.397566	0.186750
H	-1.269598	-4.747382	0.788415
H	0.261516	-2.774328	0.771796
O	-2.905597	-0.016583	-0.485414
C	-4.856486	-2.030350	-0.536628
F	-5.441563	-1.118216	0.272868
F	-5.604922	-3.154426	-0.476048
F	-4.953554	-1.554683	-1.798562
C	-1.619056	5.146193	-0.472351
C	-0.295669	4.711210	-0.295738
C	-0.064423	3.476230	0.357642
C	-1.162040	2.712900	0.849299
C	-2.466793	3.191697	0.650505
C	-2.695933	4.396299	-0.008748
H	-1.799019	6.085154	-0.988874
H	-3.304693	2.608718	1.023656
H	-3.712366	4.750676	-0.158306
C	1.346217	2.943102	0.464094
O	2.332181	3.692919	0.437780
O	1.524296	1.643574	0.515486
C	-0.980810	1.408951	1.556114
H	-0.303696	1.488320	2.414151
H	-1.933106	0.989803	1.891503
H	-2.132914	0.586240	-0.425595
C	0.808397	5.586392	-0.845683
H	1.428337	5.994555	-0.043697
H	1.487493	5.025173	-1.494285
H	0.382231	6.415447	-1.418875
K	3.416225	1.192143	-1.393726
P	2.789990	-1.562966	0.131977
O	1.635485	-0.948625	-0.737056
O	2.646686	-3.053089	0.436709
O	4.158116	-1.180147	-0.492202
O	2.708245	-0.805509	1.615759
H	2.550293	0.146093	1.492289
K	5.052392	-3.626205	-0.349729

6-LP

Pd	0.517845	-0.480150	-0.659625
C	-2.239700	-1.409269	0.158312
C	-3.396461	-2.236132	0.160137
C	-3.515019	-3.265694	-0.756336
C	-2.488220	-3.477491	-1.681734
C	-1.376318	-2.658232	-1.623256
N	-1.226141	-1.664469	-0.716328
H	-4.403246	-3.887536	-0.758614
H	-2.548872	-4.259745	-2.428809
H	-0.558951	-2.788987	-2.320756
O	-2.167023	-0.424351	1.008386
C	-4.486959	-1.944700	1.149936
F	-4.055912	-2.026597	2.430208
F	-5.513124	-2.822638	1.028066
F	-5.012485	-0.708043	0.991767
C	5.093986	-2.420216	1.322781
C	4.822572	-1.105267	0.914432
C	3.739427	-0.874982	0.028589
C	2.964432	-1.970122	-0.450522
C	3.273676	-3.265087	-0.006254
C	4.327105	-3.491426	0.873897
H	5.914878	-2.597425	2.012230
H	2.680640	-4.100521	-0.369012
H	4.551343	-4.500870	1.208390
C	3.363176	0.541151	-0.334313
O	4.165145	1.482815	-0.208764
O	2.135473	0.791330	-0.713279
C	1.840926	-1.814888	-1.424087
H	2.154370	-1.320790	-2.351703
H	1.392235	-2.782714	-1.657280
H	-1.480232	0.366751	0.708483

C	5.700437	-0.009622	1.477649
H	6.268298	0.492876	0.691153
H	5.112066	0.771016	1.969385
H	6.400910	-0.428459	2.206590
K	1.983510	2.844517	1.154885
P	-0.866502	2.548328	-0.549903
O	-0.551279	1.233282	0.306018
O	-2.328939	2.711906	-0.914476
O	-0.253996	3.754868	0.196151
O	-0.019924	2.331513	-1.948118
H	0.866955	1.978884	-1.753044
K	-2.558830	5.047442	0.199615

11-ts-LP

C	4.440904	-1.427403	1.421376
C	4.159018	-0.213542	0.743637
C	3.251591	-0.265575	-0.311098
C	2.607416	-1.477487	-0.663387
C	3.011473	-2.716411	-0.083013
C	3.929138	-2.643519	0.997748
H	5.143135	-1.406541	2.250479
H	2.813591	-3.670735	-0.570588
H	4.260800	-3.563113	1.471095
C	2.984170	0.798158	-1.330322
O	2.288253	0.369541	-2.348962
O	3.403406	1.966004	-1.230625
C	1.543096	-1.366259	-1.649500
H	0.664097	-0.758262	-1.489486
H	1.493025	-2.071346	-2.477310
C	4.860558	1.057275	1.146416
H	5.385682	1.502633	0.296856
H	4.136873	1.806945	1.481769
H	5.573311	0.870144	1.954436
K	-0.947985	5.074675	2.198834
Pd	0.869352	-2.462721	0.386624
P	-0.760340	2.525678	0.430262
O	0.310277	1.295254	0.535263
O	0.038658	3.789383	0.115358
O	-1.559494	2.151418	-0.955273
O	-1.674454	2.604501	1.633703
K	1.017543	2.852944	-2.195601
C	-1.997964	-1.487378	-0.027226
C	-3.428853	-1.603388	-0.062663
C	-4.063236	-2.717407	0.456662
C	-3.299864	-3.746194	1.021704
C	-1.921989	-3.597476	1.027042
N	-1.279307	-2.522192	0.526136
H	-5.145697	-2.785336	0.423472
H	-3.760012	-4.632992	1.441029
H	-1.286241	-4.370150	1.450222
O	-1.384681	-0.473014	-0.496295
C	-4.207015	-0.479798	-0.668799
F	-4.026839	0.693338	-0.010900
F	-5.541331	-0.722746	-0.668873
F	-3.857635	-0.242105	-1.961319
H	-1.824623	1.200953	-0.922497
H	-0.159620	0.445708	0.340509

3-1L

Pd	-0.353820	-0.139330	0.000257
C	2.153512	-0.129296	0.000199
C	3.556639	0.036810	0.000015
C	4.057355	1.332440	-0.000932
C	3.194047	2.439730	-0.001540
C	1.824788	2.218138	-0.001182
N	1.349772	0.965510	-0.000265
H	5.131579	1.482394	-0.001152
H	3.582115	3.450869	-0.002286
H	1.096223	3.021321	-0.001563
O	1.470780	-1.238721	0.000766
C	4.438614	-1.174125	0.000267
F	4.221531	-1.953560	1.085426
F	5.748116	-0.841225	0.001604
F	4.223557	-1.952454	-1.086187
C	-1.820259	2.886636	1.218613
C	-2.200527	1.544846	1.252080
C	-2.357214	0.856569	0.000279
C	-2.200666	1.544594	-1.251672
C	-1.820353	2.886400	-1.218549
C	-1.639606	3.545894	-0.000049
H	-1.681097	3.421927	2.152560
H	-1.681251	3.421498	-2.152614
H	-1.353352	4.593636	-0.000172
C	-2.920905	-0.559679	0.000459
O	-4.114587	-0.840579	0.000390
O	-1.947737	-1.437185	0.000660
C	-2.519801	0.858036	-2.553493
H	-2.197522	-0.186531	-2.562980
H	-3.604563	0.869465	-2.716878
H	-2.046211	1.372694	-3.392415
C	-2.519270	0.858441	2.554077
H	-3.604063	0.868894	2.717303
H	-2.196075	-0.185850	2.563879
H	-2.046227	1.373780	3.392879
K	-3.628256	-3.572056	-0.001056

4-ts-1L

Pd	-0.284579	-1.093510	0.027767
C	2.491074	-0.123783	-0.021740
C	3.910772	-0.307227	0.151877
C	4.443624	-1.550146	0.415658
C	3.610784	-2.679878	0.519833
C	2.256944	-2.491486	0.344631
N	1.725836	-1.278720	0.084019
H	5.515958	-1.653544	0.542835
H	4.008269	-3.667401	0.716620
H	1.550679	-3.318588	0.395535
O	1.940203	0.979803	-0.249307
C	4.776012	0.913633	0.046334
F	4.676338	1.505703	-1.166457
F	6.084109	0.614562	0.231328
F	4.452670	1.850944	0.966412
C	-3.031805	3.614067	0.577677
C	-3.491038	2.286683	0.562445
C	-2.703565	1.313104	-0.093195
C	-1.499060	1.688260	-0.731440

C	-1.079488	3.023465	-0.702831
C	-1.847922	3.985736	-0.052965
H	-3.617879	4.363128	1.102509
H	-0.148661	3.299655	-1.188705
H	-1.517821	5.019768	-0.028654
C	-3.179937	-0.111930	-0.125758
O	-4.385749	-0.372609	-0.251794
O	-2.320363	-1.101677	-0.002393
C	-0.621500	0.705411	-1.436123
H	-1.138016	-0.090198	-1.975455
H	0.054571	1.175357	-2.159697
H	0.347140	0.512287	-0.712734
C	-4.786140	1.965709	1.270091
H	-5.592256	1.795348	0.550842
H	-4.708955	1.056935	1.873300
H	-5.074945	2.793551	1.923141
K	-4.365874	-3.010974	-0.184499

6-3L

Pd	-0.944531	-0.396244	1.370075
C	-2.132772	-2.376663	-0.965525
C	-3.542348	-2.533472	-0.788605
C	-4.150733	-3.735095	-1.134525
C	-3.379001	-4.779009	-1.645077
C	-2.011402	-4.556843	-1.778478
N	-1.401042	-3.410111	-1.455330
H	-5.221440	-3.851580	-1.004229
H	-3.821297	-5.728161	-1.925966
H	-1.365064	-5.343538	-2.165549
O	-1.492997	-1.275830	-0.677118
C	-4.360823	-1.417357	-0.223346
F	-4.048601	-1.142237	1.072553
F	-5.688421	-1.695045	-0.238370
F	-4.207510	-0.255180	-0.908703
C	-2.323537	1.767733	-0.206900
C	-3.007664	2.985741	-0.426413
C	-3.679157	3.589399	0.625012
C	-3.667552	2.979686	1.882435
C	-2.944973	1.809491	2.036996
N	-2.255272	1.227927	1.030665
H	-4.217621	4.515751	0.459911
H	-4.198793	3.406897	2.724471
H	-2.893506	1.306889	2.995100
O	-1.740061	1.171552	-1.228075
C	-3.032638	3.566826	-1.810845
F	-1.790882	3.798655	-2.291561
F	-3.692306	4.750717	-1.837495
F	-3.653135	2.753122	-2.694949
C	3.923133	1.288787	2.287680
C	3.511788	-0.047473	2.152435
C	2.155266	-0.362944	2.395660
C	1.250993	0.648202	2.811000
C	1.707024	1.967435	2.943036
C	3.034117	2.288817	2.672376
H	4.955315	1.542916	2.063901
H	1.008604	2.740508	3.254173
H	3.375998	3.316956	2.759608
C	1.639846	-1.762541	2.124876
O	2.387180	-2.746010	2.222228
O	0.405555	-1.907869	1.710054
C	-0.179632	0.343602	3.092616
H	-0.318306	-0.478688	3.803259
H	-0.723167	1.228009	3.432865
H	-1.652873	0.163158	-1.070490
C	4.540083	-1.063430	1.711551
H	4.679628	-1.837043	2.470199
H	4.238300	-1.572624	0.792629
H	5.495428	-0.569587	1.518519
K	1.244812	-2.488975	-0.874872
C	3.398388	-0.272592	-1.477645
C	4.425142	0.747882	-1.507966
C	4.107375	2.075331	-1.261839
C	2.783677	2.436903	-0.994431
C	1.830526	1.416654	-1.001818
N	2.099005	0.131338	-1.232655
H	4.892275	2.826188	-1.275412
H	2.501637	3.463846	-0.790386
H	0.781601	1.647532	-0.814071
O	3.652060	-1.502264	-1.646297
C	5.825133	0.334759	-1.796156
F	6.338176	-0.531118	-0.878339
F	6.683444	1.396669	-1.819193
F	5.966568	-0.282180	-3.000736

11-ts-3L

Pd	0.937724	0.254941	1.436215
C	-2.268998	-1.391114	-1.464323
C	-2.174363	-2.715240	-1.973561
C	-3.319439	-3.336708	-2.449863
C	-4.535434	-2.648133	-2.425167
C	-4.534029	-1.358915	-1.909068
N	-3.441028	-0.733815	-1.436767
H	-3.261426	-4.349452	-2.833311
H	-5.450468	-3.100404	-2.789806
H	-5.460823	-0.788849	-1.866573
O	-1.169957	-0.818558	-1.017272
C	-0.862816	-3.439762	-1.931569
F	0.131303	-2.775067	-2.562076
F	-0.945429	-4.662013	-2.514521
F	-0.441846	-3.651365	-0.656570
C	-0.463217	2.358033	-0.448556
C	-0.615805	3.617752	-1.112119
C	0.492907	4.411683	-1.376055
C	1.755881	3.968888	-0.978674
C	1.843287	2.754704	-0.301395
N	0.780147	1.976315	-0.029768
H	0.369147	5.362026	-1.885941
H	2.648250	4.553591	-1.170945
H	2.794412	2.372911	0.064704
O	-1.473693	1.594588	-0.224810
C	-1.991211	4.059930	-1.473173
F	-2.669405	3.170578	-2.254226
F	-2.010914	5.239906	-2.129246
F	-2.793213	4.230411	-0.365566
C	-0.647456	-2.962751	2.346425
C	-1.879307	-2.289724	2.086998

C	-1.925493	-0.924630	2.319176
C	-0.763073	-0.202211	2.755042
C	0.426400	-0.912632	3.160105
C	0.430648	-2.321673	2.916483
H	-0.593229	-4.030413	2.149267
H	1.124619	-0.490479	3.883409
H	1.312691	-2.892311	3.195721
C	-3.137585	-0.053536	2.433806
O	-2.898977	1.087163	3.032677
O	-4.268359	-0.346640	1.991602
C	-0.994567	1.213353	2.918815
H	-1.187138	1.841749	2.063585
H	-0.585698	1.723974	3.791138
H	-1.311420	0.125592	-0.649795
C	-3.057675	-3.114707	1.632238
H	-3.945091	-2.500725	1.487821
H	-2.809611	-3.610688	0.686722
H	-3.283403	-3.907459	2.357114
K	-4.014235	1.630864	0.148755
C	3.919500	-0.074545	0.353514
C	4.936990	-0.625271	-0.525377
C	4.683073	-1.735304	-1.314195
C	3.424719	-2.347399	-1.269725
C	2.473772	-1.788564	-0.418884
N	2.687476	-0.714573	0.350169
H	5.462448	-2.121304	-1.965170
H	3.182555	-3.212788	-1.876395
H	1.479877	-2.221656	-0.348285
O	4.110613	0.934556	1.074869
C	6.258603	0.058681	-0.564611
F	6.179044	1.355233	-0.971642
F	7.129534	-0.546730	-1.424517
F	6.889190	0.086536	0.640010

7-ts-b-LP

Pd	1.205249	-1.764072	-0.328118
C	-1.815328	-0.897630	-0.351254
C	-3.225204	-1.143969	-0.384189
C	-3.718859	-2.431423	-0.286741
C	-2.820428	-3.499616	-0.162477
C	-1.464772	-3.215056	-0.163202
N	-0.959600	-1.964500	-0.250645
H	-4.789522	-2.605311	-0.312020
H	-3.163713	-4.524121	-0.084811
H	-0.732235	-4.013330	-0.089976
O	-1.365581	0.297349	-0.398613
C	-4.124632	0.036234	-0.561555
F	-3.956293	0.990640	0.394632
F	-5.429346	-0.302548	-0.550640
F	-3.900256	0.681896	-1.751295
C	-1.546281	1.905709	2.735502
C	-0.202643	2.184336	2.417046
C	0.665613	1.093929	2.253500
C	0.191497	-0.226847	2.457381
C	-1.172650	-0.474741	2.705720
C	-2.031523	0.603918	2.870500
H	-2.235803	2.739076	2.845038
H	-1.530615	-1.495095	2.796691
H	-3.083739	0.436608	3.075115
C	2.074526	1.184425	1.714264
O	2.551139	2.270323	1.301867
O	2.707732	0.075268	1.672333
C	1.098280	-1.334983	2.440176
H	2.001390	-1.340961	3.029471
H	0.724188	-2.315508	2.155904
H	0.035534	0.399910	-0.628104
C	0.217734	3.623302	2.235580
H	1.166552	3.830406	2.733345
H	0.370866	3.863135	1.179042
H	-0.549970	4.293156	2.634090
K	4.233373	-0.025154	-0.511914
O	2.197958	-3.450366	0.112941
O	3.098056	-2.472712	-0.271399
H	1.778752	2.768013	-0.177950
P	1.439612	1.542561	-1.970418
O	1.046809	0.371362	-0.849532
O	0.328969	1.641908	-2.985628
O	1.245467	2.865439	-1.004395
O	2.885890	1.311563	-2.324868
K	-1.657611	2.639255	-1.442246

7-ts-b-1L

Pd	0.464230	-0.782696	0.244281
C	-2.511997	0.024933	-0.038895
C	-3.886017	-0.158515	-0.280251
C	-4.329746	-1.415153	-0.666382
C	-3.408542	-2.456259	-0.807675
C	-2.074150	-2.204379	-0.544699
N	-1.631456	-0.980469	-0.161248
H	-5.384690	-1.576270	-0.856770
H	-3.721423	-3.447329	-1.113437
H	-1.310317	-2.970625	-0.644728
O	-2.093253	1.245903	0.316620
C	-4.831009	1.001985	-0.119835
F	-4.793257	1.510540	1.131622
F	-6.106001	0.635515	-0.365751
F	-4.531983	2.011851	-0.966673
C	2.492433	3.944559	-0.659907
C	3.174311	2.723071	-0.821818
C	2.817023	1.681031	0.044367
C	1.831911	1.863305	1.041613
C	1.132402	3.069977	1.150242
C	1.490243	4.116335	0.296725
H	2.737743	4.767747	-1.324408
H	0.359574	3.196056	1.902597
H	0.976115	5.069405	0.370456
C	3.285810	0.273538	-0.063096
O	4.230443	-0.138926	-0.726808
O	2.560370	-0.579107	0.680733
C	1.603148	0.748003	1.950805
H	2.400990	0.396618	2.594045
H	0.606892	0.611605	2.370945
H	-1.128935	1.239274	0.460339
C	4.208625	2.560109	-1.902710
H	5.193432	2.356178	-1.471631
H	3.973260	1.709645	-2.549392

H	4.273365	3.464180	-2.512473
K	3.420143	-3.087283	-0.104932
O	0.659231	-2.747653	0.738027
O	0.770879	-3.448615	-0.384035

7-ts-b-3L

Pd	-0.778341	-1.442454	-1.537666
C	0.797610	-0.237870	0.947611
C	0.928282	0.180711	2.307053
C	2.137284	0.699974	2.749962
C	3.202982	0.815224	1.856909
C	2.983435	0.416928	0.541215
N	1.831002	-0.093437	0.088704
H	2.239004	1.017888	3.782356
H	4.155080	1.235998	2.157175
H	3.770463	0.533355	-0.199487
O	-0.300374	-0.788871	0.521281
C	-0.249157	0.087754	3.220219
F	-1.269769	0.897438	2.833198
F	0.059369	0.441590	4.494289
F	-0.769894	-1.166985	3.287733
C	-3.214238	-0.099269	-0.127711
C	-4.333920	0.756847	-0.028608
C	-4.838892	1.350936	-1.177638
C	-4.217467	1.107166	-2.404460
C	-3.101977	0.282250	-2.432875
N	-2.624838	-0.316187	-1.320295
H	-5.692009	2.016822	-1.108146
H	-4.574583	1.568093	-3.318021
H	-2.504313	0.110509	-3.331928
O	-2.753655	-0.687220	0.962848
C	-4.887031	1.070081	1.330359
F	-5.338369	-0.028970	1.977706
F	-5.929660	1.933949	1.254798
F	-3.960095	1.644835	2.133071
C	2.592813	-3.664757	2.498038
C	2.963028	-3.320355	1.188335
C	1.946346	-3.312877	0.219161
C	0.626198	-3.663694	0.544546
C	0.270606	-3.958966	1.862981
C	1.271307	-3.968499	2.834941
H	3.352859	-3.660809	3.274645
H	-0.760576	-4.181466	2.118586
H	1.018274	-4.197014	3.866059
C	2.090563	-2.815544	-1.175746
O	3.145789	-2.502999	-1.723240
O	0.938242	-2.760188	-1.837222
C	-0.346652	-3.706315	-0.550341
H	-0.273793	-4.491871	-1.294286
H	-1.373881	-3.444789	-0.295777
H	-1.733386	-0.767847	0.919081
C	4.385734	-2.942790	0.869899
H	4.811967	-3.608910	0.113858
H	4.436099	-1.934205	0.451801
H	5.006279	-2.985947	1.769266
K	1.623132	0.158770	-2.935263
O	-1.184199	-1.837000	-3.426192
O	-0.812344	-0.668839	-4.038650
C	1.554647	2.842670	-1.473964
C	1.798482	3.496828	-0.203373
C	0.800427	3.576179	0.755269
C	-0.475839	3.076249	0.472965
C	-0.670209	2.520964	-0.791382
N	0.271194	2.396149	-1.730508
H	1.015157	4.028290	1.719262
H	-1.279825	3.099603	1.199575
H	-1.655621	2.146421	-1.062106
O	2.474382	2.642754	-2.320208
C	3.159403	4.038981	0.057400
F	4.151566	3.106417	0.001640
F	3.262405	4.608260	1.293553
F	3.523355	5.007076	-0.829110

PhCl

C	-1.575609	-1.208872	0.000002
C	-0.178749	-1.218790	0.000012
C	0.499460	-0.000007	-0.000016
C	-0.178747	1.218786	-0.000004
C	-1.575594	1.208883	0.000018
C	-2.275540	0.000000	-0.000013
H	-2.113345	-2.152461	0.000002
H	0.372657	-2.152778	0.000008
H	0.372683	2.152762	-0.000010
H	-2.113347	2.152460	0.000020
H	-3.361322	0.000016	-0.000025
Cl	2.267726	0.000000	0.000001

6-PhCl

Pd	0.112906	0.136922	-1.309931
C	1.187268	-2.682545	0.077473
C	2.419192	-3.029112	-0.560459
C	2.769193	-4.367786	-0.699405
C	1.914031	-5.363378	-0.226714
C	0.721453	-4.953406	0.358462
N	0.359748	-3.671105	0.506532
H	3.706878	-4.628909	-1.177953
H	2.160809	-6.414951	-0.317746
H	0.010174	-5.690696	0.728079
O	0.789714	-1.452865	0.276392
C	3.327392	-1.969511	-1.100260
F	2.773222	-1.288318	-2.140545
F	4.492894	-2.480133	-1.564843
F	3.661396	-1.041012	-0.168669
C	2.310502	1.414356	0.317516
C	3.253575	2.407450	0.671850
C	3.773026	3.235317	-0.309759
C	3.349460	3.072765	-1.632374
C	2.378602	2.123658	-1.898650
N	1.833106	1.333747	-0.945567
H	4.508040	3.988125	-0.048349
H	3.750717	3.680145	-2.434650
H	2.000951	1.979596	-2.903477
O	1.897058	0.581870	1.248138
C	3.687646	2.511186	2.105356
F	2.637545	2.722371	2.938715
F	4.545437	3.541116	2.290280

F	4.311649	1.394252	2.538475
C	-4.575388	2.852776	-1.359369
C	-4.393913	1.460699	-1.395665
C	-3.121500	0.949182	-1.759523
C	-2.083370	1.842378	-2.144110
C	-2.310136	3.225610	-2.080036
C	-3.544482	3.730262	-1.682515
H	-5.542510	3.247278	-1.060279
H	-1.509008	3.904444	-2.360223
H	-3.706497	4.803707	-1.634045
C	-2.840054	-0.530680	-1.637294
O	-3.766394	-1.351979	-1.506826
O	-1.605808	-0.953638	-1.586965
C	-0.759379	1.376550	-2.650813
H	-0.853912	0.719563	-3.523446
H	-0.112176	2.221496	-2.892521
H	1.488716	-0.279509	0.864996
C	-5.575468	0.600158	-1.007259
H	-5.875873	-0.058751	-1.825149
H	-5.341681	-0.056876	-0.165428
H	-6.424161	1.233850	-0.732282
K	-2.133873	-2.444204	0.611399
C	-1.671588	1.429022	1.903751
C	-2.936794	0.834417	1.869770
C	-3.210178	-0.202374	2.763959
C	-2.262797	-0.654097	3.686292
C	-1.006387	-0.040229	3.705384
C	-0.709702	1.000870	2.822198
H	-1.445155	2.225683	1.202257
H	-3.684626	1.163289	1.159187
H	-2.501808	-1.460569	4.371731
H	-0.260055	-0.382350	4.416060
H	0.270690	1.460927	2.836974
Cl	-4.793921	-1.000902	2.699781

10-PhCl

Pd	-1.965097	-1.402847	-0.034896
C	-1.100695	1.368786	-1.306309
C	-2.221484	2.115059	-1.735552
C	-2.288263	3.462967	-1.406379
C	-1.254953	4.035909	-0.660958
C	-0.203276	3.216402	-0.269534
N	-0.122148	1.907075	-0.571954
H	-3.138033	4.054695	-1.728240
H	-1.269747	5.084693	-0.388025
H	0.619073	3.619681	0.315461
O	-1.029471	0.074554	-1.639823
C	-3.304462	1.449072	-2.533375
F	-2.846600	0.936359	-3.696589
F	-4.298845	2.312722	-2.842828
F	-3.883309	0.420604	-1.857898
C	0.741786	-2.442301	-0.854966
C	1.812275	-3.386382	-0.818904
C	1.547758	-4.739374	-0.694595
C	0.220613	-5.174027	-0.594476
C	-0.778430	-4.215155	-0.570160
N	-0.539032	-2.890593	-0.680382
H	2.367309	-5.449773	-0.670735
H	-0.026567	-6.224974	-0.505378
H	-1.818927	-4.495005	-0.443068
O	0.976088	-1.184649	-1.016349
C	3.212289	-2.861767	-0.869458
F	3.483765	-2.159925	-1.994552
F	4.131389	-3.847802	-0.789250
F	3.474571	-2.011753	0.175424
C	-2.811493	1.934818	1.979873
C	-1.777658	1.277217	2.693204
C	-1.725456	-0.115875	2.702979
C	-2.737835	-0.859897	1.995761
C	-3.738562	-0.161995	1.252316
C	-3.768696	1.242916	1.260487
H	-2.834936	3.021596	1.991489
H	-4.540819	-0.722274	0.779722
H	-4.538350	1.771129	0.707987
C	-0.603088	-0.895394	3.404107
O	-0.952173	-1.745332	4.243126
O	0.572245	-0.594519	3.026276
C	-2.614000	-2.276478	1.714459
H	-1.853675	-2.844153	2.241671
H	-3.524095	-2.831041	1.481381
H	-0.097616	-0.399390	-1.384302
C	-0.751232	2.137964	3.394285
H	-0.080325	1.553800	4.022992
H	-0.139070	2.675955	2.658295
H	-1.245462	2.899572	4.008103
K	1.820770	0.303577	1.021001
C	3.709309	1.572194	-1.719851
C	3.287747	2.789047	-1.173000
C	3.824458	3.196563	0.050550
C	4.768873	2.425020	0.733261
C	5.181612	1.212840	0.168344
C	4.653897	0.783557	-1.054725
H	3.284063	1.236847	-2.660704
H	2.547608	3.398223	-1.679168
H	5.172018	2.763921	1.681546
H	5.913151	0.604484	0.691842
H	4.955454	-0.168681	-1.475495
Cl	3.288739	4.723291	0.761663

11-ts-PhCl

Pd	-2.952262	1.441903	-0.559299
C	-0.462582	-1.638502	1.372124
C	-1.087093	-2.764024	1.967816
C	-0.397148	-3.967069	2.009127
C	0.887833	-4.039548	1.463508
C	1.416848	-2.886599	0.897526
N	0.768587	-1.708275	0.845500
H	-0.860985	-4.837701	2.459321
H	1.456327	-4.962229	1.474000
H	2.410089	-2.901205	0.456005
O	-1.129945	-0.496079	1.348260
C	-2.469437	-2.634842	2.534622
F	-2.535299	-1.714432	3.525478
F	-2.905559	-3.806416	3.059763
F	-3.379690	-2.264898	1.601835
C	-0.406625	2.601897	0.570982

C	0.501395	3.608862	1.025782
C	0.030446	4.820007	1.505371
C	-1.349524	5.050517	1.533879
C	-2.188444	4.053665	1.053675
N	-1.752308	2.870373	0.576534
H	0.728558	5.574084	1.854294
H	-1.761308	5.980606	1.907233
H	-3.264902	4.197426	1.039565
O	0.017072	1.468243	0.145891
C	1.960584	3.300738	0.952189
F	2.304444	2.180301	1.642242
F	2.732666	4.301982	1.425592
F	2.379519	3.074096	-0.335839
C	-3.630325	-2.217813	-1.422761
C	-2.246863	-2.260339	-1.730252
C	-1.652829	-1.082590	-2.176849
C	-2.401232	0.115494	-2.288229
C	-3.819010	0.119914	-2.109133
C	-4.401895	-1.087985	-1.644170
H	-4.106598	-3.122503	-1.056545
H	-4.444897	0.912967	-2.517999
H	-5.476154	-1.130078	-1.489328
C	-0.267241	-0.908469	-2.725349
O	-0.092136	0.238894	-3.320305
O	0.629817	-1.761244	-2.588025
C	-1.634077	1.311477	-2.594617
H	-0.843547	1.662800	-1.951637
H	-1.992961	2.006986	-3.351453
H	-0.648800	0.236745	0.838572
C	-1.484029	-3.553548	-1.607873
H	-0.594483	-3.425732	-0.989424
H	-2.113198	-4.336904	-1.176261
H	-1.126955	-3.889984	-2.587201
K	1.941956	0.190477	-1.032410
C	4.743400	0.341898	1.191387
C	4.755510	-1.033674	0.939028
C	5.116209	-1.481216	-0.334963
C	5.465684	-0.590739	-1.353567
C	5.451444	0.782700	-1.081600
C	5.088917	1.251637	0.186035
H	4.445910	0.700210	2.171088
H	4.482970	-1.742397	1.713094
H	5.742416	-0.961446	-2.334632
H	5.721361	1.481858	-1.867477
H	5.058486	2.317572	0.385836
Cl	5.123465	-3.214570	-0.667533

3. Supplementary References

1. Frisch, M. J.; Trucks, G. W.; Schlegel, H. B.; Scuseria, G. E.; Robb, M. A.; Cheeseman, J. R.; Scalmani, G.; Barone, V.; Petersson, G. A.; Nakatsuji, H.; Li, X.; Caricato, M.; Marenich, A. V.; Bloino, J.; Janesko, B. G.; Gomperts, R.; Mennucci, B.; Hratchian, H. P.; Ortiz, J. V.; Izmaylov, A. F.; Sonnenberg, J. L.; Williams-Young, D.; Ding, F.; Lipparini, F.; Egidi, F.; Goings, J.; Peng, B.; Petrone, A.; Henderson, T.; Ranasinghe, D.; Zakrzewski, V. G.; Gao, J.; Rega, N.; Zheng, G.; Liang, W.; Hada, M.; Ehara, M.; Toyota, K.; Fukuda, R.; Hasegawa, J.; Ishida, M.; Nakajima, T.; Honda, Y.; Kitao, O.; Nakai, H.; Vreven, T.; Throssell, K.; Montgomery, J. A., Jr.; Peralta, J. E.; Ogliaro, F.; Bearpark, M. J.; Heyd, J. J.; Brothers, E. N.; Kudin, K. N.; Staroverov, V. N.; Keith, T. A.; Kobayashi, R.; Normand, J.; Raghavachari, K.; Rendell, A. P.; Burant, J. C.; Iyengar, S. S.; Tomasi, J.; Cossi, M.; Millam, J. M.; Klene, M.; Adamo, C.; Cammi, R.; Ochterski, J. W.; Martin, R. L.; Morokuma, K.; Farkas, O.; Foresman, J. B.; Fox, D. J., *Gaussian 09*, Revision D.01, Gaussian, Inc., Wallingford CT, **2009**.
2. Hay, P. J.; Wadt, W. R., Ab initio effective core potentials for molecular calculations. Potentials for K to Au including the outermost core orbitals. *J. Chem. Phys.* **1985**, *82*, 299-310.
3. Wadt, W. R.; Hay, P. J., Ab initio effective core potentials for molecular calculations. Potentials for main group elements Na to Bi. *J. Chem. Phys.* **1985**, *82*, 284-298.
4. Becke, A. D., Density-functional exchange-energy approximation with correct asymptotic behavior. *Phys. Rev. A* **1988**, *38*, 3098-3100.
5. Lee, C.; Yang, W.; Parr, R. G., Development of the Colle-Salvetti correlation-energy formula into a functional of the electron density. *Phys. Rev. B* **1988**, *37*, 785-789.

6. Becke, A. D., A new mixing of Hartree–Fock and local density‐functional theories. *J. Chem. Phys*. **1993**, *98*, 1372-1377.

7. Grimme, S.; Antony, J.; Ehrlich, S.; Krieg, H., A consistent and accurate ab initio parametrization of density functional dispersion correction (DFT-D) for the 94 elements H-Pu. *J. Chem. Phys.* **2010**, *132*, 154104.

8. Marenich, A. V.; Cramer, C. J.; Truhlar, D. G., Universal Solvation Model Based on Solute Electron Density and on a Continuum Model of the Solvent Defined by the Bulk Dielectric Constant and Atomic Surface Tensions. *J. Phys. Chem. B* **2009**, *113*, 6378-6396.

9. CYLview, 1.0b; Legault, C. Y., Université de Sherbrooke, **2009** (http://www.cylview.org).
